# Agreement and reliability between the two-day 6-minute incremental step test and two-day cardiopulmonary exercise test in post COVID-19 condition for assessing post-exertional malaise: The REVEAL-study

**DOI:** 10.1371/journal.pone.0353132

**Published:** 2026-07-14

**Authors:** Sarah Bomans, Naomi Michotte, Imane El M’Rabet, Berenice Jimenez Garcia, Lynn Leemans, Peter Janssens, Shane Hanon, Elisabeth De Waele, David Beckwée

**Affiliations:** 1 Clinical Nutrition Department, Universitair Ziekenhuis Brussel (UZ Brussel), Brussels Health Campus, Brussels, Belgium; 2 Faculty of Physical Education and Physiotherapy, Rehabilitation Research Group, Vrije Universiteit Brussel (VUB), Brussels Health Campus, Brussels, Belgium; 3 Faculty of Medicine and Pharmacy, Metabolism and Nutrition Research Unit, Vitality Research Group, Vrije Universiteit Brussel (VUB), Brussels Health Campus, Brussels, Belgium; 4 Pulmonology Department, Respiratory Division, Universitair Ziekenhuis Brussel (U.Z. Brussel), Brussels Health Campus, Brussels, Belgium; 5 Faculty of Medicine and Pharmacy, Lung Research Unit, Vitality Research Group, Vrije Universiteit Brussel (VUB), Brussels Health Campus, Brussels, Belgium; 6 Nephrology and Arterial Hypertension Department, Universitair Ziekenhuis Brussel (UZ Brussel), Brussels Health Campus, Brussels, Belgium; 7 Faculty of Medicine and Pharmacy, Kidney Diseases, Dialysis and Transplantation Research Unit, Vitality Research Group, Vrije Universiteit Brussel (VUB), Brussels Health Campus, Brussels, Belgium; Scuola Superiore Sant’Anna, ITALY

## Abstract

**Background:**

Post-Exertional Malaise (PEM) is a core symptom of post COVID-19 condition (also known as long COVID) affecting millions of people, yet assessment remains challenging. The two-day cardiopulmonary exercise test (CPET) is the current gold standard for objectifying PEM, but its cost and patient burden limit use. The two-day 6-minute incremental step test (6MIST) with wireless wearable sensors could offer a more accessible alternative.

**Methods:**

This cross-over study (n = 25, one-month washout period) evaluated the level of agreement and reliability between the two-day 6MIST and the two-day CPET for assessing PEM. Each “two-day” test consisted of two identical exercise tests separated by 24 hours to capture worsening of symptoms on day 2. Objective (VO_2_peak) and subjective (fatigue, neuromuscular complaints and rated perceived exertion) PEM outcomes were collected. Subjective outcomes were measured in relation to the first exercise test of each two-day session; at 15 minutes pretest, 15 minutes posttest and 24 hours posttest, with changes analyzed as Δ1 (baseline to 15 minutes posttest) and Δ2 (15 minutes posttest to 24 hours posttest). Agreement was assessed using Bland-Altman plots and reliability through consistency Intraclass Correlation Coefficients. The study is registered in ClinicalTrials.gov (Ref: NCT06933017).

**Results:**

VO_2_peak and neuromuscular complaints showed low agreement and reliability between the two tests. Rated perceived exertion showed moderate reliability at all times and fatigue showed moderate reliability for changes after 24h (Δ2).

**Conclusions:**

Contrary to our hypothesis, the two-day 6MIST shows limited agreement with the two-day CPET overall. However, moderate reliability for rated perceived exertion and fatigue suggests potential for improvement with protocol refinement. Further research is needed to optimize the two-day 6MIST and to develop assessments that capture PEM both within and beyond the 24-hour period.

## Introduction

The severe acute respiratory syndrome coronavirus (SARS-CoV-2) may belong to the past for many; however, it remains a daily struggle for people with post COVID-19 condition. Post COVID-19 condition, also known as post-acute sequelae of SARS-CoV-2 or long COVID, is a multisystemic condition following a SARS-CoV-2 infection that includes physical, cognitive and emotional impairments [1]. The number of affected individuals could reach up to 400 million worldwide [2] and in 2022, post COVID-19 condition contributed to an estimated reduction in labor-supply of 1 million workers in the European Union [2–4].

The World Health Organization (WHO) describes Post-Exertional Malaise (PEM) as a core symptom of post Covid-19 condition [5] with profound consequences on the overall wellbeing of the individual [6]. PEM is defined as “an abnormal response or exacerbation of symptoms following minimal physical, mental, cognitive, emotional or social activity that was previously well tolerated, usually manifesting 12-48 hours after activity and possibly lasting days to weeks” [7]. It often greatly affects an individual’s daily functioning and social life, potentially leading to further disability and reinforcing a negative cycle [8].

Objectifying PEM proves very challenging due to its substantial inter-individual variability in onset, symptom profile, and temporal evolution [9]. In the last decades, the number of studies exploring PEM has been rising in other populations suffering from PEM, namely myalgic encephalomyelitis (ME) and chronic fatigue syndrome (CFS). However, the mechanisms behind PEM remain unclear and there is no clear consensus on the best clinical strategy for managing PEM [10]. The emergence of post COVID-19 condition, with its strikingly similar reports of PEM, underscores the urgent need for further research.

Given the significant impact of PEM on patients with ME/CFS and post COVID-19 condition, reliable methods to objectively assess this phenomenon are essential. Currently, the most valid tool for assessing PEM in ME/CFS objectively is the two-day Cardiopulmonary Exercise Test (CPET), which triggers PEM under controlled conditions [11]. The two-day CPET protocol, involving two maximal exercise tests 24 hours apart, enables researchers to objectively quantify exertion-induced functional decline. Even though there is no clear cut-off for PEM, researchers can provide clear physiological evidence by showing abnormal recovery [12]. Building on ME/CFS research, our post COVID-19 condition approach adopts the two-day CPET to objectively characterize PEM. This can help to diagnose post COVID-19 condition and to provide patients with tangible evidence of their condition, validating their symptoms as physiological rather than psychosomatic, thereby enhancing clinical recognition [13].

Because of the high intensity, the two-day CPET can trigger severe or prolonged symptom exacerbation in patients with PEM [14]. This highlights the substantial physical burden this test can impose. Combined with its high costs, specialized equipment and personnel requirements, it makes the test impractical for large scale research [15]. Therefore, considering the high number of individuals affected by post COVID-19 condition suffering from PEM, the exploration of more accessible, affordable and patient-friendly assessment tools is necessary.

Field-based exercise tests, such as the incremental shuttle walking test or 6-Minute Walk Test, have been widely used as practical alternatives or complements to CPET in various clinical populations. These tests have demonstrated good reliability and clinical utility, especially in cardiovascular and cardiopulmonary disorders, where they are commonly used to assess functional capacity and guide treatment decisions [16–20]. In cardiovascular rehabilitation populations, incremental field tests have been shown to provide reproducible and clinically interpretable measures of functional capacity over time, supporting their use in both assessment and longitudinal follow-up [16,17]. These field-based tests are valuable due to their accessibility, low cost and ease of implementation.

Recent advances in wearable sensor technologies further enhance the potential of field- and home-based exercise testing by enabling continuous, objective monitoring of physiological responses such as heart rate and oxygen consumption in real-world environments [21,22]. This is particularly significant in light of the COVID-19 pandemic, which has accelerated the implementation of telemonitoring and remote rehabilitation strategies [23]. Evidence from cardiac rehabilitation and telehealth research demonstrates that remote assessment and monitoring can be safe and effective, while reducing patient burden and improving accessibility to care [24]. In individuals with post COVID-19 condition, who experience pronounced fatigue and PEM, the ability to perform functional assessments in a home-based setting may be particularly valuable.

Building on the established use of field-based functional tests, one promising option is the 6-minute incremental step test (6MIST) with wearable wireless sensor devices [15]. The 6MIST is a maximal exercise test which involves high knee stepping, with a metronome guiding the rhythm and accelerating every 30 seconds [15]. It’s important to note that the 6MIST is designed to be a patient-friendly test that can be performed with minimal equipment in many settings, both in and outside the hospital. Because of these advantages, the two-day 6MIST represents a possible alternative to the two-day CPET.

In this study, we compare the two-day CPET with the two-day 6MIST to measure PEM in an objective (VO_2_ peak) and subjective way (patient reported). This was done by 1) comparing the difference in VO_2_peak between day 1 and day 2 of both tests and by 2) comparing the difference in patient reported outcomes between day 1 and day 2 of both tests. If proven a good alternative to the two-day CPET, it could possibly be performed at the patient’s home in the future. Home monitoring could substantially reduce patient burden and eliminate the risk of inducing PEM due to traveling to and from the hospital. The 6MIST offers also a more cost-effective approach as it is less expensive, requires fewer medical staff and has a shorter duration than the CPET [15]. Furthermore, it could facilitate the longitudinal monitoring of PEM over time.

## Materials and methods

### Ethical approval

This study was approved by the Medical Ethics Committee of the University Hospital Brussels on December 4, 2024, before study initiation on March 20, 2025, but retrospectively registered in ClinicalTrials.gov (Ref: NCT06933017) on April 17, 2025. The delay in registration was due to fixed logistical constraints, including pre-scheduled access to cardiopulmonary exercise testing facilities, which required the study to start within a limited time window. The trial was registered in ClinicalTrials.gov as soon as possible, and all outcomes and the study protocol were defined a priori and remained unchanged during this period. The authors confirm that all ongoing and related trials for this intervention are registered. The study has been performed according to the Declaration of Helsinki. All participants provided written informed consent prior to undergoing any procedures. The recruitment period for this study started on March 20, 2025, and ended on July 3, 2025.

### Aim of the study

The aim of this study was to investigate if the two-day 6MIST is a reliable alternative to the two-day CPET for assessing PEM in post COVID-19 patients. Objective and subjective PEM assessments were compared between the two exercise tests at multiple time points. Objective PEM assessment was based on the change in VO_2_peak between day 1 and day 2, while subjective experiences were measured by changes between day 1 and day 2 in fatigue (Revised Piper Fatigue Scale (RPFS) [25]), Rate of Perceived Exertion (RPE [26]) and neuromuscular complaints (Likert scale [27]). Importantly, this study did not aim to establish absolute equivalence of outcome values between the two-day 6MIST and two-day CPET. Instead, the focus was on their concordance in detecting within-subject change over time, specifically the extent to which both protocols elicit a comparable decline in physiological capacity and symptom burden from day 1 to day 2 as an indicator of post-exertional malaise.

### Study trajectory

**Fig 1:**
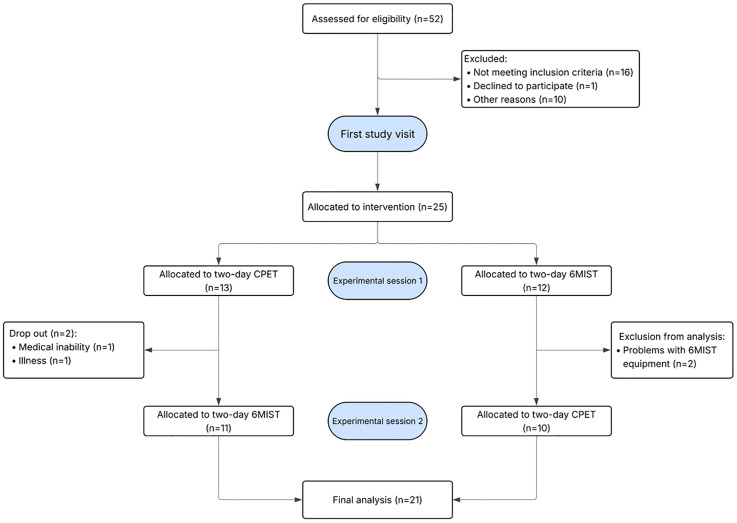
Study design REVEAL-study.

The trial was designed as a cross-over study with a washout period of 4 weeks (± 10 days). Half of the participants started with the two-day CPET and the other half with the two-day 6MIST, without random allocation due to logistical constraints. The study design is shown in Fig 1.

A total of 25 participants suffering from post COVID-19 condition were included in this study. Inclusion criteria were: ≥ 18 years old, suffering from post COVID-19 condition according to the WHO criteria (“the continuation or development of new symptoms three months after initial SARS-CoV-2 infection, with these symptoms lasting for at least two months with no other explanation”) [28], previously physically active as defined by the WHO criteria (“minimal 150 minutes/week of moderate intensity aerobic physical activity OR minimal 75 minutes of vigorous intensity activity/week OR an equivalent combination of moderate and vigorous intensity activity throughout the week”) [29], suffering from PEM as determined by the DePaul Symptom Questionnaire-PEM subscale (DSQ-PEM) [30], being capable to understand and provide written informed consent in Dutch, French or English. Exclusion criteria included experiencing symptoms that can be explained by pre-existing conditions or newly diagnosed medical conditions, having contra-indications to perform a CPET as determined by the medical staff, suffering from chronic obstructive pulmonary disease (COPD) Global Initiative for Chronic Obstructive Lung Disease (GOLD) [31] classification category 2–4, and pregnancy. Data collection was managed through the validated electronic data capture system REDCap.

### Recruitment

Recruitment took place via the Universitair Ziekenhuis (UZ) Brussel Clinical Nutrition team’s database (>150 post COVID-19 patients), established pre-study through prior research [32], clinic referrals, and media attention.

### Prescreening & screening

Potential participants were prescreened by telephone during which in- and exclusion criteria were verified through questions and the DSQ-PEM. When eligibility was initially established, the final screening was conducted by a medical doctor (MD) who verified the participants’ medical records and ensured that no contraindications existed for the exercise tests. If declared eligible by the medical doctor, patients were invited for a first study visit. In case the MD could not decide on eligibility solely based on the medical file, the patient was invited for a post COVID-19 condition consultation.

### First study visit

Before any procedure was carried out, written informed consent was obtained from the participant. The first study visit consisted of anamnesis, a brief clinical examination and a lung function test. The lung function test was performed to exclude alternative diagnoses and to ensure that participants started the exercise tests with normal baseline pulmonary function. During the anamnesis the study researcher collected demographic data (date of birth, gender, ethnicity, educational level, employment status, smoking, alcohol consumption, leisure time and sports) and listed symptoms (current and past) and medication. The clinical examination consisted of measuring height, weight, blood pressure (BP) and resting heart rate (HR). Height and weight were measured using a calibrated scale and measuring rod, both being periodically recalibrated by the biotechnical department. The lung function test was performed by a study nurse at the Pneumology Department of the UZ Brussel and is part of standard procedure before a CPET.

### Experimental sessions

Each participant completed two experimental sessions. Each session involved either a two-day CPET or a two-day 6MIST protocol, with a 24-hour interval (±2 hours) between the two testing days. To measure PEM subjectively the RPFS, BORG RPE and a 10-point Likert scale for neuromuscular complaints were taken at several time points: 15 minutes (± 15 minutes) before the first exercise test, 15 minutes (± 15 minutes) after the first exercise test and 24 hours (±2 hours) after the first exercise test but before the second exercise test.

Due to the intensive nature of the study, participants needed enough time to recover. Hence, a washout period of one month (4 weeks ± 10 days) was implemented. By giving enough recovery time, we aimed to minimize the dropout rate of the study and to limit probable interference between the two experimental sessions. The two-day CPET and two-day 6MIST were administered by the same study researcher, with a pulmonologist present during the CPETs.

### Questionnaires

#### Post exertional malaise – DSQ-PEM.

The DSQ-PEM is the most common and validated questionnaire to determine if a person suffers from PEM. Only Part 1 of the questionnaire was administrated, which scores every item on a scale from 0 to 4 for frequency and severity of PEM over the last six months [30]. A score of at least 2 for both frequency and severity on any item indicates the presence of PEM [30]. The supplementary yes/no items contained in Part 2 were not included in the present study.

#### Fatigue assessment – RPFS.

The RPFS is a self-assessment tool widely used across the world to measure fatigue. It assesses the physical and mental aspects of fatigue, including its characteristics and the impact on daily living [33]. This instrument is designed to reliably capture the subjective experience of fatigue in patients and has been validated in several languages, including the ones used in our study (English, Dutch [34] and French [35]. The scale contains 22 items and covers four dimensions of fatigue [36]. Each of the 22 items is given a score between 0 and 10, with 0 indicating the optimal condition and 10 the worst symptoms severity.

#### Neuromuscular complaints – 10-point Likert scale.

Neuromuscular complaints were assessed on a 10-point Likert scale [27], covering a wide range of symptoms such as joint pain, muscle pain and muscle soreness. The scale ranges from 1 to 10 with higher scores indicating more severe complaints. This single-item global rating was used as a practical indicator of neuromuscular complaint severity and was administrated in English, Dutch and French.

#### Rating of perceived exertion – BORG scale.

The BORG RPE scale is a validated tool that reliably reports the subjective perception of effort during an activity [26]. The BORG RPE is used in our study as a momentary self-report of perceived fatigue rather than exercise-specific effort. The scale has been translated and validated into different languages including Dutch [37], French [38] and English [26]. Values range from 6 to 20, where a score of 6 indicates very light perceived exertion and 20 represents maximal effort.

### CPET protocol

The CPET was performed in a standardized way on an electronically braked cycle ergometer, at the Pneumology Department of the UZ Brussel in accordance with current American Thoracic Society/European Respiratory Society (ATS/ERS) guidelines [39,40]. Prior to every CPET, a spirometry test was performed to assess Forced Expiratory Volume in 1 second (FEV_1_), Forced Vital Capacity (FVC) and to calculate Maximum Voluntary Ventilation (MVV). An individualized, symptom-limited ramp protocol was used, with the work rate increased stepwise to achieve volitional exhaustion within a target duration of 8–12 minutes. Participants maintained a pedaling cadence of 60–70 rotations per minute. The test was terminated at volitional exhaustion or earlier if predefined clinical stopping criteria were met (in accordance with ATS/ERS guidelines). During exercise, a continuous 12-lead electrocardiogram was recorded, and BP was measured manually at regular intervals. Breath-by-breath gas exchange variables were continuously collected using a calibrated metabolic cart.

### 6MIST protocol

The 6MIST is an incremental pace stationary stepping test, in which the pace is increased by 5, 10 or 15 steps every 30 seconds [15]. Participants were instructed to perform high knee lifts above waist. The rhythm was increased and guided by a metronome. If the patient could not follow the rhythm of the metronome, he or she was instructed to continue at his or her own maximal pace. The entire data collection process took ten minutes, with one minute data collection at rest, one minute warm-up, six minutes of stepping and two minutes of recovery. During the test, clinical cardiorespiratory variables were continuously measured. Measurement of VO_2_, ventilation (VE) and Respiratory Exchange Ratio (RER) was done with a portable metabolic analyser, Calibre™ Biometric tracker (Calibre biometrics, United States). HR was measured using the Polar Sense of Polar 10 HR monitor. All sensors (Calibre, Polar) were simultaneously linked to the SplendoMonitor app (SplendoHealth, USA) on an Apple iPad and continuously collected the data.

### Statistical analyses

The normality of data was assessed using the Shapiro-Wilk test. The Wilcoxon signed-rank test was employed to assess differences in peak wattage between day 1 and day 2 for the two-day CPET, as the data did not meet the normality assumption required for parametric tests. To assess differences between day 1 and day 2 in peak VO_2_ for the two-day CPET and the two-day 6MIST, a paired t-test (two-tailed) was used since the data was normally distributed. The intraclass correlation coefficient (ICC) evaluated reliability of day 1 minus day 2 outcomes between the two-day CPET and two-day 6MIST protocols. The ICC quantifies the consistency of repeated measurements within the same group; values closer to 1 indicate stronger reliability. We selected the ICC(3,1) model, corresponding to a two-way mixed-effects, single-measurement, consistency model [41]. This model assumes fixed measurement conditions and evaluates whether the pattern of repeated scores remains stable across days [42]. Reliability is calculated from a single measurement and the cut off values from Koo et al. (2016) were used [41]. Based on the 95% confidence interval of the ICC estimate, values below 0.50, between 0.50 and 0.75, between 0.75 and 0.90 and above 0.90 indicate poor, moderate, good, and excellent reliability, respectively. Standard Error Measurements (SEM) were calculated by dividing the standard deviation (SD) of the difference between tests by √2 (SDdiff/ √2) [43]. The SEM estimates the amount of measurement variability expected in repeated assessments, with smaller SEM values indicating greater measurement precision. Furthermore, Bland-Altman plots were used to evaluate agreement between the two-day CPET and two-day 6MIST. By using Bland-Altman plots, we visually represented differences between the two types of tests plotted against their mean values, estimated an agreement interval within which most differences between the test are expected to lie and calculated the mean difference (bias). For the subjective PEM scores, both absolute and relative values were analyzed. Relative values were calculated as ratios to baseline (T0) to account for inter-individual variability, as some participants reported higher fatigue at baseline. Subjective PEM scores were assessed at three predefined timepoints; T0 (15 minutes pre-exercise, baseline), T1 (15 minutes post-exercise), and T2 (24 hours post-exercise). To quantify post-exertional changes, two delta scores were computed: Δ1 representing the change from baseline to 15 minutes post-exercise (T0 – T1), and Δ2 representing the change from 15 minutes to 24 hours post-exercise (T1 – T2). Because we expected the objective scores to decrease over time, change scores were calculated by subtracting the later timepoint from the earlier one. A larger Δ score reflects greater deterioration and loss of function. An overview of timepoints and deltas can be found in Table 1.

**Table 1:** Overview of timepoints and calculation of delta scores.

Timepoints	
T0	15 min before exercise test 1 (baseline)
T1	15 min post exercise test 1
T2	24 hours post exercise test 1
**Delta’s**	
Δ1	T0 – T1
Δ2	T1 – T2

As no published minimal clinically important difference (MCID) was available in the literature to perform a formal power calculation, we based our sample size on a previous study employing the 6MIST in comparison to a supine CPET. The sample size for the present study was chosen to be consistent with that employed in Molinger et al. (2024) [15] (n = 15), which investigated similar outcomes, with an additional 40% increase to account for potential dropouts due to the intensive study protocol. All statistical analyses were conducted using Statistical Package for the Social Sciences (SPSS) at significance level 0.05.

## Results

### Participants

Demographic data is shown in Table 2. Twenty-five post COVID-19 patients (mean age: 49 ± 10.3 years, body mass index (BMI): 26.7 ± 3.5 kg/m^2^) presented a wide variation of PEM symptoms with fatigue being the most reported complaint (96%), followed by cognitive problems (72%) and musculoskeletal pain (68%) based on the anamnesis during the first study visit. A minority of the participants had been hospitalized for acute COVID-19 (16%). Key mean lung function values are shown in Table 1. The mean score on the DSQ-PEM was 2.69 ± 0.72 for symptom severity and 2.61 ± 0.72 for symptom frequency. Both symptom severity and frequency were normally distributed (p = 0.124 and p = 0.189 respectively). All participants completed the first experimental session. However, one participant was withdrawn from the study due to indications of a co-existing condition during the CPET and was referred for further medical follow-up. Another participant decided to withdraw due to an illness unrelated to the study. Both participants completed the two-day CPET as first experimental condition. Due to calibration issues with the 6MIST equipment, the data of two other participants could not be used. Consequently, 21 participants were included in the final agreement analysis (Fig 1).

**Table 2:** Baseline characteristics of the participants (mean ± standard deviation).

Demographics	
Age (years)	49 ± 10.3
Sex (M/F)	7/18
BMI (kg/m^2^)	26.7 ± 3.5
**Clinical history**	
Hospitalization due to COVID-19 (%) No Yes, in ICU Yes, not in ICU	84 4 12
**Symptoms (%)**	
Fatigue	96
Cognitive	72
Muscle/joint pain	68
Headache & migraine	56
Sensory hypersensitivity	36
Gastrointestinal	36
Respiratory	32
Visual complaints	28
Cardiac	24
Thermoregulation	16
Balance & coordination	12
Sleep disturbances	12
Tingling & numbness	8
Hair loss	4
**Lung function (%predicted)**	
FEV_1_	104.24 ± 14.65
TLC	99.48 ± 11.80
DLCO_SB	93.68 ± 12.49
**PEM (DSQ-PEM)**	
Severity	2.69 ± 0.72
Frequency	2.61 ± 0.72

BMI: Body Mass Index, DLCO_SB: Diffusing Capacity of the Lung for Carbon Monoxide Single Breath, DSQ-PEM: DePaul Symptom Questionnaire for Post Exertional Malaise, FEV_1_: Forced Expiratory Volume in 1 second, ICU: Intensive Care Unit, TLC: Total Lung Capacity

### Objective outcomes

#### Peak oxygen uptake (VO_2_ peak).

There was no significant difference in VO_2_ peak between day 1 and day 2 for the two-day CPET (p = 0.089), nor for the two-day 6MIST (p = 0.361).

### Peak wattage

Peak wattage was measured as a secondary outcome, serving as an additional check on workload consistency and physiological comparability between the two CPETs. This measure was not available for the two-day step test because the protocol involved knee lifting without external resistance, making it impossible to quantify mechanical workload. Mean of the absolute values for peak wattage of the two-day CPET are 150 ± 39.55 on day 1 and 141.4 ± 45.73 on day 2 (Table 3). The Wilcoxon signed rank test revealed a significant difference in peak wattage between day 1 and day 2 for the two-day CPET (p = 0.009); SPSS does not provide a 95% CI for the Wilcoxon signed rank test.

**Table 3:** Mean of the absolute values and standard deviation for objective and subjective PEM outcomes.

Objective Outcomes	Test	Day 1	Day 2	Mean Day 1 minus Day 2 + 95% CI
VO_2_ Peak (ml/min)	CPET	1836.46 ± 396.82	1783.29 ± 492.52	53.17 ± 187.25 [-25.90 to 132.24]
	6MIST	1746.65 ± 489.83	1781.14 ± 702.26	−34.49 ± 438.79 [-234.23 to 165.24]
Peak Wattage	CPET	150 ± 39.55	141.4 ± 45.72	Not applicable
	6MIST	Not applicable		
**Subjective Outcomes**	**Test**	**Day 1**		**Day 2**
		**T0**	**T1**	**T2**
RPE BORG	CPET	11.24 ± 2.26	16.76 ± 2.47	12.16 ± 2.86
	6MIST	12.00 ± 2.69	15.13 ± 2.52	12.73 ± 2.55
Likert scale	CPET	5.40 ± 2.58	7.52 ± 1.66	6.80 ± 2.04
	6MIST	5.36 ± 2.46	6.91 ± 2.39	5.96 ± 2.12
				
RPFS	CPET	5.49 ± 1.94	6.81 ± 1.46	6.12 ± 1.89
	6MIST	6.01 ± 1.98	6.62 ± 1.79	6.39 ± 1.81

**Fig 2:**
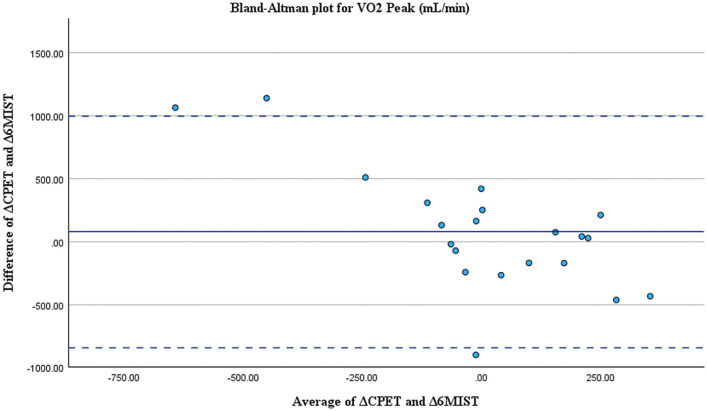
Bland-Altman plot for VO_2_peak values for the comparison of the two-day CPET and two-day 6MIST. Dotted lines represent the limits of agreement. Straight line represents the bias (mean difference). ΔCPET and Δ6MIST represent the differences in VO_2_ peak between day 1 and day 2.

The consistency between the change in VO₂peak measured by the two-day CPET and two-day 6MIST was very low with an ICC of 0.012 (95% CI [−0.413 to 0.432]; p = 0.479) (Table 4). The Bland-Altman plot for ΔVO_2_ peak values (two-day CPET vs two-day 6MIST) showed a mean difference of 76.92 mL/min, with wide limits of agreement ranging from −844.21 to 998,06 mL/min (Table 4). The wide limits of agreement point to large variability in the differences at the individual level (Fig 2).

**Table 4:** Agreement and reliability between the two-day CPET and two-day 6MIST for objective and subjective outcomes.

	ICC (3.1) 95% CI, single measure	SEM	Bias ± SD	Limits of agreement
**VO**_**2**_ **Peak**				
ΔVO_2_Peak (mL/min)	0.012 [-0.413 to 0.432]	332.08	76.92 ± 469.97	−844.21 to 998.06
**RPE**				
Δ1 Absolute	0.511 [0.123 to 0.763]	2.02	−2.50 ± 2.86	3.10 to −8.10
Δ1 Relative	0.518 [0.133 to 0.767]	0.23	−0.22 ± 0.32	0.42 to −0.85
Δ2 Absolute	0.715 [0.428 to 0.871]	1.55	2.68 ± 2.19	6.97 to −1.61
Δ2 Relative	0.613 [0.256 to 0.822]	0.19	0.24 ± 0.27	0.77 to −0.28
**10-point Likert scale**				
Δ1 Absolute	0.180 [-0.233 to 0.538]	1.63	−0.63 ± 2.30	3.88 to −5.13
Δ1 Relative	−0.070 [-0.470 to 0.353]	0.74	−0.16 ± 1.04	1.90 to −2.20
Δ2 Absolute	0.340 [-0.074 to 0.655]	1.29	−0.26 ± 1.82	3.30 to −3.82
Δ2 Relative	0.027 [-0.391 to 0.435]	0.52	−0.02 ± 0.73	1.45 to −1.42
**RPFS**				
Δ1 Absolute	0.498 [0.106 to 0.756]	1.03	0.73 ± 1.46	2.13 to −3.60
Δ1 Relative	0.245 [-0.198 to 0.605]	0.42	−0.30 ± 0.60	0.88 to −1.47
Δ2 Absolute	0.513 [0.137 to 0.760]	1.05	0.50 ± 1.48	3.40 to −2.38
Δ2 Relative	0.569 [0.193 to 0.800]	0.32	0.15 ± 0.45	1.04 to −0.74

CI: Confidence Interval, ICC: Intraclass Correlation Coefficient, RPE: Rate of Perceived Exertion, RPFS: Revised Piper Fatigue Scale, SD: Standard Deviation, SEM: Standard Error of Measurement

### Subjective outcomes

#### RPE measured by BORG.

**Fig 3:**
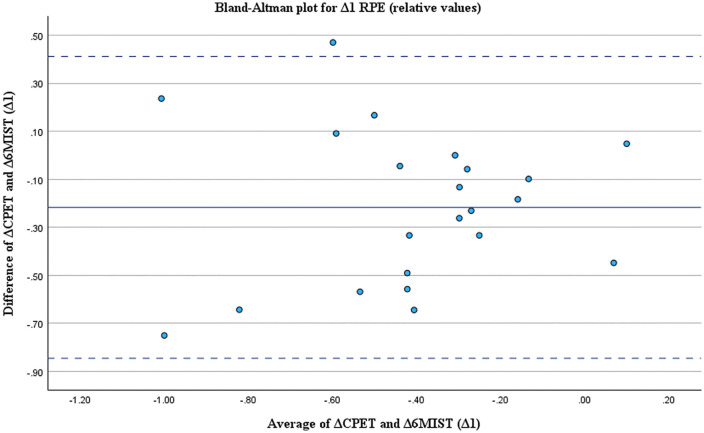
Bland-Altman plot for relative RPE BORG scores for Δ1 for the comparison of the two-day CPET and two-day 6MIST. Dotted lines represent the limits of agreement. Straight line represents the bias (mean difference). ΔCPET and Δ6MIST represent the differences in RPE BORG score between day 1 and day 2.

**Fig 4:**
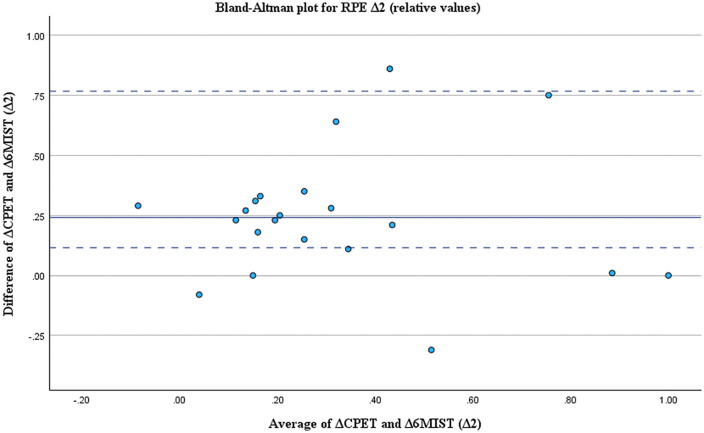
Bland-Altman plot for relative RPE BORG scores for Δ2 for the comparison of the two-day CPET and two-day 6MIST. Dotted lines represent the limits of agreement. Straight line represents the bias (mean difference). ΔCPET and Δ6MIST represent the differences in RPE BORG score between day 1 and day 2.

For Δ1 absolute values, the ICC showed moderate reliability (ICC = 0.511, 95% CI [0.123, 0.763]. The SEM was 2.02. The mean difference (bias) of −2.50 ± 2.86, indicates that the two-day CPET lead to a RPE score of 2.5 points lower than the two-day 6MIST. Limits of agreement ranged from 3.10 to −8.10, suggesting substantial variability in the responses of participants (Table 4). For Δ2 absolute values, the ICC indicated moderate reliability (ICC = 0.715, 95% CI [0.428, 0.871). The SEM was 1.55. The mean difference was 2.68 ± 2.19, indicating that the two-day CPET lead to a higher perceived exertion than the two-day 6MIST with wide limits of agreement from 6.97 to −1.61 (Table 4). At Δ1 for relative changes, the ICC showed moderate reliability (ICC = 0.518, 95% CI [0.133, 0.767] with a SEM of 0.23. The mean difference of −0.22 ± 0.32 was negligibly small and had narrow limits of agreement (0.42 to −0.85) (Table 4). At Δ2 for relative changes, reliability was moderate (ICC = 0.613, 95% CI [0.256, 0.822] with a SEM of 0.19. The mean difference was 0.24 ± 0.27 with limits of agreement ranging from 0.77 to −0.28 (Table 4). This points to good agreement in RPE. The Bland-Altman plots for relative values can be found in Fig 3 and Fig 4. The Bland-Altman plots for absolute values are provided in the S1 Appendix.

### Neuromuscular complaints measured by a 10-point Likert scale

**Fig 5:**
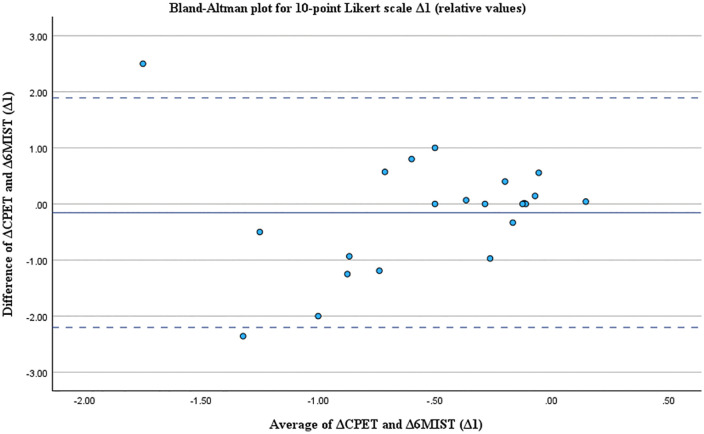
Bland-Altman plot for relative 10-point Likert scores for neuromuscular complaints for Δ1 for the comparison of the two-day CPET and two-day 6MIST. Dotted lines represent the limits of agreement. Straight line represents the bias (mean difference). ΔCPET and Δ6MIST represent the differences in 10-point Likert score between day 1 and day 2.

**Fig 6:**
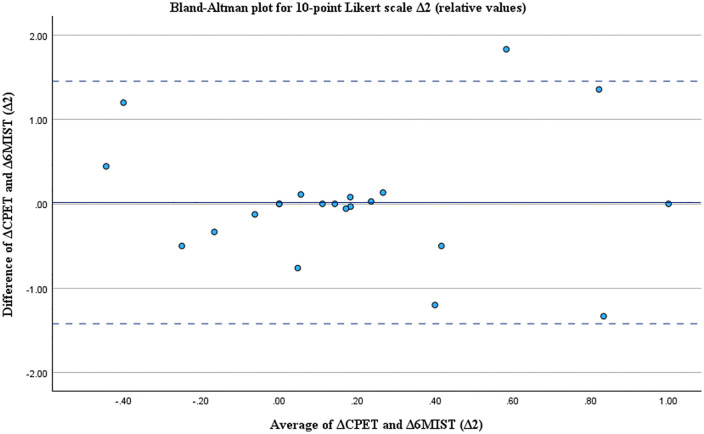
Bland-Altman plot for relative 10-point Likert scores for neuromuscular complaints for Δ2 for the comparison of the two-day CPET and two-day 6MIST. Dotted lines represent the limits of agreement. Straight line represents the bias (mean difference). ΔCPET and Δ6MIST represent the differences in 10-point Likert score between day 1 and day 2.

Poor reliability was found between the two-day CPET and the two-day 6MIST at all time points with very low ICCs (< 0.40) and even one ICC value below zero, indicating that participants perceived the neuromuscular demands of the two tests as fundamentally different. Relatively wide limits of agreement were observed as well, given the limited range of the scale itself (Table 4). The Bland-Altman plots for relative values are shown in Fig 5 and Fig 6, the Bland-Altman plots for absolute values are provided in the S1 Appendix.

### Fatigue (physical and mental) by the RPFS

**Fig 7:**
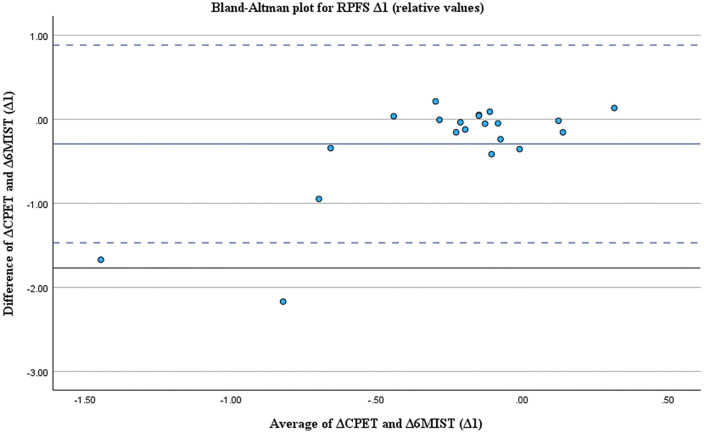
Bland-Altman plot for relative RPFS scores for fatigue for Δ1 for the comparison of the two-day CPET and two-day 6MIST. Dotted lines represent the limits of agreement. Straight line represents the bias (mean difference). ΔCPET and Δ6MIST represent the differences in RPFS score between day 1 and day 2.

**Fig 8:**
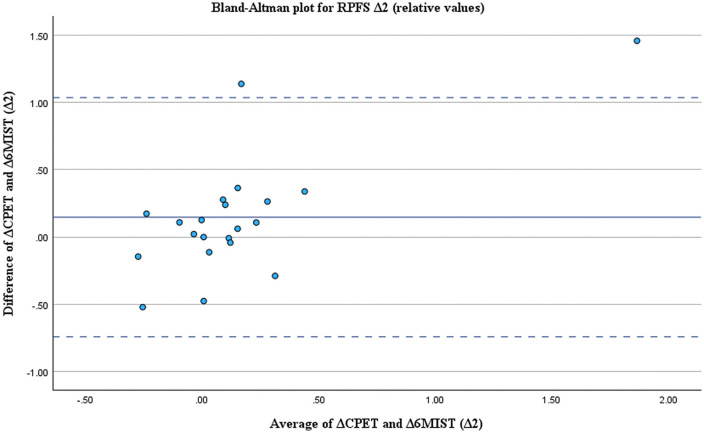
Bland-Altman plot for relative RPFS scores for fatigue for Δ2 for the comparison of the two-day CPET and two-day 6MIST. Dotted lines represent the limits of agreement. Straight line represents the bias (mean difference). ΔCPET and Δ6MIST represent the differences in RPFS score between day 1 and day 2.

At Δ1, the results showed an ICC for absolute measurements of 0.498 and limits of agreement ranging from 2.13 to −3.60 (Table 4), indicating rather poor reliability and agreement. The relative values had a very low ICC of 0.245 and low limits of agreement (0.88 to −1.47). Comparable findings were found at Δ2 showing moderate ICC values of 0.513 (absolute values) and 0.569 (relative values) with acceptable limits of agreement for relative values (1.04 to −0.74) (Table 4). The Bland-Altman plots for relative values are shown in Fig 7 and Fig 8, the Bland-Altman plots for absolute values are provided in the S1 Appendix.

## Discussion

Field-based exercise tests are increasingly studied as an accessible alternative to CPET in different population groups. A recent systematic review with meta-analysis showed a moderately strong association between the 6-minute walking test and CPET- particularly peak VO_2_ – and with excellent test-retest reliability, supporting their validity as practical alternative when CPET is not feasible [20]. In addition, another study has demonstrated significant and moderate associations between a new incremental step test and the 6-minute walking test in people with COPD [44]. However, although these findings suggest that field-based exercise tests are clinically useful in other populations, their ability to capture PEM-related physiological changes in post COVID-19 condition has not yet been established.

This study aimed to compare the two-day CPET and the two-day 6MIST as measures of PEM in individuals with post COVID-19 condition. Opposite to our hypothesis, we found overall low agreement and low to moderate reliability between the two-day CPET and two-day 6MIST for both objective and subjective outcomes. Only RPE and RPFS at Δ2 demonstrated moderate reliability (RPE: ICC = 0.511 [0.123 to 0.763]; 0.518 [0.133 to 0.767]; 0.715 [0.428 to 0.871] and 0.613 [0.256 to 0.822]; RPFS: ICC = of 0.513 [0.137 to 0.760] and 0.569 [0.193 to 0.800]) (Table 4) with moderate to good agreement for RPE (relative values). This indicates that the two-day 6MIST is currently not yet an optimal alternative to the two-day CPET to measure PEM. However, the two-day 6MIST remains a simpler, less resource-intensive test, and with further refinement of the protocol or in combination with other measures it may still provide valuable insights into PEM symptom changes.

### Objective outcomes

The two-day CPET peak wattage demonstrated a significant decrease from day 1 to day 2. This aligns with a meta-analysis comparing two-day CPET results at peak and first ventilatory threshold (VT) between individuals with ME/CFS and sedentary healthy controls [12]. In ME/CFS, mean values across all parameters declined from day 1 to day 2 of the CPET, whereas they rose in controls. The difference between the patient and control group was most significant at the wattage at the first VT [12].

Although we observed no statistically significant decrease in VO_2_peak between day 1 and day 2 for the two-day CPET and two-day 6MIST, several participants did not meet commonly used indicators of maximal effort. This may suggest that the tests were submaximal for some participants, possibly due to apprehension about inducing PEM the following day. The absence of significant decrease in VO_2_peak is inconsistent with a study with a large sample size of ME/CFS patients (N = 84) and sedentary controls (N = 71). The people suffering from ME/CFS had significant declines in VO_2_peak on day two of the two-day CPET, but not the control group [14]. This decline on day two persisted in aerobic capacity-matched case-control pairs, indicating that baseline fitness did not account for the difference [14]. In contrast, our study results are in line with two other studies that performed a two-day CPET in people with post COVID-19 condition [45,46]. Those studies reported no significant differences in physiological responses between day 1 and day 2 [45] and less frequent PEM following a CPET in post COVID-19 condition compared to the ME/CVS cohort [46]. This consistency suggests that the two-day CPET may not be the most suitable method for measuring PEM in individuals with post COVID-19 condition, as it is for people with ME/CFS.

Furthermore, as our study represents the first application of the two-day 6MIST—and the first use of the 6MIST in a PEM-affected population—no prior data exist on declines on day two for this protocol, precluding direct comparisons.

Functional capacity tests such as walking and stepping tests are increasingly used in clinical practice because they provide accessible and clinically meaningful information regarding exercise tolerance. Previous literature has shown that the interpretation of these tests is multifactorial-for instance, the 6-minute walking test result should take into account age, stature and BMI in individuals with chronic heart failure [19]. The 6MIST (but without a structured increase in exercise intensity), has been previously investigated and validated as an equivalent alternative to the standard 6-minute walk test in the same population [47]. A similar step test to the 6MIST has also been evaluated in patients with COPD. In this study, it was demonstrated that the step test was feasible and safe for home-based administration [44]. Moreover, Molinger et al. (2024), compared the 6MIST to the CPET with invasive hemodynamics in supine position in heart failure patients [15]. In contrast to our findings, they reported good to excellent ICCs between the invasive CPET and the 6MIST for peak HR, absolute VO_2_peak, relative VO_2_peak, maximal VE, oxygen pulse and cardiorespiratory optimal point. In the present study, however, the 6MIST was compared to the standard CPET protocol performed in a sitting position, since this reflects better real-life conditions. The CPET and 6MIST were conducted over two consecutive days with the aim of capturing a loss of functional capacity associated with PEM. These differences may explain why we failed to find good ICCs and agreement for most of the measurements.

### Subjective outcomes

Unlike Gattoni et al. [45], who measured subjective PEM symptoms in relation to the preceding six months with the DSQ-PEM, our study focused on PEM symptoms measured at the time of testing, which reflects more immediate and test-related symptom changes.

RPE showed moderate reliability at all times and RPFS at Δ2 for absolute and relative values, depicted by the ICCs. The relative values for BORG RPE showed better agreement than absolute values, which is shown through lower SEMs and tighter limits of agreement. The BORG RPE is developed to capture intensity and impact of exertion across a broad range of severities, which could explain our findings regarding reliability when symptoms fluctuate over time as for example with PEM [48]. The RPFS is a multidimensional instrument, containing 22 extensive questions that cover various domains of fatigue (behavioral, affective, sensory, and cognitive/mood dimensions) [33]. People with post COVID-19 condition experience fatigue beyond just the physical level [49], which makes the RPFS more accurate than a regular fatigue questionnaire asking only about the physical part and might reduce measurement errors. The RPFS has been validated for internal consistency and sensitivity in detecting fatigue-related differences [33] and the BORG RPE scale has been validated for exercise intensity scaling [50]. Regarding the 10-point Likert scale for measuring neuromuscular complaints, no meaningful agreement nor reliability between test modalities was found at any time point. The absence of meaningful findings might be explained by differences in physiological stress imposed by the different test protocols. The CPET is a well validated maximal effort test that stresses both the central and the peripheral systems [51]. The 6MIST elicits greater localized fatigue in the legs, likely due to the unfamiliarity of the high-knee stepping pattern. These differences in fatigue mechanisms might lead to different scoring on the questionnaires. Another possible reason is that the 10-point Likert scale may have been insufficiently specific for participants to reliably interpret the response options, which could have reduced its sensitivity to subtle changes in symptom severity. This might have resulted in differences in interpretation or shifts in how participants perceived pain which could have led to a larger random error and low consistency. The poor to moderate agreement in subjective scoring may also partly stem from contextual and motivational factors [52]. The CPET occurred in a more clinical setting with multiple study personnel and extensive amount of monitoring equipment present, potentially increasing symptom awareness. The 6MIST took place in a less medicalized setting, with only one researcher taking the test. Participants possibly perceived the test as “less serious” because of this.

### Strengths and limitations

Literature has shown that ME/CFS patients need about two weeks to recover from a two-day CPET, whereas sedentary controls recover within approximately two days [53]. We extended the washout period by an additional two weeks beyond the standard two-week recovery time following a two-day CPET [53] to ensure participants were fully recovered and to eliminate any residual effects from the initial test. We had a pragmatic sample size for a two-day exercise test protocol inducing PEM, even with two dropouts and two participants excluded from analysis due to calibration errors with the 6MIST equipment. However, no formal a priori power calculation was performed due to the absence of established MCIDs for the outcomes in this population. As such, the study may be underpowered to detect small differences between conditions. Non-significant findings, such as those from the non-significant Wilcoxon signed-rank and paired t-tests, should therefore be interpreted with caution. Another strength of our approach is that PEM was measured in real time, allowing for a more precise and immediate assessment compared to the classic method using the DSQ-PEM, which relies on retrospective recall over the past six months and is therefore subject to recall bias. A methodological limitation of the 6MIST was selecting the appropriate pacing level. The 6MIST requires judgement of the clinical profile of the participant to balance safety with sufficient exertion in order to attain maximal effort. This degree of subjectivity may have influenced the outcomes, resulting sometimes in a submaximal test. Another limitation is that both testing protocols weren’t fully equivalent. Participants underwent spirometry before each CPET but not before each 6MIST, which could have contributed to fatigue prior to the CPET.

### Directions for future research

The observed lack of agreement and reliability in the two-day 6MIST likely stems from methodological and protocol-related factors—rather than a lack of clinical relevance—while its potential applicability in home-based settings remains clinically meaningful despite these limitations. The COVID-19 pandemic accelerated the implementation of home-based telerehabilitation, where remote functional assessments have gained adoption [24]. A recent study demonstrated that wearable technologies and remote assessment methods are feasible for measuring functional capacity beyond rehabilitation or hospital settings [54]. Home-based assessment methods may be particularly valuable in individuals with post COVID-19 condition, who frequently suffer from fatigue and PEM because of travelling and hospital-based testing. Therefore, while the two-day 6MIST protocol would benefit from further refinement and validation, its accessibility and feasibility suggest potential for exploration as a home-based assessment tool.

Given that the 6MIST is straightforward and easy to administer, it remains a promising tool, and further investigation is warranted to refine protocols and improve reproducibility and clinical utility. Future studies should focus on establishing standardized criteria for test execution, particularly to ensure that participants reach maximal rather than submaximal exertion. Another potential direction for future research is to focus on measuring and comparing exercise intensity using wattage instead of VO₂peak. In the present study, wattage demonstrated significant differences between test days, whereas VO_2_peak did not, suggesting that wattage may be a more sensitive indicator of changes associated with PEM in post COVID-19 condition. In addition, alternative outcome measures may be worth exploring. Although the two-day CPET is currently considered the reference method for objectively assessing PEM, it may not fully capture the delayed onset of symptom exacerbation, which in our population often occurred several days after exertion (based on a feedback conversation). This observation, together with the absence of a significant decline in VO_2_peak, may indicate that the two-day CPET might not be sufficiently sensitive to detect PEM-related changes in individuals with post COVID-19 condition, unlike in ME/CFS patients [12], although this requires further investigation. Future research should therefore focus on developing assessments that better capture the timing and multidimensional nature of PEM, including measurements beyond 24h post-exercise.

## Conclusion

To our knowledge, this is the first study to evaluate agreement and reliability between the two-day CPET and the two-day 6MIST for assessing PEM in individuals with post COVID-19 condition. The current findings indicate poor agreement and low reliability for objective outcomes and predominantly poor agreement across subjective outcomes, despite moderate reliability for fatigue and RPE. Despite its cost and complicated set-up, the two-day CPET remains the best option to measure PEM in post COVID-19 condition at present. Nevertheless, the two-day 6MIST shows potential for clinical applicability, and further refinement is warranted to improve its reliability and agreement as well as to better capture PEM across extended time frames.

## Supporting information

S1 Appendix Bland-Altman plots absolute values. Bland-Altman plots for absolute values for RPE by BORG, neuromuscular complaints by a 10-point Likert-scale and for fatigue (physical and mental) by the RPFS.
(PDF)

S2 File TREND statement checklist. Completed TREND checklist with corresponding manuscript page numbers for each reporting item.
(DOCX)

S3 File Clinical trial protocol. Final approved study protocol.
(PDF)

## Abbreviations

6MIST: 6-Minute Incremental Step Test
ATS: American Thoracic Society
BMI: Body Mass Index
BP: Blood Pressure
CFS: Chronic Fatigue Syndrome
COPD: Chronic Obstructive Pulmonary Disease
CPET: CardioPulmonary Exercise Test
DLCO: Diffusion Capacity of the Lung for Carbon Monoxide
DSQ-PEM: DePaul Symptom Questionnaire for Post-Exertional Malaise
EC: Ethics Committee
ERS: European Respiratory Society
FEV1: Forced Expiratory Volume in 1 second
FVC: Forced Vital Capacity
GOLD: Global Initiative for Obstructive Lung Disease
HR: Heart Rate
ICC: Intraclass Correlation Coefficient
MCID: Minimal Clinically Important Difference
MD: Medical Doctor
ME: Myalgic Encephalomyelitis
MVV: Maximal Voluntary Ventilation
PEM: Post-Exertional Malaise
PESE: Post-Exertional Symptom Exacerbation
RCT: Randomized Controlled Trial
RER: Respiratory Exchange Ratio
RPE: Rate of Perceived Exertion
RPFS: Revised Piper Fatigue Scale
SARS-CoV-2: Severe Acute Respiratory Syndrome Coronavirus
SD: Standard Deviation
SEM: Standard Error Measurement
SPSS: Statistical Package for the Social Sciences
TLC: Total Lung Capacity
VE: Ventilation
VT: Ventilatory Threshold
VUB: Vrije Universiteit Brussel
UZ: Universitair Ziekenhuis
