# Supplementary material for: Agreement and reliability between the two-day 6-minute incremental step test and two-day cardiopulmonary exercise test in post COVID-19 condition for assessing post-exertional malaise: The REVEAL-study
Source: PLoS One. 2026 Jul 14;21(7):e0353132. doi: 10.1371/journal.pone.0353132 (PMC13367738; doi:10.1371/journal.pone.0353132)
Supplement: S1 Appendix — Bland-Altman plots for absolute values for RPE by BORG, neuromuscular complaints by a 10-point Likert-scale and for fatigue (physical and mental) by the RPFS. (PDF) [file pone.0353132.s001.pdf]

Supplementary Materials

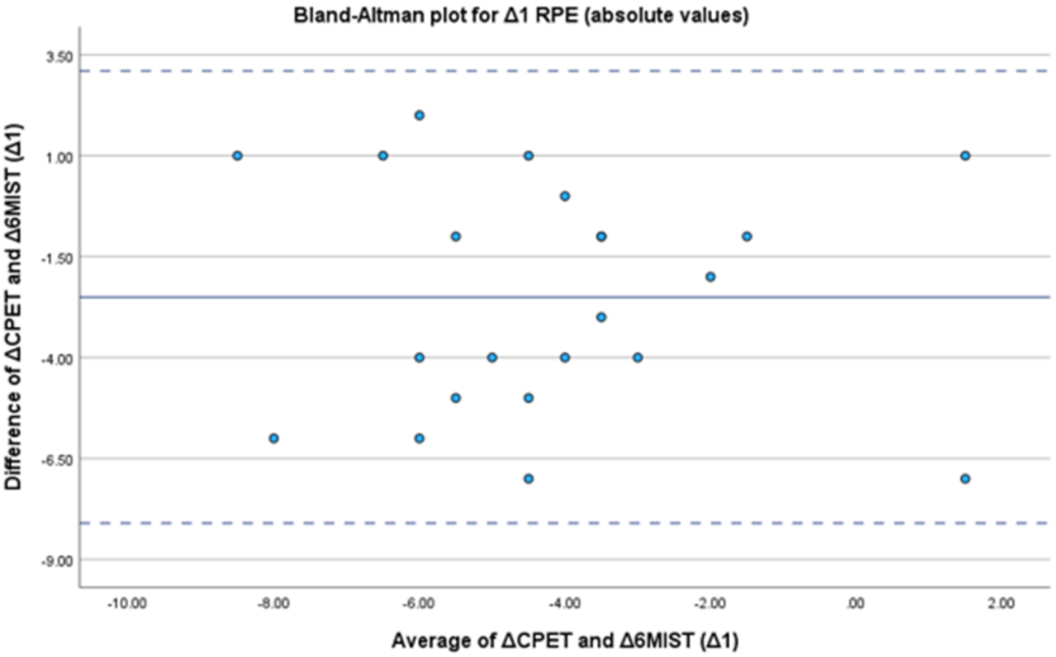

Fig S1. Bland-Altman plot for absolute RPE BORG scores for Δ1 for the comparison of the two-day CPET and two-day 6MIST. Dotted lines represent the limits of agreement. Straight line represents the bias (mean difference). ΔCPET and Δ6MIST represent the differences in RPE BORG score between day 1 and day 2.

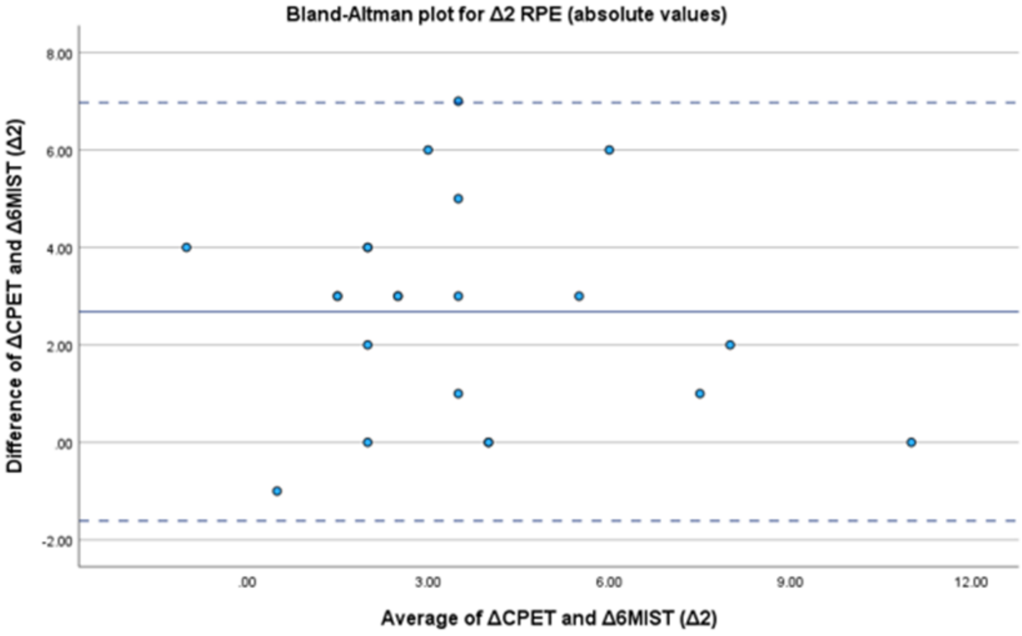

Fig S2. Bland-Altman plot for absolute RPE BORG scores for Δ2 for the comparison of the two-day CPET and two-day 6MIST. Dotted lines represent the limits of agreement. Straight line represents the bias (mean difference). ΔCPET and Δ6MIST represent the differences in RPE BORG score between day 1 and day 2.

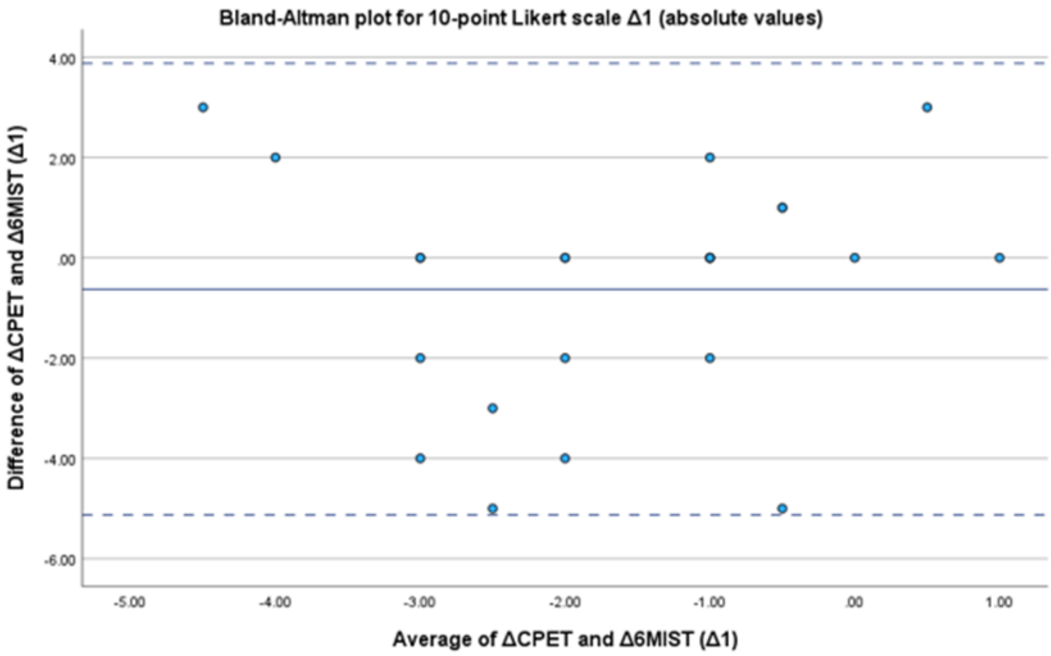

Fig S3. Bland-Altman plot for absolute 10-point Likert scores for neuromuscular complaints for Δ1 for the comparison of the two-day CPET and two-day 6MIST. Dotted lines represent the limits of agreement. Straight line represents the bias (mean difference). ΔCPET and Δ6MIST represent the differences in 10-point Likert score between day 1 and day 2.

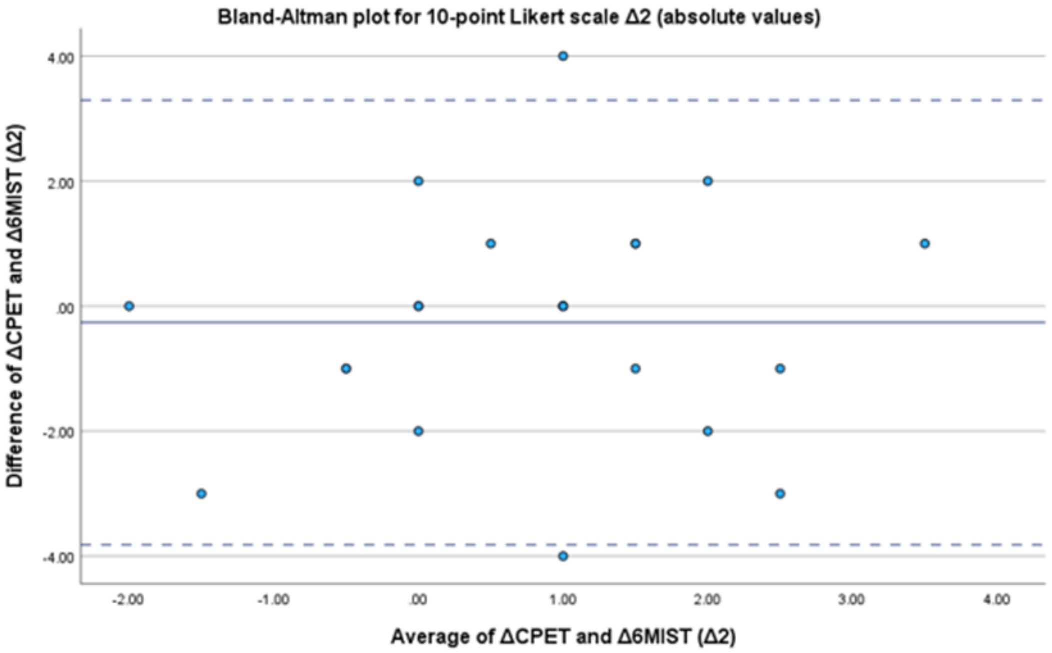

Fig S4. Bland-Altman plot for absolute 10-point Likert scores for neuromuscular complaints for Δ2 for the comparison of the two-day CPET and two-day 6MIST. Dotted lines represent the limits of agreement. Straight line represents the bias (mean difference). ΔCPET and Δ6MIST represent the differences in 10-point Likert score between day 1 and day 2.

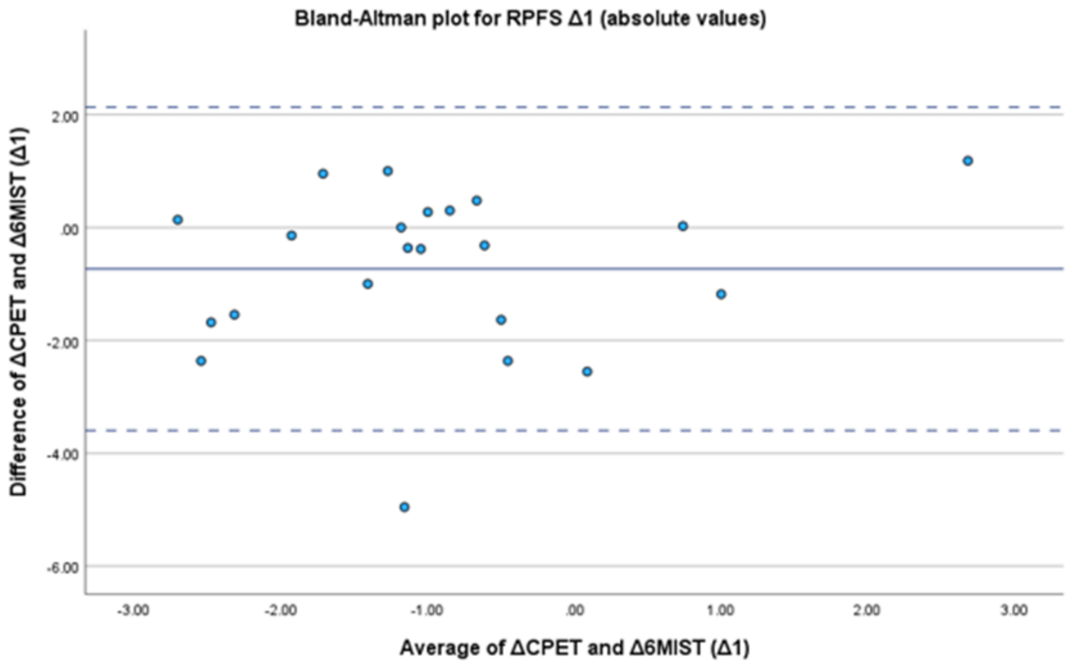

Fig S5. Bland-Altman plot for absolute RPFS scores for fatigue for Δ1 for the comparison of the two-day CPET and two-day 6MIST. Dotted lines represent the limits of agreement. Straight line represents the bias (mean difference). ΔCPET and Δ6MIST represent the differences in RPFS score between day 1 and day 2.

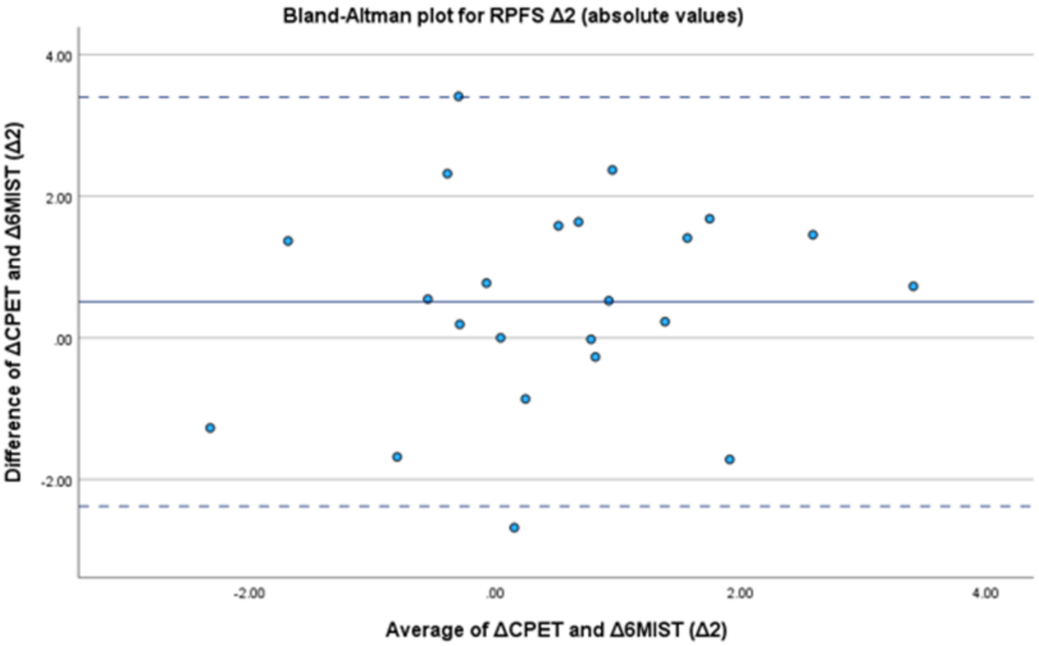

Fig S6. Bland-Altman plot for absolute RPFS scores for fatigue for Δ2 for the comparison of the two-day CPET and two-day 6MIST. Dotted lines represent the limits of agreement. Straight line represents the bias (mean difference). ΔCPET and Δ6MIST represent the differences in RPFS score between day 1 and day 2.
