# Supplementary material for: Agreement and reliability between the two-day 6-minute incremental step test and two-day cardiopulmonary exercise test in post COVID-19 condition for assessing post-exertional malaise: The REVEAL-study
Source: PLoS One. 2026 Jul 14;21(7):e0353132. doi: 10.1371/journal.pone.0353132 (PMC13367738; doi:10.1371/journal.pone.0353132)
Supplement: S3 File — Final approved study protocol. (PDF) [file pone.0353132.s003.pdf]

Study Protocol

**Study Title: Feasibility study** of the augmented Two-Day 6-Minute Incremental Step Test and causal exploration through a Directed Acyclic Graph and Structural Equation Model for Post-Exertional Symptom Exacerbations in long COVID Patients
**Study Acronym: REVEAL** (**R**eliable **EV**aluation of Feasibility for **E**xertion **A**nalysis in **L**ong COVID)<br><br>**Protocol Version and Date: V2 07/11/2024**<br><br>**Sponsor:** UZ Brussel<br><br>**Protocol number:** 2024-EDW-REVEAL<br><br>**Principal Investigator:** Elisabeth De Waele

The information contained in this document is the property of the Sponsor/Coordinating Investigator and may not be reproduced, published or disclosed to others without written authorization of the Sponsor/Coordinating Investigator.

PROTOCOL SIGNATURE PAGE

I agree:
- to assume responsibility for the proper conduct of this study
- to conduct the study in compliance with this protocol
- not to implement any deviations from or changes to the protocol without prior review and written approval from the Ethics Committee, or for administrative aspects of the study (where permitted by all applicable regulatory requirements)
- to ensure that all persons assisting me with the study are adequately informed about their study-related duties and functions as described in the protocol
- that I am aware of and will comply with the current good clinical practice (GCP) guidelines and ethical principles outlined in the Declaration of Helsinki
- to conduct the study in accordance with all applicable laws and regulations

Printed name   Signature   Date

Table of Contents

1 Sponsor/Coordinating Investigator Information 3

2 List of Abbreviations 3

3 Protocol Version History 4

4 Trial Registration/Protocol Summary 4

5 Background and Rationale 5

5.1 Overview of Relevant Literature 5

5.2 Study Rationale and Purpose 6

6 Study Objectives and Endpoints 8

6.1 Primary Objective 8

6.2 Secondary Objectives 9

6.3 Endpoints 9

7 Study Design 12

7.1 Study Design 12

7.2 Date Range for collected study data .... ***Error! Bookmark not defined.***

8 Study Population 12

8.1 Population of interest 12

8.2 Inclusion Criteria 12

8.3 Exclusion Criteria 12

9 Study Assessments and Procedures 13

9.1 Schedule of Activities 13

9.2 Detailed Study Assessments/Interventions 13

10 Data Collection and Management 18

10.1 Monitoring 18

10.2 Data Collection 18

10.3 Database Management and Quality Control 18

10.4 Statistical Considerations and Data Analysis 18

11 Ethical Considerations 19

11.1 Ethical Conduct of the Study ... 19
11.1.1 Declaration of Helsinki ... 19
11.1.2 Ethics Committee ... 19

11.2 Informed Consent ... 19

11.3 Study Data Protection ... 19

11.4 Subject Identification ... 20

12 Insurance ... 20

13 Reporting and Dissemination ... 20

14 Finance and Conflict of Interest Statement ... 20

15 Tables and Figures ... 21

16 References ... 22

1 Sponsor/Coordinating Investigator Information

Sponsor: UZ Brussel
Principal Investigator: Elisabeth De Waele
Subinvestigator(s): David Beckwée, Peter Janssens, Berenice Jimenez, Lynn Leemans
Coordinating Investigator: Sarah Bomans
Statistician: Wilfried Cools
Study site(s) and co-investigator(s): UZ Brussel

2 List of Abbreviations

ALA: 5-aminolevulinic acid hydrochloride
BIA: Bioelectrical Impedance Analysis
BRS: Brief Resilience Scale
COMET: Cellular Oxygen METabolism
COP: Cardiorespiratory Optimal Point
CAR: Cortisol Awakening Response
CCC: Concordance Correlation Coefficient
CFS: Chronic Fatigue Syndrome
CPET: Cardio-Pulmonary Exercise Test
DSQ-PEM: DePaul Symptom Questionnaire-Post Exertional Malaise
HR: Heart Rate
IC: Indirect Calorimetry
MitoPO_2: Mitochondrial Oxygen Tension
MitoPO2: Mitochondrial Oxygen Pressure
MitoVO2: Mitochondrial Oxygen Consumption
NCS: Neurocardiogenic Syncope
NIRS: Near-Infrared Spectroscopy
NRS: Numeric Rating Scale
ODR: Oxygen Disappearance Rate
OH: Orthostatic Hypotension
PA: Physical Activity
PEM: Post Exertional Malaise
PESE: Post Exertional Symptom Exacerbations
POTS: Postural Orthostatic Tachycardia Syndrome
PpIX-TSLT: Protophorphyrin IX triplet state lifetime technique
REE : Resting Energy Expenditure
RER: Respiratory Exchange Ratio
R-PFS: Revised-Piper Fatigue Scale
RR: Respiration Rate
SEM: Structural Equation Model
TEE: Total Energy Expenditure
TV: Tidal Volume
VE: Ventilation
WAYS: Ways of Coping Questionnaire
WHO: World Health Organization
6MIST: 6-Minute Incremental Step Test

3 Protocol Version History

Version N°	Version Date	Summary of changes
1	7/08/2024	
2	7/11/2024	Validation study becomes feasibility study

4 Trial Registration/Protocol Summary

**Information**	
Objectives:	1) To explore the feasibility of using the augmented two-day 6-minute incremental step test (6MIST) with wearable devices as a reliable and practical alternative to the two-day Cardio-Pulmonary Exercise Test (CPET) for assessing Post-Exertional Symptom Exacerbations (PESE) in long COVID patients.<br><br>2) To explore potential causal relationships with PESE by creating a Directed Acyclic Graph (DAG) based on a literature search and to test this DAG by creating a Structural Equation Model (SEM) based on measurements of nutritional parameters, body composition, mitochondrial dysfunction, psychological factors, autonomic dysfunction and physical activity.
Study population:	Adult long COVID patients with PESE
In- and exclusion criteria:	Inclusion criteria:<br><br>• ≥18 years old<br>• Long COVID patients following the World Health Organization (WHO) criteria: “the continuation or development of new symptoms 3 months after the initial SARS-CoV-2 infection, with these symptoms lasting for at least 2 months with no other explanation”<br>• Previously active as described in the WHO recommendations for physical activity (minimal 150 minutes/week of moderate intensity aerobic physical activity OR minimal 75 minutes of vigorous intensity activity/week OR an equivalent combination of moderate- and vigorous-intensity activity throughout the week)<br>• Patient suffers from PESE as defined by the DePaul Symptom Questionnaire PEM (Post-Exertional Malaise) subscale<br>• Able to understand and sign written informed consent in Dutch, French or English<br><br>Exclusion criteria:<br><br>• Any pre-existing conditions or new medical diagnosis that can alternatively explain the current symptoms<br>• Being unable to perform a cycle ergometer test as decided upon by the medical study team members<br>• Suffering from Chronic Obstructive Pulmonary Disease (COPD) GOLD classification category 2,3 or 4 (by the Global Initiative for Chronic Obstructive Lung Disease)

	• Allergies to medical adhesive bandages<br>• Skin conditions aggravated by sunlight including Porphyria<br>• Participation in other interventional trials<br>• Mitochondrial diseases<br>• Pregnancy<br>• Lactation
Endpoints:	• Feasibility of using the two-day 6MIST as an alternative for the two-day CPET<br>• Creation of a DAG to explore possible causal relationships with PESE (based on a literature search)<br>• Creation of a SEM to explore possible causal relationships with PESE (based on measurements)
Target sample size:	25
Statistical considerations:	• Objective 1: Evaluate the agreement of two methods through a Bland-Altman plot, a Concordance Correlation Coefficient (CCC) and using (Deming) regression.<br>• Objective 2: DAG's formally visualize the assumed causal structure.<br>• Objective 3: SEM can compare the assumed structure with the data and evaluate discrepancies between the two.

5 Background and Rationale

5.1 Overview of Relevant Literature

Definition of long COVID

Long COVID has been defined by the World Health Organization (WHO) as "the continuation or development of new symptoms 3 months after the initial SARS-CoV-2 infection, with these symptoms lasting for at least 2 months with no other explanation" [1].

Epidemiology and pathogenesis

According to the WHO, as of May 17, 2024, the SARS-Cov-2 virus has been responsible for 775 379 864 confirmed cases and almost 7 million deaths globally [2]. Many different clinical presentations were seen in the acute infection phase, going from asymptomatic to hospital admission to critical illness. As the pandemic became more chronic, more and more patients started suffering from post-infection symptoms, gathered under the name 'Long Covid'. The current estimated incidence of long COVID is approximately 144 million individuals globally [3]. It is thought that 10-30% with a recent history of SARS-Cov-2 infection develop Long Covid [4]. There is no clear link between the severity of the acute SARS-Cov-2 infection and the fact that subjects of all ages are affected, but the highest incidence is reported in patients aged 36-50 years [5]. The risk of developing long COVID does not differ significantly between vaccinated (minimum one dose) and non-vaccinated subjects [6].

Common symptoms of Long Covid may include dyspnea, severe and/or chronic fatigue, widespread muscle pain, cognitive impairments and sleep disturbances and may seriously affect quality of life [7].

An overview of the main Long Covid symptoms in descending order according to the WHO [8]:

Fatigue	78%
Dyspnea	78%
Cognitive impairment/brain fog	74%
**Post-Exertional Symptyom Exacerbations (PESE)**	67%
Muscle pain/spasms	64%
Cough	63%
Sleep disorders	62%
Tachycardia/Palpitations	60%
Altered smell/taste	57%
Headache	56%
Chest pain	55%
Joint pain	52%
Depression	50%

The underlying causes of long COVID are probably numerous and potentially overlapping. Multiple hypotheses for the pathogenesis of Post-Covid 19 have been put forward [5, 9]. The current existing hypotheses for the pathogenesis of long COVID are immune dysregulation, Vascular dysfunction & microclots, mitochondrial dysfunction, neurological dysfunction and cognitive dysfunction [5], [9] [10], [11], [12], [13].

The persistent nature of invalidating symptoms can significantly impact daily functioning and quality of life. Patients suffering from long COVID feel like they are being abandoned by healthcare workers and often receive conflicting information [14]. Current knowledge of pathophysiology and treatment options is limited, and more research is urgently needed.

PESE is marked by a significant worsening of fatigue and other symptoms after physical, mental, social, or emotional exertion that would normally not cause such a reaction in healthy people. This symptom flare-up can happen immediately after the exertion or be delayed by several hours to days. Episodes of PESE can persist for days or even weeks. The specific symptoms that worsen vary among individuals but often include severe fatigue, muscle or joint pain, cognitive issues, sleep disturbances, headaches, flu-like symptoms, and gastrointestinal problems [15].

5.2 Study Rationale and Purpose

1) <u>Feasibility of the two-day 6MIST as an alternative for the two-day CPET:</u>

The two-day CPET is currently the most reliable way to measure PESE. The 2-day CPET is recommended by the PESE working group of the National Institute of Neurological Disorders and Stroke (NINDS) [16]. The aim is to objectively demonstrate the loss of function these patients experience following exertion. The CPET methodology is a well-known standardized procedure for assessing physiologic responses to exercise and exertion. The two-day CPET approach has already proven to be able to assess function loss in studies with ME/CFS patients. Normally, the CPET is performed

only once and on a single day. However, a two-day CPET can assess function loss using two CPETs 24h apart. An amount of one-day CPET studies reported a lack of significant differences between ME/CFE patients and controls, but significant differences were observed in the second test of the 2-day CPET [17]. The two-day CPET is a demanding test for long COVID patients, physically and psychologically. Even the trajectory to and from the hospital can already be exhausting for them. The augmented two-day 6MIST is an enhanced bedside test with wearable wireless sensor technology that is easy to perform and can be applied at home [18]. This test would be an excellent alternative to the two-day CPET to assess PESE, since it is much less physically demanding for the fragile long covid patients and much more practical and easier to use in clinical trials since it can be done at the patient's home. Therefore, there is a clear need and rationale to test the feasibility of the augmented two-day 6MIST as an alternative to the two-day CPET.

2) Identifying causal factors of PESE

Among the numerous descriptive studies documenting fatigue and other symptoms associated with Long COVID, only a small fraction specifically measures PESE. The underlying pathophysiology of PESE is not yet understood. Research indicates that PESE may involve multiple systems in the body, including the mitochondrial and metabolic systems [19]. However, the precise mechanisms remain unclear, and more research is needed to uncover the exact biological and psychological processes that cause PESE. This lack of definitive understanding makes it challenging to develop targeted treatments and management strategies for those affected [19]. By identifying the potential underlying mechanisms of PESE, further research can focus on the development of targeted treatments that reduce or prevent PESE episodes, improving the quality of life for long COVID patients. It can also help to draw up guidelines for safe levels of physical, mental, and emotional activity for patients, helping to avoid symptom exacerbations. This is why we want to investigate the possible underlying causal relationships between nutritional parameters & body composition (metabolism), mitochondrial dysfunction, psychological factors, physical activity, autonomic dysfunction and PESE based on a DAG and a SEM. We will conduct a thorough literature search to create a DAG. To check if the potential causal relationships in the DAG are correct, we will conduct a SEM. To create the SEM, we will measure nutritional parameters, body composition, mitochondrial dysfunction, psychological factors, autonomic dysfunction and physical activity. These measurements are easy to perform and are not burdensome for the patient. The SEM is purely explorative and will be interpreted with caution since our sample size is too small to draw any firm conclusions.

Nutritional parameters as causal factor for PESE
A lot of long COVID patients show signs of malnutrition [20]. Malnutrition leads to insufficient energy intake, affecting overall energy levels and thereby potentially leading to fatigue and PESE [21]. Indeed, when someone with malnutrition engages in physical or even mental activity, their body may not have enough energy reserves to recover. Especially when malnutrition may prevent the patient's body to replenish energy stores after exertion [21], PESE could occur. Our own clinical experience with long COVID patients at UZ Brussel showed that 50% of long COVID patients do not meet their energy requirements and that they have an imbalanced macronutrient intake [20]. Therefore, keeping track of a food diary and performing an Indirect Calorimetry (IC) and Bioelectrical Impedance Analysis (BIA) measurement is necessary. By assessing a food diary, we track what participants eat and analyze their diet's macro- and micronutrient composition. Food diaries help in identifying eating habits, meal frequency, portion sizes, and food choices, which can be affected by long COVID symptoms such as fatigue, loss of taste, or gastrointestinal issues.

Body composition as causal factor for PESE
Appelman et al. (2024) [19] showed that severe exercise-induced muscle damage and subsequent regeneration are associated with the pathophysiology of PESE in long COVID, therefore there is a clear rationale to investigate the link between muscle wasting, weakness and damage caused by malnutrition and PESE.

Mitochondrial dysfunction as a potential cause for PESE
A pathogenic mechanism in long COVID **possibly contributing to PESE** is mitochondrial dysfunction [22, 23]. Mitochondria are responsible for generating Adenosine Triphosphate (ATP), dysfunction in the organelles could lead to impaired ATP production and thus a lack of energy availability. After exertion, the body possibly has difficulty replenishing its energy stores, leading to PESE [24]. Mitochondrial dysfunction also causes elevated oxidative stress through excessive production of mitochondrial reactive oxygen species (mtROS), contributing to cell dysmetabolism, persistent inflammation and immune dysregulation. This might contribute to fatigue, muscle pain and other PESE symptoms [25].

Psychological factors as a potential cause for PESE
PESE might be influenced by multiple psychosocial factors such as stress, anxiety, negative mood and bad coping strategies [26]. A person's ability to manage stressful events or emotions can **possibly trigger symptoms of PESE** by modulating the attenuated Cortisol Awakening Response (CAR) [26]. High psychological stress and negative feelings can lead to hypocortisolism, which results in poorer utilization of cortisol. This can lead to poorer recovery after some type of high exertion through inhibitions in mitochondrial respiration [27]. In breast cancer, there is evidence that worry significantly contributes to neurocognitive dysfunction, even before treatments like chemotherapy. This suggests cognitive impairments may arise from psychological stress related to a cancer diagnosis and not just the treatment itself. Understanding how worry and similar psychological factors influence PESE could lead to more effective interventions that reduce cognitive and physical symptoms in long COVID [28].

Autonomic dysfunction as a potential cause for PESE
Orthostatic intolerance and autonomic dysfunction have been recognized as a unique symptom cluster associated with Long COVID [29]. Autonomic dysfunction refers to any disruption in the autonomic nervous system, including postural orthostatic tachycardia syndrome (POTS). POTS has been increasingly reported in patients after COVID-19 infection [30]. Other, less common forms of autonomic dysfunction seen in Long COVID patients include neurocardiogenic syncope (NCS) and orthostatic hypotension (OH) [31]. Some studies suggest that POTS may play a role in the pathophysiology of Long COVID, potentially explaining persistent symptoms like fatigue and cognitive issues in these patients. However, evidence supporting this hypothesis is still missing [30]. The mechanisms of action may involve direct tissue damage, immune dysregulation, hormonal imbalances, elevated cytokine levels, and ongoing low-grade inflammation [32].
Physical activity (PA) as a potential cause for PESE
There is a clear connection between lack of physical activity and an increase in feelings of fatigue and decreased energy levels. Evidence suggests that the link between physical inactivity and fatigue is more significant than the impact of other factors like body mass index and chronic illness on energy and fatigue levels [33]. Besides that, patients suffering from PESE are more likely to avoid PA since this is also often a trigger for their symptom exacerbations. Evidence suggests that repeated exercise on consecutive days in patients with ME/CFS leads to altered muscle metabolism and abnormal autonomic nervous system responses [34]. Therefore, we will measure PA with an activity tracker to see if there is a link with PESE.

6 Study Objectives and Endpoints

6.1 Objective 1

To explore the feasibility of the two-day augmented 6-minute incremental step test with wearable devices as a reliable and practical alternative to the two-day CPET for assessing PESE in long COVID patients.

6.2 Objective 2

To explore potential causal relationships with PESE by creating a DAG based on a literature search and to test this DAG by creating a SEM based on measurements of nutritional parameters, body composition, mitochondrial dysfunction, psychological factors, autonomic dysfunction and physical activity.

6.3 Endpoints

Primary endpoints

*Endpoints related to **objective 1**:*

- *Objective endpoints:*

The difference in oxygen uptake at peak (VO2 in ml/min/kg) VO2 between day 1 and day 2

- *Subjective endpoints:*

The difference in Rate of Perceived Exertion (RPE) between day 1 and day 2. It will be measured at the following time points:

Day 1:
15 minutes (+- 15 min) before the test (CPET or 6MIST)
15 minutes (+- 15 min) after the test

Day 2:
24h (+- 120 min) after test 1 and before test 2

*Endpoints related to **objective 2**:*

Nutritional parameters and body composition:

IC:

- Resting Energy Expenditure (REE) in kcal/kg/day
- Total Energy Expenditure (TEE) in kcal/kg/day

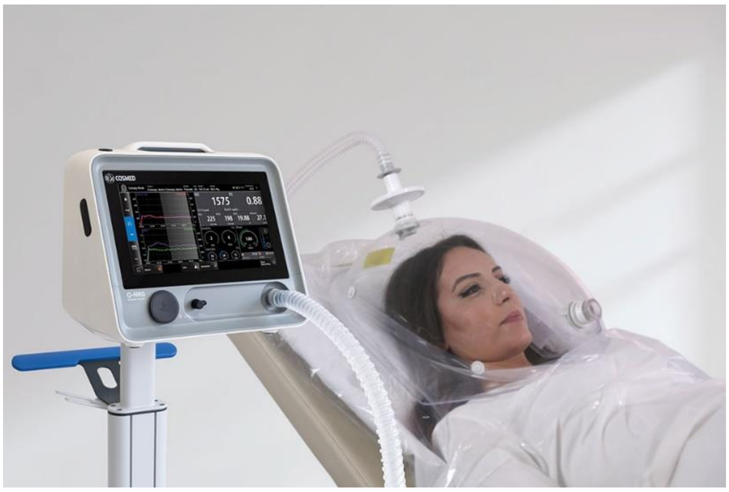

Figure 1: Indirect calorimetry

BIA:

- Phase angle (degrees)
- Muscle mass (kg/m^2)
- Fat mass index (kg/m^2)
- Fat-free mass index (kg/m^2)
- Fat to fat-free mass ratio
- Hydratation (%)

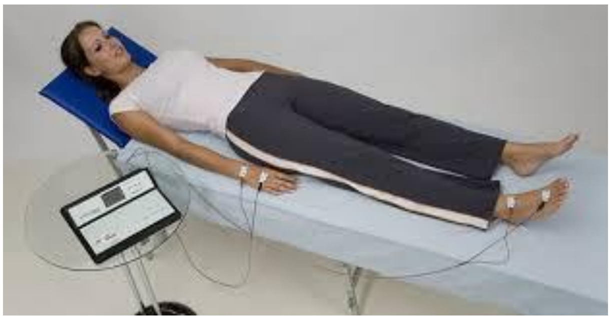

Figure 2: BIA measurement

Food diaries:

- Adequacy of feeding: the ratio between intake of calories and proteins and the individual need
- Mean daily intake: calories, fats, proteins, carbohydrates

Mitochondrial respiration with Cellular Oxygen METabolism (COMET):

- Oxygen availability as the partial pressure of oxygen (mitoPO_2) (mmHg)

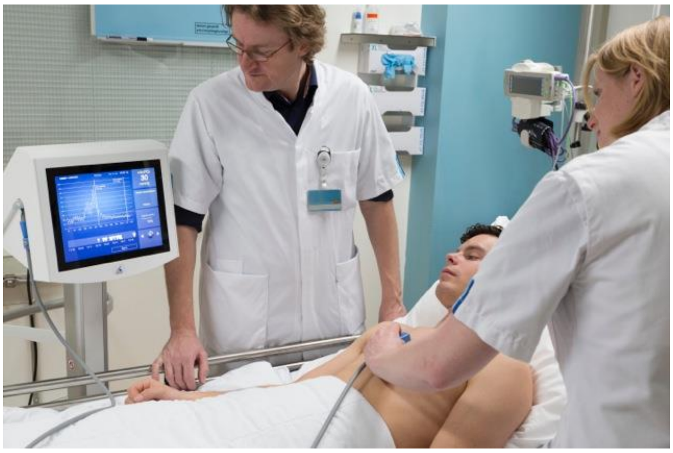

Figure 3: COMET measurement

Psychological factors:

- Kinesiophobia with the Tampa Scale for Kinesiophobia (TSK)
- Ability to bounce back after stressful events with the Brief Resilience Scale (BRS)
- Coping style with the WAYS (Ways of Coping Questionnaire)

Autonomic dysfunction:

- Autonomic symptoms measured with the COMPASS-31 questionnaire

Physical activity (PA):

- Level of PA and sedentary behavior assessed by accelerometry (activity tracker)

<u>Secondary endpoints</u>
The secondary endpoints are only related to **objective 1.**

Objective endpoints during CPET and 6MIST:

Performance measures during CPET:
- Oxygen uptake at first ventilatory threshold (VO2 in ml/min/kg)
- Oxygen uptake at peak (VO2 in ml/min/kg)
- Workload (Watts) at first ventilatory threshold
- Workload (Watts) at peak
- Heart Rate (HR) peak
- Ventilation (VE) (Tidal Volume (TV) and Respiration Rate (RR))
- VE/VO2 slope
- VO2/HR slope (= O2 pulse)
- Respiratory Exchange Ratio (RER) = VCO2/VO2
- Cardiorespiratory Optimal Point (COP) = VO2/VE

Performance measures during 6MIST:
- Oxygen uptake at peak (VO2 in ml/min/kg) VO2peak (30sec rolling average)
- Oxygen uptake at peak (VO2 in ml/min/kg) VO2 peak (5 sec rolling average)
- VE/VO2 slope
- VO2/HR slope (O2 pulse)
- HR peak
- VE (TV and RR)
- COP

Subjective endpoints during CPET and 6MIST, and 7 days after each experimental session:

CPET and 6MIST:

- Overall fatigue and mental fatigue/brain fog with the Revised Piper Fatigue Scale (R-PFS)
- Self-reported neuromuscular complaints intensity with a 10-point Likert scale

The secondary subjective endpoints were chosen in line with the most frequently reported symptoms of PESE and will be measured at the same time points as the RPE and 7 days (± 1 day) after each experimental session.

<u>Explanatory endpoints:</u>

- Vaccination status
- Demographics: date of birth/age, ethnicity, BMI, hospitalization for COVID-19, educational level, employment status, native language

7 Study Design

7.1 Study Design

This clinical trial will be a randomized cross-over study, meaning data will be collected from participants at two different points in time to compare the results of the two-day 6MIST to the results of the two-day CPET. In a feasibility study, it is common practice to conduct the two tests on the same day to ensure standardization and consistency. This approach is problematic for our study involving a two-day CPET and a two-day 6MIST in a long COVID population. As mentioned before, these patients suffer from PESE, so performing both maximal tests on two consecutive days could negatively impact their performance and health. Therefore, we opted for a randomized cross-over design to perform one test (two-day CPET or two-day 6MIST) and then conduct the other test one month (+- 10 days) later. This spacing helps mitigate the physical strain and potential interference between tests. It also would help reduce the participant dropouts due to the demanding nature of the study.

We will conduct the DePaul Symptom Questionnaire-Post Exertional Malaise (DSQ-PEM) during the initial screening to see if patients are eligible for the study. To minimize patient burden and to reduce the need for multiple hospital visits, we will also perform the COMET, BIA, IC, lung function test, and questionnaires on the same day. While same-day testing is preferred for consistency, the COMET, BIA, IC, questionnaires and lung function test may be conducted on separate days if scheduling conflicts, or unforeseen circumstances arise. Study activities will only take place after signing informed consent.

8 Study Population

8.1 Population of interest

Long COVID patients (only adults) who were frequently active according to the World Health Organization (WHO) criteria before their COVID infection.

8.2 Inclusion Criteria

- ≥18 years old
- Long COVID patients following the WHO criteria: “the continuation or development of new symptoms 3 months after the initial SARS-CoV-2 infection, with these symptoms lasting for at least 2 months with no other explanation”
- Previously active as described in the WHO recommendations for physical activity (minimal 150 minutes/week of moderate intensity aerobic physical activity OR minimal 75 minutes of vigorous intensity activity/week OR an equivalent combination of moderate- and vigorous-intensity activity throughout the week)
- Patient suffers from PESE assessed by the DePaul Symptom Questionnaire PEM (Post-Exertional Malaise) subscale
- Able to understand and sign written informed consent in Dutch, French or English

8.3 Exclusion Criteria

- Having pre-existing conditions or new medical diagnosis that can explain their symptoms
- Being unable to perform a cycle ergometer test as decided upon by the medical study team members
- Suffering from Chronic Obstructive Pulmonary Disease (COPD) GOLD classification category 2,3 or 4 (by the Global Initiative for Chronic Obstructive Lung Disease)

- Allergies to medical adhesive bandages
- Skin conditions aggravated by sunlight, including Porphyria
- Mitochondrial diseases
- Participation in other interventional trials
- Pregnancy
- Lactation

9 Study Assessments and Procedures

9.1 Schedule of Activities

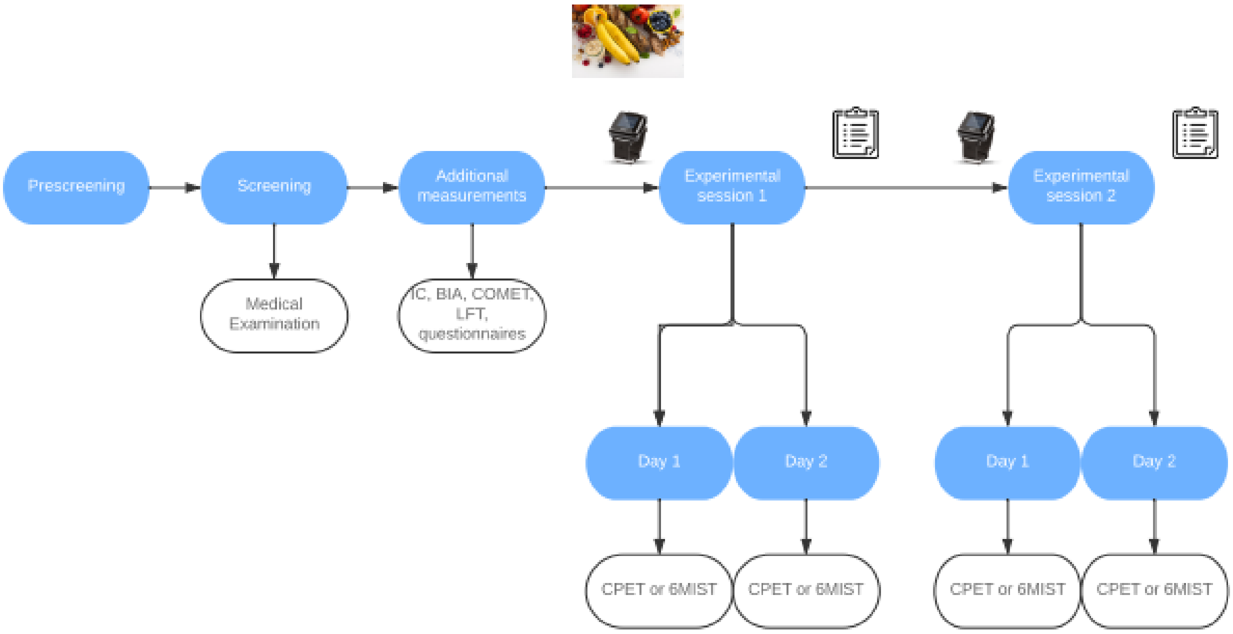

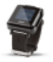 = Accelerometry (during 7 days before each experimental session)

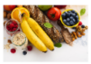 = Food diaries (to be completed before experimental session 1)

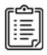 = Revised Piper Fatigue Scale, 10-point Likert scale (7 days after each experimental session)

9.2 Detailed Study Assessments/Interventions

Recruitment:
Leaflets and posters will be spread throughout UZ Brussel and the Health Campus of the VUB (VUB, Erasmus Hogeschool, and UZ Brussel). The academic centers of general practice of the VUB, ULB and UCL will be invited to share information about the study and motivate their members to participate in the recruitment of potential participants. Another way to reach the general practitioners is through General Practitioner (GP) magazines or through GP groups (for example Domus Medica). Participants will also be recruited through the members of the Advisory Board. Mrs. Ann Li, Advisory Board member and chairwoman of *Post-Covid Gemeenschap* (the Flemish patients' association for people with long COVID) agreed to inform their patient-members about the study through social media and newsletters. Additionally, advertisements and announcements in newspapers,

online newsletters, mentions in webinars or on television, paramedical professional associations (for example AXXON) and publications from participant support groups can also be used. General practitioners and pharmacies working within a 15-minute car travel distance from UZ Brussel can be contacted and invited to recruit participants. If necessary, also physiotherapy practices can be contacted. If they agree, they will receive recruitment leaflets.

Prescreening:
Long COVID patients interested in participating in the study can reach out to the research team for further information about the study. When a potential candidate reaches out to the research team, a pre-screening will take place by telephone. Inclusion and exclusion criteria will be evaluated through an anamnesis with standardized questions. The topics discussed during the prescreening will be the language, the age, the SARS-Cov-2 infection, their previous physical activity state, PESE, persisting symptoms, allergies and their social security system. The survey can be found as an attachment. If they seem eligible for the study based on the prescreening, an appointment for a screening on site with a medical doctor will be scheduled as soon as possible. After the prescreening, the informed consent document will be sent to the participant so they can read it carefully and sign it before or during the screening. No data will be collected from the prescreening.

Screening:
Before any study activity takes place, the signed informed consent document needs to be returned to the research team. Subjects will give informed consent themselves at the time of this screening consultation or by returning the signed documents before the screening consultation. The screening will take place on site. To be able to participate, female premenopausal women will be asked to take a urine pregnancy test. A medical doctor will conduct a medical examination. The medical doctor will check the patient's medical history and medical file. Demographics (age, gender, ethnicity, educational level, employment status, smoking, alcohol, leisure time and sports) and prior and concomitant medication will be registered. In case of underlying pre-existing comorbidities with physical and/or psychological symptoms, eligibility might be negative. If the patient does not have a medical file at UZ Brussel, a new medical file will be made and we will access their medical history through E-health (the participant gives his permission to do so by signing the informed consent). He/she will have the opportunity to ask the research team supplementary questions.

The medical examination will consist of ruling out contra-indications for the CPET, measuring height, weight, blood pressure, saturation, resting heart rate (vital signs) and a conclusion. The medical examination is necessary to check if the patient is considered able to perform a cycle ergometer test. In case of an absolute contra-indication: no CPET. In case of a relative contra-indication: CPET depends on judgement of a medical doctor and only with direct supervision of a medical doctor [35]. If considered eligible for the study by the medical doctor, we set a date with the patient for the experimental sessions. The intervention will take place as soon as possible.

The bodyweight and the length of the study participants will be measured using a calibrated (on fixed intervals by biotechnical department) scale and measuring rod.

Table 1: Contra-indications CPET [35]

Absolute	Relative
Recent significant change in the resting ECG suggesting significant ischemia, recent myocardial infarction (within 2 days) or other acute cardiac events	Severe arterial hypertension (I.e. systolic blood pressure of >200 mmHg and/or diastolic blood pressure of >110 mmHg) at rest
Unstable angina pectoris	Moderate stenotic valvular heart disease
Uncontrolled cardiac dysrhythmias causing symptoms or hemodynamic compromise	Uncontrolled medical conditions like electrolyte abnormalities (e.g. hypokalemia)
Symptomatic severe aortic stenosis	Left main coronary artery stenosis or its equivalent
Uncontrolled heart failure	Tachydysrhythmia or bradydysrhythmia
Acute pulmonary embolus, pulmonary infarction, pulmonary edema or deep venous thrombosis	Hypertrophic cardiomyopathy and other forms of outflow tract obstruction
Acute myocarditis or pericarditis	Neuromotor, musculoskeletal or rheumatoid disorders that are exacerbated by exercise
Suspected or known dissecting aneurysm	High-degree atrio-ventricular block
Acute systemic infection, accompanied by fever, body aches or swollen lymph glands	Ventricular aneurysm
Active endocarditis	Uncontrolled metabolic disease
Respiratory failure	Chronic infectious disease
Uncontrolled asthma	Mental or physical impairment leading to inability to exercise adequately
	Recent stroke or transient ischemic attack

Experimental sessions:

The day of screening

The DSQ-PEM questionnaire will be taken once at the time of screening to evaluate the PESE inclusion criteria.

After the screening, the research team will perform the IC and BIA measurements. We choose to do this on the day of screening so the patients can come in fasting conditions, which is required for these measurements. According to standard procedures, the study team will perform the BIA measurement with the Akern Nutrilab® (Akern, Italy). The IC will be assessed with Qnergy+® (Cosmed, Italy) by the study team in accordance with the ICALIC study group recommendations [36]. The participants must be at rest and in supine position, without supplementary oxygen until a stable measurement is obtained. A stable measurement means less than 10% variability allowed in VCO2 and VO2.

BIA is a non-invasive method for assessing body composition, used in daily clinical practice. BIA provides estimates of body fat, lean body mass, and total body water, which are important metrics for understanding the physical impact of long COVID. It also measures the phase angle, which is a measure of cell and, thereby, body vitality. IC measures oxygen consumption and carbon dioxide production, which corresponds to cellular respiration. This makes it possible to calculate the resting energy expenditure (REE) in kcal/kg/day. REE is defined as the sum of basal energy expenditure and diet-induced thermogenesis [36]. Calculating the REE is possible because oxygen consumption and carbon dioxide production are highly correlated with heat production [36]. This technology is non-invasive and was used more than 500 times in 2023 at UZ Brussel, mostly in the clinical setting.

To diminish the burden on the patients on the days of the experimental sessions, the research team will also perform the mitochondrial function assessment (COMET) on the screening day. Measuring mitochondrial function in humans is challenging and a gold standard assessment is currently lacking. COMET monitoring is a promising, fast, and non-invasive methodology to assess mitochondrial function through the transdermal assessment of oxygen consumption [37]. It calculates the tissue oxygen consumption rate on the skin and consequently does not need blood sampling. Mitochondrial oxygen consumption rates could eventually serve as a biomarker for disease severity and treatment response, helping to personalize and optimize therapeutic strategies. COMET uses the protophorphyrin IX triplet state lifetime technique (PpIX-TSLT), which measures mitochondrial oxygen pressure (mitoPO2) by measuring the oxygen-dependent optical properties of protophorphyrin IX (PpIX) [38]. PpIX is an endogenous porphyrin produced in the mitochondria from the TCA cycle intermediate, succinyl-Coa. After photoexcitation, PpIX produces a delayed fluorescent signal that is effectively quenched by oxygen, meaning that the lifetime is inversely related to the amount of oxygen in the tissue. Application of the PpIX precursor 5-aminolevulinic acid hydrochloride (ALA) to cells enhances the PpIX concentration, and the delayed fluorescent signal. Topical application of 2.5% ALA cream to the skin allows for the use of this technique on the skin [39]. With this technique, it is possible to measure the mitochondrial oxygen tension (mitoPO$_2$) and combine it with a measurement of the oxygen disappearance rate (ODR). Microvascular blood flow cessation (by applying pressure on the measuring probe) makes it possible to calculate the tissue oxygen consumption rate [40]. The technology is EC certified and validated in clinical studies and had not been used before in long COVID patients [41].

A psychologist will conduct and interpret three psychological questionnaires TSK, BRS and WAYS to measure kinesiophobia, resilience and coping respectively. All these questionnaires are validated in different patient populations and will be taken and interpreted by a qualified psychologist. The COMPASS-31 questionnaire will be filled in by the patients and measures autonomic symptoms and dysfunction.

A lung function test (including a pulmonary diffusion testing) will be performed to exclude underlying respiratory conditions that could affect exercise performance and to ensure the safe conduct of the CPET.

<u>Between the screening and experimental session 1</u>
Participants will complete a standardized food diary for 3 days. They will note every nutritional intake (food and drinks) they consume daily for 3 consecutive days. We request 2 working days and one free day for a representative idea of the mean habitual intake. The nutrition is calculated using Nubel pro®. Feeding adequacy will be calculated as the ratio between TEE and nutritional intake.

<u>Experimental sessions</u>
Every patient will participate in two experimental sessions at the hospital one month apart (a single experimental session consists of two consecutive days) during which the following measurements will be performed on each day 24h (± 2h) apart: a CPET or a 6MIST. To rule out test order effects, patients will be randomized, and chance will decide whether they start with the CPET or the 6MIST.

Spirometry will be performed prior to the CPET as part of standard care. It is used to predict the Maximum Voluntary Ventilation (MVV) from the Forced Expiratory Volume in one second (FEV1), which is crucial for interpreting CPET results. The CPET will be performed in a standardized, validated way at the University Hospital Brussels and under the supervision of trained medical personnel. The CPET methodology is standardized as a well-accepted procedure to assess physiological responses to exertion in many illness conditions [17]. The protocol is patient-tailored to obtain a fatigue-limited exercise duration of 8 to 12 minutes. The predicted peak wattage will be calculated according to the formula work rate peak = -102 + (1,5 x weight (kg)) + (1,9 x height (cm)) – (2 x age) – (sex x 60 [male = 0, female = 1]) and considering the overall clinical profile of the patient. The calculated predicted peak wattage is divided by 10 to obtain the work rate increment protocol [42]. The test will be performed on a bicycle. Before starting the test, patients will be connected to

an electrocardiogram (ECG), oximeter and blood pressure cuff. A mouthpiece will be used to collect information about gas exchange (oxygen and carbon dioxide). Before the actual start of the test, resting data will be collected for 3 minutes. Patients start at 0 watts per minute. The intensity will be increased until exhaustion (incremental step protocol) or if the test needs to be stopped for safety reasons. Exhaustion will be deemed to have occurred when the participant can no longer maintain the required power output or wishes to stop. The reason for stopping the exercise test will be recorded. Participants will be instructed to maintain a pedal frequency of 60-70 rotations per minute receiving feedback when being out of this scope. The 12-lead ECG will be recorded continuously throughout the test, blood pressure will be measured non-invasively and oxygen saturation will be monitored continuously. Before starting the test and at peak exercise, the Borg RPE scale [43] will be used for the self-assessment of subjective exertion [from 6 (= no exertion at all) to 20 (= maximum exertion)] and dyspnea [from 6 (= no breathing difficulty) to 20 (= maximum breathing difficulty)] and helps to estimate how hard the test is at that moment for the patient. During the CPET, patients will wear a Train-Red Fyer Near-Infrared Spectroscopy (NIRS) Muscle Oxygen Sensor to measure muscle tissue oxygenation.

The augmented 6MIST consists of incremental pace stationary stepping (increasing pace by 5 steps every 30 seconds using a metronome) for a maximum of 6 minutes while clinical cardio-respiratory variables are simultaneously recorded. A portable metabolic analyzer, VO2 Master Pro (VO2 Master Health Sensors Inc., British Columbia, Canada) will be used to assess VO2 and VE (TV and RR). The Polar Sense of Polar 10 heart rate monitor will be used to measure HR continuously. The Train-Red Fyer NIRS Muscle Oxygen Sensor will be used to collect the muscle tissue oxygenation. The sensor (VO2 Master), Polar Sense of Polar 10 and Train-Red NIRS Oxygen Sensor will simultaneously be connected to the SplendoMonitor (Apple iPad) App (SplendoHealth, USA) to collect all VO2 kinetics and data continuously.

All the patients will complete the two-day CPET and two-day 6MIST with a month (±10 days) between the tests. Patients will be asked to refrain from strenuous exercise and their known (avoidable) PESE triggers 48h before the start of the experimental sessions.

Seven days after each experimental session, the R-PFS and 10-point Likert scale will be sent to the patient via REDCap to ask about their PESE.

We will measure PA with accelerometry to measure if there is a potential causal relationship between PA and PESE. All patients will carry an activity tracker during 7 days before the first experimental session and during 7 days before the second experimental session, to measure their daily physical activity and sedentary behavior.

The results of the IC, BIA, food diaries, COMET, TSK, BRS, WAYS, COMPASS-31 and activity tracker will be included in the SEM.

Feedback consultation:
All participants will be invited for a last visit at the UZ Brussel for a consultation with the nutritionist, psychologist and physiotherapist and/or medical doctor (wrap-up and farewell). They will receive a folder with their individual results concerning nutrition, psychological questionnaires and physical performance on the CPET and 6MIST. This consultation is completely without obligation for the study participants.

10 Data Collection and Management

10.1 Monitoring

The investigator must make all trial documentation and related records available in case a monitoring visit or audit by the Sponsor is requested. Also, in case of regulatory inspections all trial documentation should be made available to the inspector(s). All participant data must be handled and treated confidentially.
The Sponsor's monitoring frequency will be determined prior to the start of the trial. A monitoring plan will be generated detailing the frequency and scope of monitoring for the trial. Throughout the course of the trial the monitoring plan can be adjusted as necessary. Monitoring of the UZ Brussel study site will be performed by the monitor of the Clinical Trial Center.

10.2 Data Collection

An Electronic Data Capture system "***REDCap***" will be used for data collection. The system is validated and access to all levels will be granted/revoked by the Sponsor representative. Trial data should be entered within reasonable time after the subject attended the visit. Corrections/modifications will be automatically tracked by an audit trail detailing date and time of the correction and the name of person performing the correction.

10.3 Database Management and Quality Control

The sponsor and the investigator shall keep a clinical trial master file. The clinical trial master file shall at all times contain the essential documents relating to the clinical trial which allow verification of the conduct of a clinical trial and the quality of the data generated.

The sponsor and the investigator shall archive the content of the clinical trial master file for at least 25 years after the end of the clinical trial, unless other EU law requires archiving for a longer period. The medical files of subjects shall be archived in accordance with national law.

The content of the clinical trial master file shall be archived in a way that ensures that it is readily available and accessible, upon request.

10.4 Statistical Considerations and Data Analysis

Objective 1:

- Deming regression
  A Deming regression between the 6MIST and CPET accommodates the measurement error in both measurements. The model estimates will reveal how one can be transformed into the other.

- Bland-Altman plot
  To evaluate the agreement between the 6MIST and CPET, a Bland-Altman plot will be constructed. This plot will visually represent the differences between the two tests against their mean values, helping to identify systematic differences between the scales.

- CCC:
  CCC will be utilized to evaluate the agreement between the two testing methods. The CCC will measure both the precision and accuracy of the 6MIST relative to the CPET, providing a comprehensive assessment of its validity as an alternative testing method.

Data will be cleaned and checked before analysis. Statistical analyses will be performed using SPSS. The significance level for all tests will be set at $\alpha = 0.05$.

Objective 2:

- DAG:
  Literature Review and DAG development: A comprehensive review of relevant literature will be conducted to identify and hypothesize potential causal relationships between the variables of interest. The DAG will be constructed based on this review to visually represent the hypothesized causal pathways. The DAG will illustrate the hypothesized relationships among the variables, including direct and indirect effects on PESE. The DAG will serve as a theoretical framework for subsequent statistical testing.

- SEM:
  A SEM will be developed based on the DAG. The model will specify the relationships among the variables and the pathways through which these variables influence PESE. Modifications to the causal structure may be suggested by this type of analysis, comparing the assumed model with its alternatives, albeit only very tentatively because of the limited sample size.

Sample size:
A sample size could not be calculated since we are not looking for a difference but an agreement. As no pilot study has yet taken place, we do not have any measurements to base our sample size on. This is why we did a literature search to look for similar agreement studies. We based our sample size on the study of Molinger et al. (2024) [44], and took into account potential attrition. Based on our previous experience at UZ Brussel with long COVID patients in the UNLOCK study and based on the fact that the two-day CPET and two-day 6MIST are physically demanding for these patients, we have incorporated an anticipated a dropout rate of 40% into our sample size. This is why we opt for a sample size of 25 participants.

11 Ethical Considerations

11.1 Ethical Conduct of the Study

11.1.1 Declaration of Helsinki

The trial will be performed in accordance with the Declaration of Helsinki, the conditions and principles of Good Clinical Practice, the protocol and applicable local regulatory requirements and laws.

11.1.2 Ethics Committee

Before the start of the trial or implementation of any amendment, approval of the trial protocol and amendments, informed consent forms and other relevant documents will be obtained from the applicable ethical committee(s).

11.2 Informed Consent

Each participant shall provide Informed Consent before performance of any study-related activities. The IC form that is/are used must be approved by reviewing EC and be in a language that the participant can read and understand. The ICF should be in accordance with current ICH and GCP guidelines and with applicable local regulations.

11.3 Study Data Protection

The collection and processing of personal data from participants enrolled in the study will be limited to those data that are necessary to fulfill the objectives of this study. These data must be collected and processed with adequate precautions to ensure confidentiality and compliance with applicable data protection laws and regulations.

11.4 Subject Identification

The participant identification will be treated as confidential and will be filed by the investigator in an identification log. This log is kept at the participating site and shall not be copied. In all reports and communication between the site and the Sponsor the participant shall be identified with a participant study number.

12 Insurance

UZ Brussel/VUB is, as Sponsor of the trial, responsible for ensuring appropriate general/product liability insurance and as required in accordance with applicable laws and regulations, country-specific liability insurance coverage for claims made by a trial subjects for injury arising from the subject's participation in the trial.

13 Reporting and Dissemination

The data and information collected during this trial will be reported in a publication in a scientific/medical journal. Reporting of trial results will be performed according to local regulations.

Data collected within the UZ Brussel or VUB as an employee or (PhD) student of the VUB are owned by the UZ Brussel VUB. For the correct authorship rules we refer to the International Committee of Medical Journal Editors:
https://www.icmje.org/recommendations/browse/roles-and-responsibilities/defining-the-role-of-authors-and-contributors.html

14 Finance and Conflict of Interest Statement

Investigators and study team members will provide the Sponsor with sufficient, accurate financial information in accordance with local regulations to allow the Sponsor to submit complete and accurate financial certification or disclosure statements to the appropriate regulatory authorities/ethics committee. Any update of information on financial interests should be disclosed during the course of the study.

To cover their transportation costs to the hospital as well as the extended time spent during the study visits, the participants will receive a payment of € 50 per experimental session in the form of a gift card (except for the initial prescreening consultation with anamnesis and informed consent). Every participant who needs to use the hospital parking lot can get a free parking ticket. All consultations, related to the study, will be free of charge to the patients. Any other contact/consultation with the PMR department or the Nutrition department will fall out of the scope of the study.

15 Tables and Figures

Table 1: Overview of study assessments

	Pre-screening	Screening	Between screening and experimental session 1	Experimental session 1	Experimental session 2	Seven days after each experimental session	One week before each experimental session
Anamnesis (inclusion/exclusion)	X						
Informed consent		X					
Demographics		X					
Prior and concomitant medication		X					
Medical history		X					
Pregnancy test*		X					
Vaccine doses + type of vaccine		X					
Height		X					
Weight		X					
Vital Signs		X					
Medical exam (ruling out contra-indications for CPET)		X					
DSM-PEM		X					
IC		X					
BIA		X					
COMET		X					
LFT		X					
TSK		X					
WAYS		X					
BRS		X					
R-PFS				X	X	X	
10-point Likert scale				X	X	X	
COMPASS-31		X					
Food diaries			X				
Spirometry + Two-day CPET				X	X		
Two-day 6MIST				X	X		
Accelerometry							X

*Only in premenopausal women (urine test)

16 References

1. Organization, W.H. *Post COVID-19 condition (Long COVID)*. 2022 7 december 2022; Available from: https://www.who.int/europe/news-room/fact-sheets/item/post-covid-19-condition.
2. Organization, W.H. *Number of COVID-19 cases reported to WHO (cumulative total)*. 2024; Available from: https://data.who.int/dashboards/covid19/cases?n=c.
3. Hanson, S.W., et al., *A global systematic analysis of the occurrence, severity, and recovery pattern of long COVID in 2020 and 2021.* medRxiv, 2022.
4. Parums, D.V., *Editorial: Long COVID, or Post-COVID Syndrome, and the Global Impact on Health Care.* Med Sci Monit, 2021. **27**: p. e933446.
5. Davis, H.E., et al., *Long COVID: major findings, mechanisms and recommendations.* Nat Rev Microbiol, 2023. **21**(3): p. 133-146.
6. Taquet, M., Q. Dercon, and P.J. Harrison, *Six-month sequelae of post-vaccination SARS-CoV-2 infection: A retrospective cohort study of 10,024 breakthrough infections.* Brain Behav Immun, 2022. **103**: p. 154-162.
7. Batiha, G.E., et al., *Pathophysiology of Post-COVID syndromes: a new perspective.* Virol J, 2022. **19**(1): p. 158.
8. Lippi, G., F. Sanchis-Gomar, and B.M. Henry, *COVID-19 and its long-term sequelae: what do we know in 2023?* Pol Arch Intern Med, 2023. **133**(4).
9. Astin, R., et al., *Long COVID: mechanisms, risk factors and recovery.* Exp Physiol, 2023. **108**(1): p. 12-27.
10. Peluso, M.J., et al., *Impact of Pre-Existing Chronic Viral Infection and Reactivation on the Development of Long COVID.* medRxiv, 2022.
11. Gaebler, C., et al., *Evolution of antibody immunity to SARS-CoV-2.* Nature, 2021. **591**(7851): p. 639-644.
12. Serena, S., *Nervous System Consequences of COVID-19.* Science, 2022. **375**(6578).
13. Dotan, A., et al., *The autonomic aspects of the post-COVID19 syndrome.* Autoimmun Rev, 2022. **21**(5): p. 103071.
14. Yong, S.J., *Long COVID or post-COVID-19 syndrome: putative pathophysiology, risk factors, and treatments.* Infect Dis (Lond), 2021. **53**(10): p. 737-754.

15. Vernon, S.D., et al., *Post-exertional malaise among people with long COVID compared to myalgic encephalomyelitis/chronic fatigue syndrome (ME/CFS).* Work, 2023. **74**(4): p. 1179-1186.
16. NINDS, *Common Data Elements (CDE) Group Post-Exertional Malaise Subgroup Summary on Myalgic/Encephalomyelitis/Chronic Fatigue Syndrome*. 2017.
17. Glaab, T. and C. Taube, *Practical guide to cardiopulmonary exercise testing in adults.* Respir Res, 2022. **23**(1): p. 9.
18. Molinger, J., *Feasibility of a Novel Augmented 6-Minute Incremental Step Test: a Simplified Cardiorespiratory Fitness Assessment Tool* 2024, Duke University Medical Center
19. Appelman, B., et al., *Muscle abnormalities worsen after post-exertional malaise in long COVID.* Nat Commun, 2024. **15**(1): p. 17.
20. Garcia, B.J., *Long COVID patients do not meet their nutritional requirements* in *ESPEN*. 2023: Lyon.
21. Barrea, L., et al., *Dietary Recommendations for Post-COVID-19 Syndrome.* Nutrients, 2022. **14**(6).
22. Paul, B.D., et al., *Redox imbalance links COVID-19 and myalgic encephalomyelitis/chronic fatigue syndrome.* Proc Natl Acad Sci U S A, 2021. **118**(34).
23. Lin, M.T. and M.F. Beal, *Mitochondrial dysfunction and oxidative stress in neurodegenerative diseases.* Nature, 2006. **443**(7113): p. 787-95.
24. Zong, Y., et al., *Mitochondrial dysfunction: mechanisms and advances in therapy.* Signal Transduct Target Ther, 2024. **9**(1): p. 124.
25. Ortona, E. and W. Malorni, *Long COVID: to investigate immunological mechanisms and sex/gender related aspects as fundamental steps for tailored therapy.* Eur Respir J, 2022. **59**(2).
26. Hall, D.L., et al., *Stress management skills, cortisol awakening response, and post-exertional malaise in Chronic Fatigue Syndrome.* Psychoneuroendocrinology, 2014. **49**: p. 26-31.
27. Morris, G. and M. Maes, *A neuro-immune model of Myalgic Encephalomyelitis/Chronic fatigue syndrome.* Metab Brain Dis, 2013. **28**(4): p. 523-40.
28. Askren, M.K., et al., *Neuromarkers of fatigue and cognitive complaints following chemotherapy for breast cancer: a prospective fMRI investigation.* Breast Cancer Res Treat, 2014. **147**(2): p. 445-55.

29. El-Rhermoul, F.Z., et al., *Autoimmunity in Long Covid and POTS.* Oxf Open Immunol, 2023. **4**(1): p. iqad002.
30. Amekran, Y., N. Damoun, and A.J. El Hangouche, *Postural orthostatic tachycardia syndrome and post-acute COVID-19.* Glob Cardiol Sci Pract, 2022. **2022**(1-2): p. e202213.
31. Blitshteyn, S. and S. Whitelaw, *Postural orthostatic tachycardia syndrome (POTS) and other autonomic disorders after COVID-19 infection: a case series of 20 patients.* Immunol Res, 2021. **69**(2): p. 205-211.
32. Carmona-Torre, F., et al., *Dysautonomia in COVID-19 Patients: A Narrative Review on Clinical Course, Diagnostic and Therapeutic Strategies.* Front Neurol, 2022. **13**: p. 886609.
33. O'Connor, P.J. and T.W. Puetz, *Chronic physical activity and feelings of energy and fatigue.* Med Sci Sports Exerc, 2005. **37**(2): p. 299-305.
34. Vollestad, N.K. and A.M. Mengshoel, *Post-exertional malaise in daily life and experimental exercise models in patients with myalgic encephalomyelitis/chronic fatigue syndrome.* Front Physiol, 2023. **14**: p. 1257557.
35. Gary Liguori, A.C.o.S.M., *ACSM's Guidelines for Exercise Testing and Prescription* 11th ed. 2021.
36. Oshima, T., et al., *Indirect calorimetry in nutritional therapy. A position paper by the ICALIC study group.* Clin Nutr, 2017. **36**(3): p. 651-662.
37. Ubbink, R., et al., *A monitor for Cellular Oxygen METabolism (COMET): monitoring tissue oxygenation at the mitochondrial level.* J Clin Monit Comput, 2017. **31**(6): p. 1143-1150.
38. Mik, E.G., et al., *In vivo mitochondrial oxygen tension measured by a delayed fluorescence lifetime technique.* Biophys J, 2008. **95**(8): p. 3977-90.
39. Harms, F.A., *Validation of the protoporphyrin IX–triplet state lifetime technique for mitochondrial oxygen measurements in the skin.* Optics Letters 2012. **37**(13): p. 2625-2627.
40. Harms, F.A., et al., *Cutaneous respirometry by dynamic measurement of mitochondrial oxygen tension for monitoring mitochondrial function in vivo.* Mitochondrion, 2013. **13**(5): p. 507-14.
41. Healthcare, P. *Oxygen in tissue cells*. 2017 2024; Available from: https://www.photonicshealthcare.com/#:~:text=Video%20Transcript-

,COMET%20MEASUREMENT%20SYSTEM,the%20mitochondria%20of%20active%20cells.
42. Van de Poppe, D.J., et al., *Reference values for maximum work rate in apparently healthy Dutch/Flemish adults: data from the LowLands fitness registry.* Acta Cardiol, 2019. **74**(3): p. 223-230.
43. *<BORG - 1982.pdf>.*
44. Molinger, J., et al., *Feasibility of a Novel Augmented 6-Minute Incremental Step Test.* JACC: Advances, 2024. **3**(8).

17 Attachments

Survey Prescreening

1. *Taal/Langue/Language*

In welke taal zou u willen deelnemen?
Dans quelle langue souhaitez-vous participer ?
In which language would you participate?

2. *Leeftijd/Âge/Age*

Welke leeftijd heeft u?
Vous avez quel âge ?
How old are you?

3. *SARS-Cov-2 infection*

Werd uw SARS-Cov-2 infectie bevestigd?
Votre infection SARS-Cov-2 a-t-elle été confirmée ?
Was your SARS-Cov-2 infection confirmed?

4. *SARS-Cov-2 infection begin/début/beginning*

Wanneer is uw SARS-Cov-2 infectie begonnen?
Quand est-ce que votre infection SARS-Cov-2 a-t-elle commencé ?
When did your SARS-Cov-2 infection started?

5. *Aanhoudende symptomen/Symptômes persistans/Persisting symptoms*

Welke aanhoudende symptomen ervaart u?
Quels symptômes persistants ressentez-vous?
Which persisting symptoms do you experience?

6. *PESE*

Ervaart u verergering van symptomen na een fysieke/mentale/emotionele inspanning?
Vous souffrez d'exacerbations de symptômes après un effort physique/mental/emotionnel ?
Do you suffer from symptom exacerbations after a physical/mental/emotional effort?

7. *Duur symptomen/Durée symptômes/Duration of symptoms*

Hoelang ervaart u reeds deze aanhoudende symptomen?
Ça fait combien de temps que vous souffrez de ces symptômes persistants ?
How long have you suffered from these persisting symptoms?

8. *Fysieke activiteit/Activité physique/Physical activity*

Voor uw SARS-Cov-2 infectie, was u fysiek actief? Zo ja, wat deed u voor activiteit en hoeveel keer per week?
Avant votre infection SARS-Cov-2, pratiquiez-vous une activité physique régulière ? Si oui, quelle activité physique et combien de fois par semaine ?

Before your SARS-Cov-2 infection, were you physically active? If so, which physical activity did you do and how many times a week?

9. *Allergieën/Allergies*

Heeft u allergieën?
Vous avez des allergies ?
Do you have any allergies?

10. *Mutualiteit/Mutuel/Social security*

Bent u aangesloten bij een mutualiteit?
Êtes-vous affilié à un système de sécurité sociale ?
Are you affiliated to a social security system?

Screening consultatie

Anamnese

Onset klachten:

Huidig klachtenpatroon:

Systeemanamnese:

- Cardiaal (retrosternale pijn, hartkloppingen, oedeemvorming, vagale syncopes…):

- Respiratoir (kortademigheid al dan niet bij inspanning…):

- Abdominaal:

- Andere recente problemen:

Medische geschiedenis:

Hospitalisatie in kader van COVID-infectie:

Medicatie:

Sociaal:

- Opleidingsniveau:

- Werk:

- Vrije tijd/Sport:

- Roken:

- Alcohol:

Klinisch onderzoek

Gewicht:

Lengte:

Bloeddruk:

Hartfrequentie (in rust):

BMI:

Aanvullende onderzoeken

Cardiorespiratoir (ECG...):

Medische beeldvorming:

Besluit

Patiënt/e met aanhoudende klachten, voornamelijk:

Op basis van anamnese en klinisch onderzoek kan hij/zij deelnemen aan deze studie en een maximale inspanningstest onder de vorm van een CPET/6MIST afleggen.

Op basis van anamnese en klinisch onderzoek verwijzen we patiënt/e door naar de dienst Cardiologie en/of Pneumologie voor een verdere evaluatie alvorens een maximale inspanningstest onder de vorm van een CPET/6MIST aan te vatten.

Tampa Schaal voor Kinesiofobie/Tampa Scale for Kinesiophobia/Echelle Tampa

TAMPA-SCHAAL VOOR KINESIOFOBIE

Miller, RP., Kori, SH & Todd, DD.(1991)

Geautoriseerde Nederlandse Vertaling

Vlaeyen J.W.S., Kole-Snijders A.M.J., Crombez, G. Boeren R.G.B. & Rotteveel, A.M.(1995)

INSTRUCTIE:

Met deze lijst willen wij onderzoeken op welke wijze u tegen uw pijn aankijkt en hoe u deze ervaart.
Het is de bedoeling dat u met behulp van de cijfers 1 t/m 4 aangeeft in welke mate u het eens of oneens bent met elke bewering. Het is van essentieel belang dat u bij de beoordeling uitgaat van uw eigen gevoelens; wat anderen denken is hierbij niet relevant.
Het is ook niet de bedoeling uw medische kennis te testen. Waar het om gaat is dat u aangeeft hoe u uw pijn ervaart.

Geef van onderstaande beweringen door middel van een cijfer tussen 1 en 4 aan in welke mate u het eens of oneens bent met deze bewering. De betekenis van de cijfers is als volgt:

1 = in hoge mate mee oneens
2 = enigszins mee oneens
3 = enigszins mee eens
4 = in hoge mate mee eens

1.	Ik ben bang om bij het doen van lichaamsoefeningen letsel op te lopen.	1	2	3	4
2.	Als ik me over de pijn heen zou zetten, dan zou hij erger worden.	1	2	3	4
3.	Mijn lichaam zegt me dat er iets gevaarlijks mis mee is.	1	2	3	4
4.	Mijn pijn zou waarschijnlijk minder worden als ik lichaams-oefeningen zou doen.	1	2	3	4
5.	Mijn gezondheidstoestand wordt door anderen niet serieus genoeg genomen.	1	2	3	4
6.	Door mijn pijnproblemen loopt mijn lichaam de rest van mijn leven gevaar.	1	2	3	4
7.	Mijn pijn betekent dat er sprake is van letsel.	1	2	3	4
8.	Als mijn pijn erger wordt door iets, betekent dat nog niet dat dat gevaarlijk is.	1	2	3	4
9.	Ik ben bang om per ongeluk letsel op te lopen.	1	2	3	4
10.	De veiligste manier om te voorkomen dat mijn pijn erger wordt is gewoon oppassen dat ik geen onnodige bewegingen maak.	1	2	3	4
11.	Ik had wellicht minder pijn als er niet iets gevaarlijks aan de hand zou zijn met mijn lichaam.	1	2	3	4
12.	Hoewel ik pijn heb, zou ik er beter aan toe zijn als ik lichamelijk actief zou zijn.	1	2	3	4
13.	Mijn pijn zegt me wanneer ik moet stoppen met lichaams-oefeningen doen om geen letsel op te lopen.	1	2	3	4
14.	Voor iemand in mijn toestand is het echt af te raden om lichamelijk actief te zijn.	1	2	3	4
15.	Ik kan niet alles doen wat gewone mensen doen, omdat ik te gemakkelijk letsel oploop.	1	2	3	4
16.	Zelfs als ik ergens veel pijn door krijg, geloof ik niet dat dat gevaarlijk is	1	2	3	4
17.	Ik zou geen lichaamsoefeningen hoeven doen wanneer ik pijn heb.	1	2	3	4

Tampa Scale for Kinesiophobia
(Miller , Kori and Todd 1991)

1 = strongly disagree
2 = disagree
3 = agree
4 = strongly agree

1. I'm afraid that I might injury myself if I exercise	1	2	3	4
2. If I were to try to overcome it, my pain would increase	1	2	3	4
3. My body is telling me I have something dangerously wrong	1	2	3	4
4. My pain would probably be relieved if I were to exercise	1	2	3	4
5. People aren't taking my medical condition seriously enough	1	2	3	4
6. My accident has put my body at risk for the rest of my life	1	2	3	4
7. Pain always means I have injured my body	1	2	3	4
8. Just because something aggravates my pain does not mean it is dangerous	1	2	3	4
9. I am afraid that I might injure myself accidentally	1	2	3	4
10. Simply being careful that I do not make any unnecessary movements is the safest thing I can do to prevent my pain from worsening	1	2	3	4
11. I wouldn't have this much pain if there weren't something potentially dangerous going on in my body	1	2	3	4
12. Although my condition is painful, I would be better off if I were physically active	1	2	3	4
13. Pain lets me know when to stop exercising so that I don't injure myself	1	2	3	4
14. It's really not safe for a person with a condition like mine to be physically active	1	2	3	4
15. I can't do all the things normal people do because it's too easy for me to get injured	1	2	3	4
16. Even though something is causing me a lot of pain, I don't think it's actually dangerous	1	2	3	4
17. No one should have to exercise when he/she is in pain	1	2	3	4

ÉCHELLE TAMPA (TSK-CF)

(Kori et al., 1990, traduite par GRISART & MASQUELIER, Cliniques Universitaires Saint-Luc, 1200 Bruxelles)

Même à cette époque de haute technologie, il ne faut pas négliger une des plus importantes sources d'information à votre sujet : il s'agit de vos sentiments ou de vos intuitions à propos de ce qui arrive à votre corps.
Répondez aux questions suivantes en utilisant l'échelle de droite. Répondez vraiment en fonction de votre impression et pas en fonction de ce que les autres pensent que vous devriez croire. Il ne s'agit pas d'un test de connaissance médicale. Nous voulons savoir comment vous voyez les choses.

	Consignes : Veuillez lire attentivement chaque question **et encercler le numéro qui correspond le mieux à ce que vous ressentez.**	Fortement en désaccord	Légèrement en désaccord	Légèrement en accord	Fortement en accord
1.	J'ai peur qu'en faisant de l'exercice, cela ne me blesse.	1	2	3	4
2.	Si je vais au-delà de mes limites pour dépasser la douleur, elle pourrait augmenter.	1	2	3	4
3.	Mon corps me dit que quelque chose ne va pas et que cela constitue un danger pour lui.	1	2	3	4
4.	Ma douleur serait probablement diminuée si je faisais de l'exercice. *	1	2	3	4
5.	Les gens ne prennent pas mon état de santé suffisamment au sérieux.	1	2	3	4
6.	Mon accident a fragilisé mon corps pour le reste de ma vie.	1	2	3	4
7.	La douleur signifie qu'il y a toujours une lésion.	1	2	3	4
8.	Ce n'est parce que quelque chose aggrave ma douleur que cela signifie que c'est dangereux *	1	2	3	4
9.	J'ai peur de me faire mal ou de me blesser par mégarde.	1	2	3	4
10.	En étant attentif à ne faire que des gestes adéquats, je peux éviter d'augmenter la douleur.	1	2	3	4
11.	Le maintien d'une telle douleur signifie que j'ai probablement quelque chose de grave.	1	2	3	4
12.	Malgré ma douleur, je serais mieux si j'avais plus d'activités physiques. *	1	2	3	4
13.	La douleur me fait savoir quand je dois arrêter mes exercices afin de ne pas entraîner des dommages corporels.	1	2	3	4
14.	Ce n'est pas vraiment très bon pour une personne dans un état comme le mien d'être physiquement active.	1	2	3	4
15.	Je ne peux pas tout faire comme les autres, sinon cela pourrait causer des lésions dans mon organisme.	1	2	3	4
16.	Même si certaines choses entraînent une douleur, je ne pense pas qu'elles soient réellement dangereuses. *	1	2	3	4
17.	Personne ne devrait faire de l'exercice lorsqu'il(elle) a mal.	1	2	3	4

DePaul Symptom Questionnaire – Post-Exertional Malaise (DSQ-PEM)

Pour chaque symptôme ci-dessous, veuillez entourer un chiffre pour indiquer sa fréquence et un chiffre pour sa gravité :
Veuillez remplir le tableau de gauche à droite.

Symptômes	Fréquence :<br>Au cours des **6 derniers mois, à quelle fréquence** avez-vous eu ce symptôme ?<br>Pour chaque symptôme listé ci-dessous, entourez un chiffre parmi :<br>**0 = jamais**<br>**1 = de temps en temps**<br>**2 = environ la moitié du temps**<br>**3 = la plupart du temps**<br>**4 = tout le temps**	Sévérité :<br>Au cours des **6 derniers mois, à quel degré** ce symptôme vous a-t-il dérangé ?<br>Pour chaque symptôme listé ci-dessous, entourez un chiffre parmi :<br>**0 = symptôme non présent**<br>**1 = faible**<br>**2 = modéré**<br>**3 = sévère**<br>**4 = très sévère**
1. Sensation d'assommement, de lourdeur après avoir débuté un exercice physique	0 1 2 3 4	0 1 2 3 4
2. Douleur ou fatigue le lendemain d'activités ordinaires non intensives	0 1 2 3 4	0 1 2 3 4
3. Fatigué-e mentalement après le moindre effort	0 1 2 3 4	0 1 2 3 4
4. Faire un minimum d'exercice vous fatigue physiquement	0 1 2 3 4	0 1 2 3 4
5. Épuisé-e physiquement ou malade après une activité légère	0 1 2 3 4	0 1 2 3 4

Pour chaque question ci-dessous, choisissez la réponse qui décrit le mieux vos symptômes de malaise post-effort.

6. Si vous étiez épuisé-e après avoir participé activement à des activités extrascolaires, sportives ou à des sorties avec des amis, vous en remettriez-vous en une heure ou deux après la fin de l'activité ?	Oui	Non
7. Ressentez-vous une aggravation de votre **fatigue / maladie liée à l'énergie** après avoir fourni un effort physique minime ?	Oui	Non
8. Ressentez-vous une aggravation de votre **fatigue / maladie liée à l'énergie** après avoir fourni un effort mental ?	Oui	Non

9. Si vous vous sentez moins bien après des activités, combien de temps cela dure-t-il ?	≤ 1 h	2–3 h	4–10 h	11–13 h	14–23 h	≥ 24 h
10. Si vous ne faites pas d'exercice, est-ce parce que l'exercice aggrave vos symptômes ?	Oui			Non		

For each symptom below, please circle one number for frequency and one number for severity:
Please complete the chart from left to right.

Symptoms	Frequency: Throughout the **past 6 months**, **how often** have you had this symptom? For each symptom listed below, circle a number from: **0 = none of the time** **1 = a little of the time** **2 = about half the time** **3 = most of the time** **4 = all of the time**					Severity: Throughout the **past 6 months**, **how much** has this symptom bothered you? For each symptom listed below, circle a number from: **0 = symptom not present** **1 = mild** **2 = moderate** **3 = severe** **4= very severe**				
1. Dead, heavy feeling after starting to exercise	0	1	2	3	4	0	1	2	3	4
2. Next day soreness or fatigue after non-strenuous, everyday activities	0	1	2	3	4	0	1	2	3	4
3. Mentally tired after the slightest effort	0	1	2	3	4	0	1	2	3	4
4. Minimum exercise makes you physically tired	0	1	2	3	4	0	1	2	3	4
5. Physically drained or sick after mild activity	0	1	2	3	4	0	1	2	3	4

For each question below, choose the answer which best describes your PEM symptoms.

6. If you were to become exhausted after actively participating in extracurricular activities, sports, or outings with friends, would you recover within an hour or two after the activity ended?	Yes			No		
7. Do you experience a worsening of your **fatigue/energy related illness** after engaging in minimal **physical** effort?	Yes			No		
8. Do you experience a worsening of your **fatigue/energy related illness** after engaging in minimal **mental** effort?	Yes			No		
9. If you feel worse after activities, how long does this last?	≤1 h	2-3 h	4-10 h	11-13 h	14-23 h	≥24 h
10. If you do not exercise, is it because exercise makes your symptoms worse?	Yes			No		

Voor elk symptoom hieronder, markeer één nummer voor de frequentie en één nummer voor de ernst. Omcirkel het nummer tussen 0 en 4 dat het beste van toepassing is bij het gevoel dat u ervaart. Vul de tabel van links naar rechts in.

	**Frequentie**					**Ernst**				
**Symptomen**	Gedurende de afgelopen **6 maanden**, hoe vaak heeft u dit symptoom ervaren?<br><br>Voor elk hieronder vermeld symptoom, markeer één nummer van:<br><br>0 = nooit<br>1 = af en toe<br>2 = ongeveer de helft van de tijd<br>3 = het grootste gedeelte van de tijd<br>4 = altijd					Gedurende de afgelopen **6 maanden**, hoeveel last heeft dit symptoom u bezorgd?<br>Voor elk hieronder vermeld symptoom, markeer één nummer van:<br><br>0 = symptoom niet aanwezig<br>1 = mild<br>2 = matig<br>3 = ernstig<br>4 = zeer ernstig				
**1. Dood, zwaar gevoel na het starten met fysieke inspanning**	0	1	2	3	4	0	1	2	3	4
**2. (Spier)pijn of vermoeidheid de volgende dag na niet-inspannende alledaagse activiteiten**	0	1	2	3	4	0	1	2	3	4
**3. Mentaal vermoeid na de geringste fysieke of mentale inspanning**	0	1	2	3	4	0	1	2	3	4
**4. Minimale lichaamsbeweging maakt u fysiek moe**	0	1	2	3	4	0	1	2	3	4
**5. Fysiek uitgeput of ziek na lichte inspanning**	0	1	2	3	4	0	1	2	3	4

Voor elke vraag hieronder, kies het antwoord dat het beste jouw symptomen beschrijft.

**6. Als u uitgeput raakte na actieve deelname aan buitenschoolse activiteiten, sport of uitstapjes met vrienden, herstelt u dan binnen een uur of twee na het beëindigen van de activiteit?**	Ja			Nee		
**7. Ervaart u een verergering van uw vermoeidheid/energie gerelateerde aandoening na het verrichten van minimale fysieke inspanning?**	Ja			Nee		
**8. Ervaar u een verslechtering van uw vermoeidheid/energie gerelateerde aandoening na het verrichten van minimale mentale inspanning?**	Ja			Nee		
**9. Als u zich slechter voelt na activiteiten, hoelang duurt dit?**	≤1u	2-3u	4-10u	11-13u	14-23u	≥24u
**10. Als u niet sport, is dat omdat lichaamsbeweging uw symptomen verergert?**	Ja			Nee		

Brief Resilience Scale/Korte Veerkracht Vragenlijst/Echelle Brève Résilience

Brief Resilience Scale (BRS)

Respond to each statement below by circling <u>one answer per row.</u>		Strongly Disagree	Disagree	Neutral	Agree	Strongly Agree
**BRS 1**	I tend to bounce back quickly after hard times.	1	2	3	4	5
**BRS 2**	I have a hard time making it through stressful events.	5	4	3	2	1
**BRS 3**	It does not take me long to recover from a stressful event.	1	2	3	4	5
**BRS 4**	It is hard for me to snap back when something bad happens.	5	4	3	2	1
**BRS 5**	I usually come through difficult times with little trouble.	1	2	3	4	5
**BRS 6**	I tend to take a long time to get over setbacks in my life.	5	4	3	2	1

Scoring: Add the value (1-5) of your responses for all six items, creating a range from 6-30. Divide the sum by the total number of questions answered (6) for your final score.

Total score: _____ / 6

My score: _____ (average)

BRS Score	Interpretation
1.00 - 2.99	Low resilience
3.00 - 4.30	Normal resilience
4.31 - 5.00	High resilience

Smith, B.W., Dalen, J., Wiggins, K., Tooley, E., Christopher, P. and Bernard, J. (2008). The Brief Resilience Scale: Assessing the Ability to Bounce Back. *International Journal of Behavioral Medicine*, 15, 194-200.

BRIEF RESILIENCE SCALE AND DUTCH TRANSLATION

Item	English BRS	Dutch language version of the BRS
1	I tend to bounce back quickly after hard times	Na een moeilijke periode veer ik meestal gemakkelijk weer terug
2	I have a hard time making it through stressful events (R)	Ik vind het moeilijk om me door stressvolle gebeurtenissen heen te slaan. (R)
3	It does not take me long to recover from a stressful event	Het kost me weinig tijd om te herstellen van een stressvolle gebeurtenis
4	It is hard for me to snap back when something bad happens (R)	Ik vind het moeilijk om het snel van me af te schudden als er iets ergs is gebeurd. (R)
5	I usually come through difficult times with little trouble	Ik sla me meestal redelijk probleemloos door moeilijke periodes heen.
6	I tend to take a long time to get over set-backs in my life (R)	Het kost me meestal veel tijd om over tegenslagen in mijn leven heen te komen. (R)

Items can be scored on a 5-point Likert scale: 1 = strongly disagree; 2 = disagree; 3 = neutral; 4 = agree; 5 = strongly agree
(R)= Reverse Items (5 = 1, 4 = 2, 3 = 3, 2 = 4, 1 = 5)

Echelle Brève Résilience

Veuillez indiquer pour chaque phrase à quel point celle-ci vous correspond.

1. Non, pas du tout
2. Non, pas vraiment
3. Neutre
4. Plutôt oui
5. Oui, tout à fait

1. Je tends à rebondir rapidement après des moments difficiles.
2. J'ai du mal à traverser des événements stressants.
3. Je me remets facilement d'un événement stressant.
4. Il est difficile pour moi de revenir brusquement à la réalité quand quelque chose se passe mal.
5. En général je traverse les moments difficiles sans trop de difficulté.
6. J'ai tendance à prendre beaucoup de temps pour me remettre des revers dans ma vie.

(Revised-)Piper Fatigue Scale/Echelle de Fatigue Revisée de Piper

PIPER FATIGUE SCALE

Toelichting: Omcirkel voor elk van de volgende vragen het cijfer dat het beste de vermoeidheid beschrijft, die u <u>op dit moment</u> ervaart. Probeer alstublieft elke vraag zo goed mogelijk te beantwoorden. Hartelijk dank.

1. In welke mate baart de vermoeidheid die u op dit moment ervaart, u zorgen?

**geen zorgen**										**veel zorgen**
0	1	2	3	4	5	6	7	8	9	10

2. In welke mate beperkt de vermoeidheid die u op dit moment ervaart, uw vermogen tot het uitvoeren van uw dagelijkse bezigheden (als werk, huishouden, school en dergelijke)?

**helemaal niet**										**zeer sterk**
0	1	2	3	4	5	6	7	8	9	10

3. In welke mate beperkt de vermoeidheid die u op dit moment ervaart, uw vermogen tot het bezoeken van, of omgaan met uw vrienden of kennissen?

**helemaal niet**										**zeer sterk**
0	1	2	3	4	5	6	7	8	9	10

4. In welke mate beperkt de vermoeidheid die u op dit moment ervaart, uw vermogen tot sexuele activiteit?

**helemaal niet**										**zeer sterk**
0	1	2	3	4	5	6	7	8	9	10

5. Al met al, in welke mate beperkt de vermoeidheid die u op dit moment ervaart, uw vermogen dingen te doen te doen die u leuk vindt?

**helemaal niet**										**zeer sterk**
0	1	2	3	4	5	6	7	8	9	10

6. Hoe zou u de intensiteit of de ernst van de vermoeidheid beschrijven, die u op dit moment voelt?

**mild**										**ernstig**
0	1	2	3	4	5	6	7	8	9	10

In welke mate zou u de vermoeidheid die u op dit moment ervaart beschrijven als:

7. **aangenaam** — **onaangenaam**

0	1	2	3	4	5	6	7	8	9	10

8. **aanvaardbaar** — **onaanvaardbaar**

0	1	2	3	4	5	6	7	8	9	10

9. **beschermend** — **slopend**

0	1	2	3	4	5	6	7	8	9	10

10. **positief** — **negatief**

0	1	2	3	4	5	6	7	8	9	10

Geautoriseerde Nederlandse versie, © Vakgroep Epidemiologie, Universiteit Maastricht

11. **normal** **abnormaal**
0 1 2 3 4 5 6 7 8 9 10

12. In welke mate voelt u zich op dit moment:
sterk **zwak**
0 1 2 3 4 5 6 7 8 9 10

13. In welke mate voelt u zich op dit moment:
wakker **slaperig**
0 1 2 3 4 5 6 7 8 9 10

14. In welke mate voelt u zich op dit moment:
levendig **lusteloos**
0 1 2 3 4 5 6 7 8 9 10

15. In welke mate voelt u zich op dit moment:
opgefrist **vermoeid**
0 1 2 3 4 5 6 7 8 9 10

16. In welke mate voelt u zich op dit moment:
energiek **futloos**
0 1 2 3 4 5 6 7 8 9 10

17. In welke mate voelt u zich op dit moment:
geduldig **ongeduldig**
0 1 2 3 4 5 6 7 8 9 10

18. In welke mate voelt u zich op dit moment:
ontspannen **gespannen**
0 1 2 3 4 5 6 7 8 9 10

19. In welke mate voelt u zich op dit moment:
opgewekt **neerslachtig**
0 1 2 3 4 5 6 7 8 9 10

20. In welke mate voelt u zich op dit moment:
in staat zich te concentreren **niet in staat zich te concentreren**
0 1 2 3 4 5 6 7 8 9 10

21. In welke mate voelt u zich op dit moment:
in staat zich dingen te herinneren **niet in staat zich dingen te herinneren**
0 1 2 3 4 5 6 7 8 9 10

PIPER FATIGUE SCALE

22. In welke mate voelt u zich op dit moment:

**in staat helder te denken**										**niet in staat helder te denken**
0	1	2	3	4	5	6	7	8	9	10

23. Al met al, wat is volgens u de belangrijkste oorzaak van uw vermoeidheid?

____

____

24. Al met al, wat is het beste middel dat u gevonden hebt om uw vermoeidheid te verminderen?

____

____

25. Wilt u nog iets anders toevoegen dat uw vermoeidheid voor ons beter beschrijft?

____

____

26. Ervaart u op dit moment nog andere symptomen / verschijnselen?

- ☐ Nee
- ☐ Ja, namelijk ______________________________

____

Date:_____________ Qualifying Assessment ID

PIPER FATIGUE SCALE (PFS)

Directions: Many individuals can experience a sense of unusual or excessive tiredness whenever they become ill, receive treatment, or recover from their illness/treatment. This unusual sense of tiredness is not usually relieved by either a good night's sleep or by rest. Some call this symptom "fatigue" to distinguish it from the usual sense of tiredness.

For each of the following questions, please fill in the space provided for that response that best describes the fatigue you are experiencing now or for today. Please make every effort to answer each question to the best of your ability. If you are not experiencing fatigue now or for today, fill in the circle indicating "0" for your response. Thank you very much!

1. How long have you been feeling fatigue? (Check one response only).

    1. not feeling fatigue
    2. minutes
    3. hours
    4. days
    5. weeks
    6. months
    7. other (Please describe) ____

2. To what degree is the fatigue you are feeling now causing you distress?

No Distress A Great Deal

1 2 3 4 5 6 7 8 9 10

3. To what degree is the fatigue you are feeling now interfering with your ability to complete your work or school activities?

None A Great Deal

1 2 3 4 5 6 7 8 9 10

4. To what degree is the fatigue you are feeling now interfering with your ability to socialize with your friends?

None A Great Deal

1 2 3 4 5 6 7 8 9 10

5. To what degree is the fatigue you are feeling now interfering with your ability to engage in sexual activity?

None | A Great Deal

1 2 3 4 5 6 7 8 9 10

6. Overall, how much is the fatigue which you are now experiencing interfering with your ability to engage in the kind of activities you enjoy doing?

None | A Great Deal

1 2 3 4 5 6 7 8 9 10

7. How would you describe the degree of intensity or severity of the fatigue which you are experiencing now?

Mild | Severe

1 2 3 4 5 6 7 8 9 10

8. To what degree would you describe the fatigue which you are experiencing now as being?

Pleasant | Unpleasant

1 2 3 4 5 6 7 8 9 10

9. To what degree would you describe the fatigue which you are experiencing now as being?

Agreeable | Disagreeable

1 2 3 4 5 6 7 8 9 10

10. To what degree would you describe the fatigue which you are experiencing now as being?

Protective | Destructive

1 2 3 4 5 6 7 8 9 10

11. To what degree would you describe the fatigue which you are experiencing now as being?

Positive | Negative

1 2 3 4 5 6 7 8 9 10

12. To what degree would you describe the fatigue which you are experiencing now as being:

Normal Abnormal

1 2 3 4 5 6 7 8 9 10

13. To what degree are you now feeling:

Strong Weak

1 2 3 4 5 6 7 8 9 10

14. To what degree are you now feeling:

Awake Sleepy

1 2 3 4 5 6 7 8 9 10

15. To what degree are you now feeling:

Lively Listless

1 2 3 4 5 6 7 8 9 10

16. To what degree are you now feeling:

Refreshed Tired

1 2 3 4 5 6 7 8 9 10

17. To what degree are you now feeling:

Energetic Unenergetic

1 2 3 4 5 6 7 8 9 10

18. To what degree are you now feeling:

Patient Impatient

1 2 3 4 5 6 7 8 9 10

19. To what degree are you now feeling:

Relaxed A Great Deal

1 2 3 4 5 6 7 8 9 10

20. To what degree are you now feeling:

Exhilarated Depressed

1 2 3 4 5 6 7 8 9 10

21. To what degree are you now feeling:

Able to Concentrate Unable to Concentrate

1 2 3 4 5 6 7 8 9 10

22. To what degree are you now feeling:

Able to Remember Unable to Remember

1 2 3 4 5 6 7 8 9 10

23. To what degree are you now feeling:

Able to Think Clearly Unable to Think Clearly

1 2 3 4 5 6 7 8 9 10

24. Overall, what do you believe is ***most*** directly contributing to or causing your fatigue?
____
____
____

25. Overall, the ***best*** thing you have found to relieve your fatigue is: ____________________
____
____
____

26. Is there anything else you would like to add that would describe your fatigue better to us?
____
____
____

27. Are you experiencing any other symptoms right now? ____________________________
____
____
____

Annexe 2. Questionnaire de Fatigue de Piper. Version testée.

ÉCHELLE DE FATIGUE RÉVISÉE DE PIPER (1998)*

Recommandations : Les questions suivantes concernent la fatigue que vous éprouvez **en ce moment**. Répondez à toutes les questions du mieux que vous pouvez et notez le temps que vous avez mis à la fin du questionnaire. Nous vous en remercions.

1. Vous sentez-vous fatigué(e) **en ce moment**? **OUI** ☐ **NON** ☐

Si OUI

Depuis combien de temps vous sentez-vous fatigué(e)?
Chiffrez et cochez **une seule réponse.**

a) ....Jours ....☐

b) ....Semaines ....☐

c) ....Mois ....☐

d) ....Autres (précisez) : ....

Pour les questions qui suivent, entourez, dans la ligne des chiffres, celui qui correspond le mieux à votre état de fatigue en ce moment.

2. A quel point la fatigue que vous ressentez en ce moment est-elle une souffrance ou un souci pour vous?

Pas du tout **Énormément**

0 1 2 3 4 5 6 7 8 9 10

3. La fatigue que vous ressentez en ce moment affecte-t-elle votre capacité à travailler ou à suivre une activité scolaire?

Pas du tout **Énormément**

0 1 2 3 4 5 6 7 8 9 10

4. La fatigue que vous ressentez en ce moment affecte-t-elle vos possibilités de sortir et/ou de passer du temps avec vos amis?

Pas du tout **Énormément**

0 1 2 3 4 5 6 7 8 9 10

5. La fatigue que vous ressentez en ce moment perturbe-t-elle votre capacité à avoir une activité sexuelle?

Pas du tout **Énormément**

0 1 2 3 4 5 6 7 8 9 10

6. Dans l'ensemble votre fatigue actuelle affecte-t-elle votre capacité à profiter des choses auxquelles normalement vous prenez plaisir?

Pas du tout **Énormément**

0 1 2 3 4 5 6 7 8 9 10

7. Quelle est l'intensité ou la sévérité de la fatigue que vous ressentez **en ce moment?**

Légère **Forte**

0 1 2 3 4 5 6 7 8 9 10

8. La fatigue que vous éprouvez **en ce moment** est-elle :

Plaisante **Déplaisante**

0 1 2 3 4 5 6 7 8 9 10

9. La fatigue que vous éprouvez **en ce moment** est-elle :

Agréable **Désagréable**

0 1 2 3 4 5 6 7 8 9 10

10. La fatigue que vous éprouvez **en ce moment** est-elle :

Protectrice **Destructrice**

0 1 2 3 4 5 6 7 8 9 10

11. La fatigue que vous éprouvez **en ce moment** est-elle :

Positive **Négative**

0 1 2 3 4 5 6 7 8 9 10

12. La fatigue que vous éprouvez **en ce moment** est-elle :

Normale **Anormale**

0 1 2 3 4 5 6 7 8 9 10

13. Comment vous sentez vous **en ce moment**

Fort(e) **Faible**

0 1 2 3 4 5 6 7 8 9 10

14. Comment vous sentez vous **en ce moment**?

Bien réveillé(e) **Endormi(e)**

0 1 2 3 4 5 6 7 8 9 10

15. Comment vous sentez vous **en ce moment**?

Dynamique — **Vide, sans entrain**

0 1 2 3 4 5 6 7 8 9 10

16. Comment vous sentez vous **en ce moment**?

Reposé(e) — **Fatigué(e)**

0 1 2 3 4 5 6 7 8 9 10

17. Comment vous sentez vous **en ce moment**?

Énergique — **Sans énergie**

0 1 2 3 4 5 6 7 8 9 10

18. Comment vous sentez vous **en ce moment**?

Patient(e) — **Impatient(e)**

0 1 2 3 4 5 6 7 8 9 10

19. Comment vous sentez vous **en ce moment**?

Détendu(e) — **Tendu(e)**

0 1 2 3 4 5 6 7 8 9 10

20. Comment vous sentez vous **en ce moment**?

Gai (e) — **Déprimé(e)**

0 1 2 3 4 5 6 7 8 9 10

21. Comment vous sentez vous **en ce moment**?

Capable de vous concentrer — **Incapable de vous concentrer**

0 1 2 3 4 5 6 7 8 9 10

22. Comment vous sentez vous **en ce moment**?

Capable de vous souvenir — **Incapable de vous souvenir**

0 1 2 3 4 5 6 7 8 9 10

23. Comment vous sentez vous **en ce moment**?

Capable de réfléchir — **Incapable de réfléchir**

0 1 2 3 4 5 6 7 8 9 10

24. Globalement, quelle est selon vous la cause la plus directe de votre fatigue?

....

....

....

25. Globalement, ce qui s'est avéré le plus efficace pour soulager votre fatigue est :

....

....

....

26. Y a-t-il d'autres termes que vous aimeriez ajouter pour mieux nous décrire **votre** fatigue?

....

....

....

27. Avez vous d'autres symptômes en ce moment?

NON ☐

OUI ☐

Si OUI, lesquels :

....

....

....

Calcul du score :

La dimension comportementale = 6 items : #2 – 7

La dimension affective = 5 items : #8 – 12

La dimension sensorielle = 5 items : #13 – 17

La dimension cognitive/humeur = 6 items : #18 – 23

Pour chaque malade les résultats sont exprimés sous la forme d'un score attribué pour chaque dimension et d'un score total.
Le score de chaque dimension est calculé en faisant la somme des notes données par le patient pour les items correspondants, divisée par le nombre d'items de cette dimension.
Le score total de fatigue est calculé en faisant la somme des scores obtenus pour chaque dimension divisée par le nombre de dimensions explorées. (4)
Le score obtenu pour chaque dimension est comparé au score total de fatigue, et permet ainsi de classer les résultats en plusieurs types de fatigue en fonction de chaque composante. Ce sont les prédominances d'une ou plusieurs caractéristiques qui permettront de déterminer l'intervention appropriée pour le patient pour diminuer son niveau de fatigue.

*Traduit de PIPER (B.F.), DIBBLE (S.L.), DODD (M.J.), WEISS (M.C.), SLAUGHTER (R.E.), & PAUL (S. M) (1998). The revised Piper Fatigue Scale : Psychometric evaluation in women with breast cancer. Oncol Nurs Forum, 25 (4) : 677 – 684.

10-point Likert scale

Op een schaal van 1 tot 10, waarbij 1 'geen ongemak' en 10 'ernstig ongemak' betekent, hoe zou u uw huidige niveau van neuromusculaire klachten (bijv. spierpijn, zwakte, krampen, gevoelloosheid, gewrichtspijn,...)?

Helemaal niet akkoord | Helemaal akkoord

1	2	3	4	5	6	7	8	9	10

Sur une échelle de 1 à 10, où 1 indique 'aucun inconfort' et 10 indique 'inconfort sévère', comment évalueriez-vous votre niveau actuel de plaintes neuromusculaires (par ex, douleur musculaire, faiblesse, crampes, engourdissement, douleur articulaire,...) ?

Pas du tout d'accord | Tout à fait d'accord

1	2	3	4	5	6	7	8	9	10

On a scale from 1 to 10, where 1 indicates 'no discomfort at all' and 10 indicates 'severe discomfort', how would you rate your current level of neuromuscular complaints (e.g., muscle pain, weakness, cramps, numbness, joint pain,...)?

Strongly disagree | Strongly agree

1	2	3	4	5	6	7	8	9	10

Borgschaal® (RPE)

Gebruik deze schaal om aan te geven hoe inspannend en vermoeiend je de activiteit vindt. Je merkt de inspanning vooral als vermoeidheid in je spieren, ademloosheid en mogelijk pijn. Valt de lichaamsbeweging je zwaar, dan wordt het ook moeilijk om te praten. Het gaat om hoe inspannend jij de lichaamsbeweging vindt. Het is belangrijk dat je je inspanning niet onderschat of overschat. Voor gewone lichaamsbeweging, zoals fietsen, hardlopen en wandelen, is 11-15 een goede score. Voor krachttraining en intensieve intervaltraining is 15-19 een normale score. Volg het advies van je arts als je ziek bent. Bekijk de schaal en de beschrijvingen en kies een getal. Je mag elk getal kiezen, ook als het tussen twee beschrijvingen staat.

**6**	**Geen enkele inspanning**	Geen spiervermoeidheid, niet buiten adem, geen
**7**	**Extreem licht**	Uiterst beperkt.
**8**		
**9**	**Heel licht**	Als een korte, rustige wandeling. Heel makkelijk om te praten.
**10**		
**11**	**Licht**	Als een lichte fysieke activiteit op je eigen tempo.
**12**	**Matig**	
**13**	**Vrij zwaar**	Enigszins vermoeiend en buiten adem. Niet zo makkelijk om te
**14**		
**15**	**Zwaar**	Zwaar en inspannend. Een bovengrens voor fitnesstraning, zoals hardlopen, snel wandelen.
**16**		
**17**	**Heel zwaar**	Heel inspannend. Je bent erg moe en buiten adem. Heel moeilijk om
**18**		
**19**	**Extreem zwaar**	De zwaarste inspanning die je ooit hebt geleverd.
**20**	**Maximale inspanning**	Maximale inspanning.

Borgschaal® (RPE)
Ratings (R) of Perceived (P) Exertion (E).
© Gunnar Borg, 1970, 1998, 2017
Vlaams, Flemish

Borg RPE Échelle® Perception de l'effort

Évaluez votre sensation de l'effort, le degré de pénibilité et de fatigue que vous ressentez. Celui-ci se traduit essentiellement par une fatigue musculaire, un essoufflement et, éventuellement, des douleurs. Lorsque l'exercice est lourd, il devient également difficile de parler. C'est votre propre sentiment d'effort qui est important. Tentez de ne pas sous-estimer ni surestimer la fatigue. Pour un exercice régulier, comme le vélo, la course ou la marche rapide, 11-15 est un bon niveau. Pour la force et l'entraînement par intervalles à haute intensité (HIIT), 15-19 est bon. Si vous êtes malade, suivez les conseils de votre médecin. Lisez les descriptions puis sélectionnez un chiffre. Utilisez les chiffres de votre choix sur l'échelle, pas forcément ceux en face de la description.

**6**	**Pas de fatigue du tout**	Aucune fatigue musculaire, essoufflement ou difficulté à respirer.
**7**	**Extrêmement léger**	Très peu fatigant.
**8**		
**9**	**Très leger**	Similaire à une marche lente pendant quelques instants.
**10**		
**11**	**Léger**	Similaire à un exercice facile à votre propre rythme. Très facile de parler.
**12**	**Moderée**	
**13**	**Un peu fatigant**	Assez pénible et essoufflé. Pas si facile de parler.
**14**		
**15**	**Fatigant**	Dur et pénible. La limite supérieure pour l'entraînement physique, par exemple courir ou marcher rapidement.
**16**		
**17**	**Très fatigant**	Très dur et pénible. Vous êtes très fatigué. Très difficile de parler.
**18**		
**19**	**Extrêmement fatigant**	Une des épreuves les plus dures que vous ayez connue.
**20**	**Fatigue maximale**	Charge maximale.

Borg RPE Échelle®
© Gunnar Borg, 1970, 1998, 2017
French, Francais

Borg RPE Scale®

Use this scale to tell how strenuous and tiring the work feels to you. The exertion is mainly felt as fatigue in your muscles and as breathlessness or possibly aches. When the exercise is hard it also becomes difficult to talk. It is your own feeling of exertion that is important. Don't underestimate it, but don't overestimate it either. For common exercise, such as cycling, running or walking, 11-15 is a good level. For strength and high-intensity interval training (HIIT), 15-19 is good. If you are sick follow your doctor's advice. Look at the scale and the descriptions and then choose a number. Use whatever numbers you want, even numbers between the descriptions.

**6**	**No exertion at all**	No muscle fatigue, breathlessness or difficulty in breathing.
**7**	**Extremely light**	Very, very light.
**8**		
**9**	**Very light**	Like walking slowly for a short while. Very easy to talk.
**10**		
**11**	**Light**	Like a light exercise at your own pace.
**12**	**Moderate**	
**13**	**Somewhat hard**	Fairly strenuous and breathless. Not so easy to talk.
**14**		
**15**	**Hard**	Heavy and strenuous. An upper limit for fitness training, as when running or walking fast.
**16**		
**17**	**Very hard**	Very strenuous. You are very tired and breathless. Very difficult to talk.
**18**		
**19**	**Extremely hard**	The most strenuous effort you have ever experienced.
**20**	**Maximal exertion**	Maximal heaviness.

Borg RPE Scale®
Ratings (R) of Perceived (P) Exertion (E).
© Gunnar Borg, 1970, 1998, 2017
English

COMPASS-31

Supplemental Appendix 2. Instrument - COMPASS 31

1. In the past year, have you ever felt faint, dizzy, "goofy", or had difficulty thinking soon after standing up from a sitting or lying position?
   1 Yes
   2 No (if you marked No, please skip to question 5)

2. When standing up, how frequently do you get these feelings or symptoms?
   1 Rarely
   2 Occasionally
   3 Frequently
   4 Almost Always

3. How would you rate the severity of these feelings or symptoms?
   1 Mild
   2 Moderate
   3 Severe

4. In the past year, have these feelings or symptoms that you have experienced:
   1 Gotten much worse
   2 Gotten somewhat worse
   3 Stayed about the same
   4 Gotten somewhat better
   5 Gotten much better
   6 Completely gone

5. In the past year, have you ever noticed color changes in your skin, such as red, white, or purple?
   1 Yes
   2 No (if you marked No, please skip to question 8)

6. What parts of your body are affected by these color changes? (Check all that apply)
   1 Hands
   2 Feet

7. Are these changes in your skin color:
   1 Getting much worse
   2 Getting somewhat worse
   3 Staying about the same
   4 Getting somewhat better
   5 Getting much better
   6 Completely gone

8. In the past 5 years, what changes, if any, have occurred in your general body sweating?

1 I sweat much more than I used to
2 I sweat somewhat more than I used to
3 I haven't noticed any changes in my sweating
4 I sweat somewhat less than I used to
5 I sweat much less than I used to

9. Do your eyes feel excessively dry?

1 Yes
2 No

10. Does your mouth feel excessively dry?

1 Yes
2 No

11. For the symptom of dry eyes or dry mouth that you have had for the longest period of time, is this symptom:

1 I have not had any of these symptoms
2 Getting much worse
3 Getting somewhat worse
4 Staying about the same
5 Getting somewhat better
6 Getting much better
7 Completely gone

12. In the past year, have you noticed any changes in how quickly you get full when eating a meal?

1 I get full a lot more quickly now than I used to
2 I get full more quickly now than I used to
3 I haven't noticed any change
4 I get full less quickly now than I used to
5 I get full a lot less quickly now than I used to

13. In the past year, have you felt excessively full or persistently full (bloated feeling) after a meal?

1 Never
2 Sometimes
3 A lot of the time

14. In the past year, have you vomited after a meal?

1 Never
2 Sometimes
3 A lot of the time

15. In the past year, have you had a cramping or colicky abdominal pain?
  1 Never
  2 Sometimes
  3 A lot of the time

16. In the past year, have you had any bouts of diarrhea?
  1 Yes
  2 No (if you marked No, please skip to question 20)

17. How frequently does this occur?
  1 Rarely
  2 Occasionally
  3 Frequently ____________ times per month
  4 Constantly

18. How severe are these bouts of diarrhea?
  1 Mild
  2 Moderate
  3 Severe

19. Are your bouts of diarrhea getting:
  1 Much worse
  2 Somewhat worse
  3 Staying the same
  4 Somewhat better
  5 Much better
  6 Completely gone

20. In the past year, have you been constipated?
  1 Yes
  2 No (if you marked No, please skip to question 24)

21. How frequently are you constipated?
  1 Rarely
  2 Occasionally
  3 Frequently ____________ times per month
  4 Constantly

22. How severe are these episodes of constipation?
  1 Mild
  2 Moderate
  3 Severe

23. Is your constipation getting:
1 Much worse
2 Somewhat worse
3 Staying the same
4 Somewhat better
5 Much better
6 Completely gone

24. In the past year, have you ever lost control of your bladder function?
1 Never
2 Occasionally
3 Frequently ____________ times per month
4 Constantly

25. In the past year, have you had difficulty passing urine?
1 Never
2 Occasionally
3 Frequently ____________ times per month
4 Constantly

26. In the past year, have you had trouble completely emptying your bladder?
1 Never
2 Occasionally
3 Frequently ____________ times per month
4 Constantly

27. In the past year, without sunglasses or tinted glasses, has bright light bothered your eyes?
1 Never (if you marked Never, please skip to question 29)
2 Occasionally
3 Frequently
4 Constantly

28. How severe is this sensitivity to bright light?
1 Mild
2 Moderate
3 Severe

29. In the past year, have you had trouble focusing your eyes?
1 Never (if you marked Never, please skip to question 31)
2 Occasionally
3 Frequently
4 Constantly

30. How severe is this focusing problem?
1 Mild
2 Moderate
3 Severe

31. Is the most troublesome symptom with your eyes (i.e. sensitivity to bright light or trouble focusing) getting:

1 I have not had any of these symptoms
2 Much worse
3 Somewhat worse
4 Staying about the same
5 Somewhat better
6 Much better
7 Completely gone

Aanvullende bijlage 2. Instrument - COMPASS 31

1. Voelde u zich het afgelopen jaar wel eens flauw, duizelig of vreemd of kostte het u wel eens moeite om kort na het opstaan uit zittende of liggende positie helder te denken?
1 Ja
2 Nee (als u Nee hebt geantwoord, kunt u doorgaan naar vraag 5)

2. Hoe vaak hebt u na het opstaan last van deze gevoelens of symptomen?
1 Zelden
2 Soms
3 Vaak
4 Bijna altijd

3. Hoe beoordeelt u de ernst van deze gevoelens of symptomen?
1 Licht
2 Gemiddeld
3 Ernstig

4. Zijn deze gevoelens of symptomen die u hebt ervaren het afgelopen jaar:
1 veel heviger geworden
2 iets heviger geworden
3 ongeveer hetzelfde gebleven
4 iets beter geworden
5 veel beter geworden
6 geheel verdwenen

5. Zijn u in het afgelopen jaar wel eens kleurveranderingen van de huid opgevallen, zoals een rode, witte of paarse kleur?
1 Ja
2 Nee (als u Nee hebt geantwoord, kunt u doorgaan naar vraag 8)

6. Op welke lichaamsdelen hebt u deze kleurveranderingen waargenomen? (Alles aankruisen dat van toepassing is)
1 Handen
2 Voeten

7. Hoe ontwikkelen deze veranderingen in uw huidskleur zich?
1 Ze worden veel heviger
2 Ze worden iets heviger
3 Ze blijven ongeveer hetzelfde
4 Ze worden iets minder hevig
5 Ze worden veel minder hevig
6 Ze zijn geheel verdwenen

8. Is het zweten van uw lichaam de afgelopen 5 jaar over het algemeen veranderd? Zo ja, wat is er veranderd?

1	Ik zweet veel meer dan vroeger
2	Ik zweet iets meer dan vroeger
3	Ik heb geen veranderingen in het zweten opgemerkt
4	Ik zweet iets minder dan vroeger
5	Ik zweet veel minder dan vroeger

9. Voelen uw ogen heel erg droog?

1	Ja
2	Nee

10. Voelt uw mond heel erg droog?

1	Ja
2	Nee

11. Is er wat betreft het symptoom waar u het langst last van hebt (droge ogen of droge mond) iets veranderd?

1	Ik heb deze symptomen niet gehad
2	Ze worden veel heviger
3	Ze worden iets heviger
4	Ze blijven ongeveer hetzelfde
5	Ze worden iets minder hevig
6	Ze worden veel minder hevig
7	Ze zijn geheel verdwenen

12. Is er het afgelopen jaar iets veranderd in hoe snel u vol zit tijdens de maaltijd?

1	Ik zit nu veel sneller vol dan vroeger
2	Ik zit nu sneller vol dan vroeger
3	Ik heb geen verandering opgemerkt
4	Ik zit nu minder snel vol dan vroeger
5	Ik zit nu veel minder snel vol dan vroeger

13. Voelde u zich het afgelopen jaar wel eens ongewoon of aanhoudend vol na de maaltijd (een opgeblazen gevoel)?

1	Nooit
2	Soms
3	Vaak

14. Hebt u het afgelopen jaar wel eens overgegeven na een maaltijd?

1	Nooit
2	Soms
3	Vaak

15. Hebt u het afgelopen jaar wel eens een krampende of koliekachtige buikpijn gehad?
 1 Nooit
 2 Soms
 3 Vaak

16. Hebt u het afgelopen jaar wel eens aanvallen van diarree gehad?
 1 Ja
 2 Nee (als u Nee hebt geantwoord, kunt u doorgaan naar vraag 20)

17. Hoe vaak gebeurde dat?
 1 Zelden
 2 Soms
 3 Vaak ____________ maal per maand
 4 Voortdurend

18. Hoe ernstig zijn die aanvallen van diarree?
 1 Licht
 2 Gemiddeld
 3 Ernstig

19. Worden uw aanvallen van diarree:
 1 Veel erger
 2 Erger
 3 Blijven hetzelfde
 4 Iets beter
 5 Veel beter
 6 Ze zijn geheel verdwenen

20. Hebt u het afgelopen jaar wel eens last gehad van verstopping?
 1 Ja
 2 Nee (als u Nee hebt geantwoord, kunt u doorgaan naar vraag 24)

21. Hoe vaak hebt u last van verstopping?
 1 Zelden
 2 Soms
 3 Vaak ____________ maal per maand
 4 Voortdurend

22. Hoe ernstig zijn die perioden van verstopping?
 1 Licht
 2 Gemiddeld
 3 Ernstig

23. Wordt uw verstopping:

1 Veel erger
2 Iets erger
3 Blijven hetzelfde
4 Iets beter
5 Veel beter
6 Ze zijn geheel verdwenen

24. Hebt u afgelopen jaar wel eens ongewenst urineverlies gehad?

1 Nooit
2 Soms
3 Vaak ____________ maal per maand
4 Voortdurend

25. Hebt u het afgelopen jaar moeite met plassen gehad?

1 Nooit
2 Soms
3 Vaak ____________ maal per maand
4 Voortdurend

26. Hebt u afgelopen jaar wel eens moeite gehad om uw blaas helemaal te legen?

1 Nooit
2 Soms
3 Vaak ____________ maal per maand
4 Voortdurend

27. Had u het afgelopen jaar als u geen zonnebril of donkere bril droeg, last van uw ogen bij fel licht?

1 Nooit (als u Nooit hebt geantwoord, kunt u doorgaan naar vraag 29)
2 Soms
3 Vaak
4 Voortdurend

28. Hoe ernstig is deze gevoeligheid voor fel licht?

1 Licht
2 Matig
3 Ernstig

29. Hebt u afgelopen jaar wel eens moeite gehad om uw ogen te focussen?

1 Nooit (als u Nooit hebt geantwoord, kunt u doorgaan naar vraag 31)
2 Soms
3 Vaak
4 Voortdurend

30. Hoe ernstig is dit probleem met focussen?

1 Licht
2 Matig
3 Ernstig

31. Is er bij de oogsymptomen waar u het meest last van hebt (gevoeligheid voor fel licht of moeite met focussen), iets veranderd?

1 Ik heb deze symptomen niet gehad
2 Veel erger
3 Iets erger
4 Ze blijven ongeveer hetzelfde
5 Iets beter
6 Veel beter
7 Geheel verdwenen

Annexe complémentaire 2. Questionnaire - COMPASS 31

1. Au cours de l'année passée, vous est-il arrivé de vous sentir faible, « bizarre », d'avoir le vertige ou avez-vous eu du mal à réfléchir peu après vous être levé(e) d'une position assise ou couchée ?

1 Oui
2 Non (si vous avez répondu « non », veuillez passer directement à la question 5)

2. Lorsque vous vous mettez debout, à quelle fréquence avez-vous ces sensations ou symptômes ?

1 Rarement
2 Parfois
3 Souvent
4 Presque toujours

3. Comment évalueriez-vous l'intensité de ces sensations ou symptômes ?

1 Légère
2 Modérée
3 Sévère

4. Au cours de l'année passée, les sensations ou symptômes que vous avez eu(e)s se sont-ils/elles :

1 Beaucoup aggravé(e)s ?
2 Un peu aggravé(e)s ?
3 Il n'y a eu à peu près aucun changement.
4 Un peu amélioré(e)s ?
5 Beaucoup amélioré(e)s ?
6 Ils/elles ont complètement disparu.

5. Au cours de l'année passée, avez-vous remarqué des changements de couleur de votre peau (rouge, blanc ou violet) ?

1 Oui
2 Non (si vous avez répondu « non », veuillez passer directement à la question 8)

6. Quelles étaient les parties de votre corps touchées par ces changements de couleur ? (cochez les réponses pertinentes)

1 Mains
2 Pieds

7. Ces changements de couleur de votre peau sont-ils en train de :

1 Beaucoup s'aggraver ?
2 Un peu s'aggraver ?
3 Il n'y a à peu près aucun changement.
4 Un peu s'améliorer ?
5 Beaucoup s'améliorer ?
6 Ils ont complètement disparu.

8. Au cours des 5 dernières années, avez-vous noté des changements concernant la transpiration de votre corps ?

1 Je transpire beaucoup plus qu'avant.
2 Je transpire un peu plus qu'avant.
3 Je n'ai remarqué aucun changement.
4 Je transpire un peu moins qu'avant.
5 Je transpire beaucoup moins qu'avant.

9. Avez-vous la sensation d'avoir les yeux trop secs ?

1 Oui
2 Non

10. Avez-vous la sensation d'avoir la bouche trop sèche ?

1 Oui
2 Non

11. Le symptôme dont vous souffrez depuis le plus longtemps entre la sécheresse des yeux et celle de la bouche est-il en train de :

1 Je n'ai eu aucun de ces symptômes.
2 Beaucoup s'aggraver ?
3 Un peu s'aggraver ?
4 Il n'y a à peu près aucun changement.
5 Un peu s'améliorer ?
6 Beaucoup s'améliorer ?
7 Il a complètement disparu.

12. Au cours de l'année passée, avez-vous remarqué des changements dans la rapidité avec laquelle vous vous sentez rassasié(e) pendant un repas ?

1 Je suis beaucoup plus rapidement rassasiée(e) qu'avant.
2 Je suis plus rapidement rassasiée(e) qu'avant.
3 Je n'ai remarqué aucun changement.
4 Je suis moins rapidement rassasié(e) qu'avant.
5 Je suis beaucoup moins rapidement rassasié(e) qu'avant.

13. Au cours de l'année passée, vous êtes-vous senti(e) excessivement ou constamment rassasié(e) (ballonnements) après un repas ?

1 Jamais
2 Parfois
3 Souvent

14. Au cours de l'année passée, avez-vous vomi après un repas ?

1 Jamais
2 Parfois
3 Souvent

15. Au cours de l'année passée, avez-vous eu des douleurs au ventre comme des crampes ou des coliques ?

1 Jamais
2 Parfois
3 Souvent

16. Au cours de l'année passée, avez-vous eu des épisodes de diarrhée ?

1 Oui
2 Non (si vous avez répondu « non », veuillez passer directement à la question 20)

17. À quelle fréquence avez-vous eu ces diarrhées ?

1 Rarement
2 Parfois
3 Souvent ____________ fois par mois
4 En permanence

18. Quelle est l'intensité de ces épisodes de diarrhée ?

1 Légère
2 Modérée
3 Sévère

19. Vos épisodes de diarrhée sont-ils en train de :

1 Beaucoup s'aggraver ?
2 Un peu s'aggraver ?
3 Il n'y a aucun changement.
4 Un peu s'améliorer ?
5 Beaucoup s'améliorer ?
6 Ils ont complètement disparu.

20. Au cours de l'année passée, avez-vous été constipé(e) ?

1 Oui
2 Non (si vous avez répondu « non », veuillez passer directement à la question 24)

21. À quelle fréquence êtes-vous constipé(e) ?

1 Rarement
2 Parfois
3 Souvent ____________ fois par mois
4 En permanence

22. Quelle est l'intensité de ces épisodes de constipation ?

1 Légère
2 Modérée
3 Sévère

23. Votre constipation est-elle en train de :

1 Beaucoup s'aggraver ?
2 Un peu s'aggraver ?
3 Il n'y a aucun changement.
4 Un peu s'améliorer ?
5 Beaucoup s'améliorer ?
6 Elle a complètement disparu.

24. Au cours de l'année passée, vous est-il arrivé de perdre le contrôle de votre vessie ?

1 Jamais
2 Parfois
3 Souvent _____________ fois par mois
4 En permanence

25. Au cours de l'année passée, avez-vous eu des difficultés à uriner ?

1 Jamais
2 Parfois
3 Souvent _____________ fois par mois
4 En permanence

26. Au cours de l'année passée, avez-vous eu des difficultés à vider complètement votre vessie ?

1 Jamais
2 Parfois
3 Souvent _____________ fois par mois
4 En permanence

27. Au cours de l'année passée, sans lunettes de soleil ni verres teintés, la lumière vive vous a-t-elle gêné(e) ?

1 Jamais (si vous avez répondu « jamais », veuillez passer directement à la question 29)
2 Parfois
3 Souvent
4 En permanence

28. Quelle est l'intensité de cette sensibilité à la lumière vive ?

1 Légère
2 Modérée
3 Sévère

29. Au cours de l'année passée, vos yeux ont-ils eu du mal à accommoder (faire le point) ?

1 Jamais (si vous avez répondu « jamais », veuillez passer directement à la question 31)
2 Parfois
3 Souvent
4 En permanence

30. Quelle est l'intensité de ce problème d'accommodation ?

1 Légère
2 Modérée
3 Sévère

31. Le symptôme qui touche vos yeux et qui vous gêne le plus (c.-à-d. soit la sensibilité à la lumière vive, soit le problème d'accommodation) est-il en train de :

1 Je n'ai eu aucun de ces symptômes.
2 Beaucoup s'aggraver ?
3 Un peu s'aggraver ?
4 Il n'y a à peu près aucun changement.
5 Un peu s'améliorer ?
6 Beaucoup s'améliorer ?
7 Il a complètement disparu.

WAYS Questionnaire

Please provide the following information:

Name:_________________________________ Date:____________________
Month / Day / Year

Identification Number (optional):____________ Gender (Circle): **M** **F** Age:____

Marital Status (check): ❑ Single ❑ Married ❑ Widowed ❑ Separate/Divorced

TO THE COUNSELOR

Fill out your Institutional Address below:

____
Name/ Institution:

____
Address

Instructions

To respond to the statements in this questionnaire, you must have a specific stressful situation in mind. Take a few moments and think about the most stressful situation that you have experienced in the *past week*.

By "stressful" we mean a situation that was difficult or troubling for you, either because you felt distressed about what happened, or because you had to use considerable effort to deal with the situation. The situation may have involved your family, your job, your friends, or something else important to you. Before responding to the statements, think about the details of this stressful situation, such as where it happened, who was involved, how you acted, and why it was important to you. While you may still be involved in the situation, or it could have already happened, it should be the most stressful situation that you experienced during the week.

As you respond to each of the statements, please keep this stressful situation in mind. **Read each statement carefully and indicate, by circling 0, 1, 2 or 3 , to what extent you used it in the situation.**

**Key:**	**0** = Does not apply or not used	**1** = Used somewhat
	**2** = Used quite a bit	**3** = Used a great deal

Please try to respond to every question.

0 = Does not apply or not used   1 = Used somewhat   2 = Used quite a bit   3 = Used a great deal

1.	I just concentrated on what I had to do next – the next step.	**0**	**1**	**2**	**3**
2.	I tried to analyze the problem in order to understand it better.	**0**	**1**	**2**	**3**
3.	I turned to work or another activity to take my mind off things.	**0**	**1**	**2**	**3**
4.	I felt that time would have made a difference – the only thing was to wait.	**0**	**1**	**2**	**3**
5.	I bargained or compromised to get something positive from the situation.	**0**	**1**	**2**	**3**
6.	I did something that I didn't think would work, but at least I was doing something.	**0**	**1**	**2**	**3**
7.	I tried to get the person responsible to change his or her mind.	**0**	**1**	**2**	**3**
8.	I talked to someone to find out more about the situation.	**0**	**1**	**2**	**3**
9.	I criticized or lectured myself.	**0**	**1**	**2**	**3**
10.	I tried not to burn my bridges, but leave things open somewhat.	**0**	**1**	**2**	**3**
11.	I hoped for a miracle.	**0**	**1**	**2**	**3**
12.	I went along with fate; sometimes I just have bad luck.	**0**	**1**	**2**	**3**
13.	I went on as if nothing had happened.	**0**	**1**	**2**	**3**
14.	I tried to keep my feelings to myself.	**0**	**1**	**2**	**3**
15.	I looked for the silver lining, so to speak; I tried to look on the bright side of things.	**0**	**1**	**2**	**3**
16.	I slept more than usual.	**0**	**1**	**2**	**3**
17.	I expressed anger to the person(s) who caused the problem.	**0**	**1**	**2**	**3**
18.	I accepted sympathy and understanding from someone.	**0**	**1**	**2**	**3**
19.	I told myself things that helped me feel better.	**0**	**1**	**2**	**3**
20.	I was inspired to do something creative about the problem.	**0**	**1**	**2**	**3**
21.	I tried to forget the whole thing.	**0**	**1**	**2**	**3**
22.	I got professional help.	**0**	**1**	**2**	**3**

Go on to next page

0 = Does not apply or not used   1 = Used somewhat   2 = Used quite a bit   3 = Used a great deal

23.	I changed or grew as a person.	**0**	**1**	**2**	**3**
24.	I waited to see what would happen before doing anything.	**0**	**1**	**2**	**3**
25.	I apologized or did something to make up.	**0**	**1**	**2**	**3**
26.	I made a plan of action and followed it.	**0**	**1**	**2**	**3**
27.	I accepted the next best thing to what I wanted.	**0**	**1**	**2**	**3**
28.	I let my feelings out somehow.	**0**	**1**	**2**	**3**
29.	I realized that I had brought the problem on myself.	**0**	**1**	**2**	**3**
30.	I came out of the experience better than when I went in.	**0**	**1**	**2**	**3**
31.	I talked to someone who could do something concrete about the problem.	**0**	**1**	**2**	**3**
32.	I tried to get away from it for a while by resting or taking a vacation	**0**	**1**	**2**	**3**
33.	I tried to make myself feel better by eating, drinking, smoking, using drugs, or medications, etc.	**0**	**1**	**2**	**3**
34.	I took a big chance or did something very risky to solve the problem.	**0**	**1**	**2**	**3**
35.	I tried not to act too hastily or follow my first hunch.	**0**	**1**	**2**	**3**
36.	I found new faith.	**0**	**1**	**2**	**3**
37.	I maintained my pride and kept a stiff upper lip.	**0**	**1**	**2**	**3**
38.	I rediscovered what is important in life.	**0**	**1**	**2**	**3**
39.	I changed something so things would turn out all right.	**0**	**1**	**2**	**3**
40.	I generally avoided being with people.	**0**	**1**	**2**	**3**
41.	I didn't let it get to me; I refused to think too much about it.	**0**	**1**	**2**	**3**
42.	I asked advice from a relative or friend I respected.	**0**	**1**	**2**	**3**
43.	I kept others from knowing how bad things were.	**0**	**1**	**2**	**3**
44.	I made light of the situation; I refused to get too serious about it.	**0**	**1**	**2**	**3**

Go on to next page

0 = Does not apply or not used 1 = Used somewhat 2 = Used quite a bit 3 = Used a great deal

45. I talked to someone about how I was feeling.	**0**	**1**	**2**	**3**
46. I stood my ground and fought for what I wanted.	**0**	**1**	**2**	**3**
47. I took it out on other people.	**0**	**1**	**2**	**3**
48. I drew on my past experiences; I was in a similar situation before.	**0**	**1**	**2**	**3**
49. I knew what had to be done, so I doubled my efforts to make things work.	**0**	**1**	**2**	**3**
50. I refused to believe that it had happened	**0**	**1**	**2**	**3**
51. I promised myself that things would be different next time.	**0**	**1**	**2**	**3**
52. I came up with a couple of different solutions to the problem.	**0**	**1**	**2**	**3**
53. I accepted the situation, since nothing could be done.	**0**	**1**	**2**	**3**
54. I tried to keep my feeling about the problem from interfering with other things.	**0**	**1**	**2**	**3**
55. I wished that I could change what had happened or how I felt.	**0**	**1**	**2**	**3**
56. I changed something about myself.	**0**	**1**	**2**	**3**
57. I daydreamed or imagined a better time or place than the one I was in.	**0**	**1**	**2**	**3**
58. I wished that the situation would go away or somehow be over with.	**0**	**1**	**2**	**3**
59. I had fantasies or wishes about how things might turn out.	**0**	**1**	**2**	**3**
60. I prayed.	**0**	**1**	**2**	**3**
61. I prepared myself for the worst.	**0**	**1**	**2**	**3**
62. I went over in my mind what I would say or do.	**0**	**1**	**2**	**3**
63. I thought about how a person I admire would handle this situation and used that as a model.	**0**	**1**	**2**	**3**
64 I tried to see things from the other person's point of view.	**0**	**1**	**2**	**3**
65. I reminded myself how much worse things could be.	**0**	**1**	**2**	**3**
66. I jogged or exercised.	**0**	**1**	**2**	**3**

Stop Here.

Vult u alstublieft de volgende gegevens in:

Naam: ___________________________________ Datum: ____________________
Dag / Maand / Jaar

Identificatienummer (optioneel): ___________ Geslacht (omcirkel): **M** **V** Leeftijd: __

Burgerlijke staat (kruis aan): ❑ Vrijgezel ❑ Gehuwd ❑ Weduwe/weduwnaar ❑ Gescheiden

VOOR DE ONDERZOEKER

Vul hieronder het adres van uw instelling in:

Naam/ Instelling:

Adres:

Instructies

U dient zich, bij het reageren op de uitspraken in deze vragenlijst, een specifieke stressvolle situatie voor de geest te halen. Neemt u even de tijd om na te gaan wat de *afgelopen 7 dagen* de meest stressvolle situatie voor u.

Met 'stressvol' bedoelen we een situatie die moeilijk of lastig voor u was, hetzij omdat u overstuur raakte over wat er gebeurde, hetzij omdat het u behoorlijk wat moeite kostte om met de situatie om te gaan. Deze situatie kan betrekking hebben op uw gezin, uw werk, uw vrienden of iets anders dat belangrijk voor u is. Neemt u, voordat u reageert op de uitspraken, de stressvolle situatie heel precies in gedachten, en denk bijvoorbeeld aan waar het gebeurde, wie er bij betrokken was, wat u deed, en waarom het belangrijk voor u was. Ongeacht of u zich nog steeds in de stressvolle situatie bevindt, of dat het al achter de rug is, het dient te gaan om de meest stressvolle situatie die u de afgelopen 7 dagen heeft meegemaakt.

Houd bij het reageren op elk van de uitspraken deze stressvolle situatie steeds in gedachten. **Lees elke uitspraak aandachtig, en geef vervolgens aan in hoeverre u er in de situatie gebruik van heeft gemaakt door een 0, 1, 2 of een 3 te omcirkelen.**

Antwoordcodes: **0 =** Niet van toepassing of niet gebruikt **1 =** Een beetje gebruikt
2 = Behoorlijk veel gebruikt **3 =** Heel veel gebruikt

Probeert u alstublieft bij elke uitspraak een antwoord te geven.

0 = Niet van toepassing of niet gebruikt   1 = Een beetje gebruikt   2 = Behoorlijk veel gebruikt   3 = Heel veel gebruikt

1.	Ik heb mij gewoon gericht op wat ik vervolgens moest doen – de volgende stap	0	1	2	3
2.	Ik heb geprobeerd het probleem te analyseren om het beter te begrijpen	0	1	2	3
3.	Ik heb me ter afleiding op mijn werk of andere activiteiten gericht	0	1	2	3
4.	Ik heb gedacht dat het wel weer over zou gaan – gewoon een kwestie van een beetje geduld	0	1	2	3
5.	Ik heb onderhandeld of compromissen gesloten om iets positiefs uit de situatie te kunnen halen	0	1	2	3
6.	Ik heb iets gedaan waarvan ik niet verwachtte dat het iets zou uithalen, maar ik was tenminste iets aan het doen	0	1	2	3
7.	Ik heb geprobeerd om degene die verantwoordelijk was op andere	0	1	2	3
8.	Ik heb met iemand gepraat om meer over de situatie te weten te komen	0	1	2	3
9.	Ik heb mezelf bekritiseerd of de les gelezen	0	1	2	3
10.	Ik heb geprobeerd om geen beslissingen te nemen waar ik niet op kon terugkomen, maar de dingen een beetje open te laten	0	1	2	3
11.	Ik heb gehoopt op een wonder	0	1	2	3
12.	Ik heb me in mijn lot geschikt; soms heb ik gewoon pech	0	1	2	3
13.	Ik ben verder gegaan alsof er niets gebeurd was	0	1	2	3
14.	Ik heb geprobeerd mijn gevoelens voor me te houden	0	1	2	3
15.	Ik heb geprobeerd om het van de positieve kant te bekijken	0	1	2	3
16.	Ik heb meer geslapen dan gewoonlijk	0	1	2	3
17.	Ik heb mijn boosheid geuit jegens de perso(o)n(en) die het probleem veroorzaakt had(den)	0	1	2	3
18.	Ik heb het medeleven en het begrip van iemand geaccepteerd	0	1	2	3
19.	Ik heb mijzelf dingen verteld waardoor ik me beter voelde	0	1	2	3
20.	Ik ben ertoe bewogen om iets creatiefs aan het probleem te doen	0	1	2	3
21.	Ik heb geprobeerd om de hele toestand van me af te zetten	0	1	2	3
22.	Ik heb professionele hulp gekregen	0	1	2	3

Ga naar de volgende pagina

0 = Niet van toepassing of niet gebruikt 1 = Een beetje gebruikt 2 = Behoorlijk veel gebruikt 3 = Heel veel gebruikt

23. Ik ben veranderd of gegroeid als mens	0	1	2	3
24. Ik heb eerst de kat uit de boom gekeken voordat ik iets deed	0	1	2	3
25. Ik heb mijn excuses aangeboden of iets gedaan om het weer goed te maken	0	1	2	3
26. Ik heb een actieplan gemaakt en heb dit uitgevoerd	0	1	2	3
27. Ik heb genoegen genomen met de tweede keus	0	1	2	3
28. Ik heb op de een of andere manier lucht gegeven aan mijn gevoelens	0	1	2	3
29. Ik heb me gerealiseerd dat ik het probleem zelf over me had afgeroepen	0	1	2	3
30. Ik ben sterker uit de situatie gekomen dan dat ik erin ben gegaan	0	1	2	3
31. Ik heb met iemand gepraat die iets concreets kon doen aan het probleem	0	1	2	3
32. Ik heb geprobeerd het even achter me te laten door uit te rusten of vakantie te nemen	0	1	2	3
33. Ik heb geprobeerd mijzelf beter te laten voelen met eten, drinken, roken, verdovende middelen, medicijngebruik, etc.	0	1	2	3
34. Ik heb een groot risico genomen of iets zeer riskants gedaan om het probleem op te lossen	0	1	2	3
35. Ik heb geprobeerd niet overhaast te handelen of meteen op mijn intuïtie af te gaan.	0	1	2	3
36. Ik heb me gewend tot godsdienst of spiritualiteit	0	1	2	3
37. Ik heb de eer aan mijzelf gehouden en mijn gevoelens niet laten zien	0	1	2	3
38. Ik heb weer ontdekt wat belangrijk is in het leven	0	1	2	3
39. Ik heb iets veranderd zodat het goed af zou lopen	0	1	2	3
40. Ik heb over het algemeen vermeden om met mensen samen te zijn	0	1	2	3
41. Ik heb ervoor gezorgd dat het mij niet zou raken; ik weigerde er te veel over na te denken	0	1	2	3
42. Ik heb advies gevraagd aan een familielid of vriend die ik waardeer	0	1	2	3
43. Ik heb ervoor gezorgd dat anderen niet wisten hoe erg de situatie was	0	1	2	3
44. Ik heb de situatie gebagatelliseerd; ik heb geweigerd het te serieus te nemen	0	1	2	3

Ga naar de volgende pagina

0 = Niet van toepassing of niet gebruikt 1 = Een beetje gebruikt 2 = Behoorlijk veel gebruikt 3 = Heel veel gebruikt

45. Ik heb met iemand gesproken over hoe ik mij voelde	0	1	2	3
46. Ik heb voet bij stuk gehouden en gevochten voor wat ik wilde	0	1	2	3
47. Ik heb me op anderen afgereageerd	0	1	2	3
48. Ik heb gebruik gemaakt van eerdere ervaringen; ik heb een dergelijke situatie eerder meegemaakt	0	1	2	3
49. Ik wist wat gedaan moest worden, dus ik heb extra mijn best gedaan om te zorgen dat het goed kwam	0	1	2	3
50. Ik heb geweigerd te geloven dat het was gebeurd	0	1	2	3
51. Ik heb mij voorgenomen dat het de volgende keer anders zal gaan	0	1	2	3
52. Ik heb een aantal oplossingen bedacht voor het probleem	0	1	2	3
53. Ik heb de situatie geaccepteerd, want er viel toch niets aan te doen	0	1	2	3
54. Ik heb geprobeerd om mijn gevoelens over het probleem niet van invloed te laten zijn op andere dingen	0	1	2	3
55. Ik heb gewenst dat ik kon veranderen wat er was gebeurd of hoe ik mij voelde	0	1	2	3
56. Ik heb iets bij mijzelf veranderd	0	1	2	3
57. Ik heb ervan gedroomd of me voorgesteld dat ik me op een betere tijd of plaats bevond	0	1	2	3
58. Ik heb gewenst dat de situatie voorbij zou gaan of dat er op de een of andere manier een einde aan zou komen	0	1	2	3
59. Ik heb fantasieën of wensen gehad over hoe een en ander zou kunnen aflopen	0	1	2	3
60. Ik heb gebeden	0	1	2	3
61. Ik heb mijzelf op het ergste voorbereid	0	1	2	3
62. Ik heb mij bedacht wat ik zou zeggen of doen	0	1	2	3
63. Ik heb gedacht hoe iemand die ik bewonder met deze situatie om zou gaan en heb me daar aan gespiegeld	0	1	2	3
64. Ik heb geprobeerd om de dingen vanuit het standpunt van de ander te zien	0	1	2	3
65. Ik heb mezelf eraan herinnerd hoeveel erger het nog had kunnen zijn	0	1	2	3
66. Ik ben gaan hardlopen of sporten	0	1	2	3

Stop hier.

Merci de nous fournir les informations suivantes :

Nom :________________________________ Date : _____________________
jour / mois / année

Numéro d'identification (facultatif) :_____ Sexe (entourez) : **H** **F** Age : ___

Situation familiale (cochez) : ❑ Célibataire ❑ Marié(e) ❑ Veuf(ve) ❑ Séparé(e) / Divorcé(e)

A REMPLIR PAR LE CONSEILLER

Veuillez inscrire l'adresse de votre établissement ci-dessous :

Nom / Etablissement :

Adresse

Instructions

Par « stressante », nous voulons dire que cette situation a été difficile ou vous a posé problème, soit parce que vous vous êtes senti(e) touché(e) par les événements, soit parce que vous avez dû faire des efforts importants pour gérer la situation. Cette situation a pu concerner votre famille, votre travail, vos amis ou toute autre chose importante pour vous. Avant de répondre aux questions, pensez aux détails de cette situation stressante, par exemple à l'endroit où elle s'est passée, aux personnes concernées, à la manière dont vous avez agi et aux raisons pour lesquelles cette situation était importante pour vous. Peu importe si cette situation vous pose encore problème à l'heure actuelle ou si elle appartient au passé : il doit s'agir de la situation la plus stressante que vous avez vécue au cours des 7 derniers jours.

En répondant à chacune des questions suivantes, gardez bien à l'esprit cette situation stressante. **Lisez chacune des affirmations avec attention et indiquez dans quelle mesure vous avez adopté ce comportement pour gérer cette situation en entourant 0, 1, 2 ou 3.**

Réponses : 0 = Non concerné(e) ou pas du tout **1 =** Un peu
2 = Beaucoup **3 =** Enormément

Merci d'essayer de répondre à toutes les questions.

1. Je me suis concentré(e) sur ce que j'avais à faire ensuite, sur l'étape suivante………………	0 1 2 3
2. J'ai essayé d'analyser le problème pour mieux le comprendre………………………………….	0 1 2 3
3. Je me suis tourné(e) vers le travail ou vers une autre activité pour me changer les idées ….	0 1 2 3
4. J'ai pensé que les choses changeraient avec le temps, et que la seule chose à faire était d'attendre………………………………………………………………………………………	0 1 2 3
5. J'ai négocié ou accepté un compromis afin de tirer quelque chose de positif de cette situation	0 1 2 3
6. J'ai fait quelque chose tout en sachant que ça ne marcherait pas, mais au moins je ne suis pas resté(e) les bras croisés………………………………………………………………………	0 1 2 3
7. J'ai essayé de faire changer d'avis la personne responsable……………………………………..	0 1 2 3
8. J'ai parlé à quelqu'un pour en savoir plus sur cette situation .... …………….	0 1 2 3
9. J'ai fait mon autocritique ou je me suis fait des reproches .... ……………..	0 1 2 3
10. J'ai essayé de ne pas brûler toutes mes cartouches et de garder une marge de manœuvre…	0 1 2 3
11. J'ai espéré un miracle………………………………………………………………………………	0 1 2 3
12. Je me suis résigné(e) ; parfois je n'ai tout simplement pas de chance…………………………..	0 1 2 3
13. J'ai continué comme si de rien n'était……………………………………………………………..	0 1 2 3
14. J'ai essayé de garder mes sentiments pour moi………………………………………………….	0 1 2 3
15. J'ai cherché l'aspect positif de la situation ; j'ai essayé de voir le bon côté des choses……….	0 1 2 3
16. J'ai dormi plus que d'habitude…………………………………………………………………….	0 1 2 3
17. Je me suis mis(e) en colère contre la(les) personne(s) responsable(s) du problème…………	0 1 2 3
18. J'ai accepté la sympathie et la compréhension qu'on me témoignait……………………………	0 1 2 3
19. Je me suis dit des choses qui m'ont réconforté(e)…………………………………………………	0 1 2 3
20. J'ai été poussé(e) à faire preuve d'inventivité pour gérer le problème…………………………..	0 1 2 3
21. J'ai essayé d'oublier cette situation stressante…………………………………………………….	0 1 2 3
22. J'ai consulté un spécialiste... ………………	0 1 2 3

Veuillez passer à la page suivante

WAYS - French

23. J'ai changé ou mûri en tant qu'individu.. **0 1 2 3**

24. J'ai attendu de voir ce qui se passerait avant de faire quoi que ce soit.............................. **0 1 2 3**

25. Je me suis excusé(e) ou j'ai fait quelque chose pour me rattraper...................................... **0 1 2 3**

26. J'ai mis au point un plan d'action et je l'ai suivi.. **0 1 2 3**

27. J'ai accepté quelque chose d'un peu moins bien que ce que je voulais.............................. **0 1 2 3**

28. J'ai exprimé mes sentiments tant bien que mal.. **0 1 2 3**

29. Je me suis rendu(e) compte que c'était moi qui avais provoqué le problème........................ **0 1 2 3**

30. Je suis ressorti(e) plus fort(e) de cette expérience **0 1 2 3**

31. J'ai parlé à quelqu'un qui pouvait agir concrètement sur le problème................................. **0 1 2 3**

32. J'ai essayé de m'éloigner du problème pendant un moment en me reposant ou en prenant des vacances.. **0 1 2 3**

33. J'ai essayé de me réconforter en mangeant, en buvant, en fumant, en prenant des drogues ou des médicaments, etc... **0 1 2 3**

34. J'ai pris un gros risque ou j'ai fait quelque chose de très risqué pour résoudre le problème....... **0 1 2 3**

35. J'ai essayé de ne pas agir trop précipitamment ou de ne pas suivre ma première intuition........ **0 1 2 3**

36. Je me suis tourné(e) vers la religion ou la spiritualité... **0 1 2 3**

37. J'ai gardé la tête haute et je n'ai pas montré mes sentiments.. **0 1 2 3**

38. J'ai redécouvert ce qui est important dans la vie... **0 1 2 3**

39. J'ai changé quelque chose pour faire en sorte que tout s'arrange....................................... **0 1 2 3**

40. En général, j'ai évité la compagnie d'autres personnes.. **0 1 2 3**

41. Je ne me suis pas laissé(e) atteindre par la situation; j'ai refusé de trop y penser.................... **0 1 2 3**

42. J'ai demandé conseil à un parent ou à un(e) ami(e) que je respecte.................................... **0 1 2 3**

43. J'ai fait en sorte que les autres ne connaissent pas la gravité de la situation.......................... **0 1 2 3**

44. J'ai dédramatisé la situation, j'ai refusé de la prendre trop au sérieux **0 1 2 3**

Veuillez passer à la page suivante

WAYS - French

45. J'ai parlé à quelqu'un de ce que je ressentais... 0 1 2 3

46. J'ai tenu bon et je me suis battu(e) pour ce que je voulais.. 0 1 2 3

47. Je m'en suis pris(e) aux autres.. 0 1 2 3

48. Je me suis servi(e) de mes expériences précédentes ; j'ai connu une situation similaire dans le passé.. 0 1 2 3

49. Je savais ce qu'il fallait faire alors j'ai redoublé d'efforts pour que ça marche....................... 0 1 2 3

50. J'ai refusé de croire à la réalité de cette situation.. 0 1 2 3

51. Je me suis promis que les choses se passeraient autrement la prochaine fois...................... 0 1 2 3

52. J'ai envisagé plusieurs solutions différentes au problème 0 1 2 3

53. J'ai accepté la situation puisqu'il n'y avait rien à faire.. 0 1 2 3

54. J'ai essayé de faire en sorte que mes sentiments sur le problème ne rejaillissent pas sur les autres situations.. 0 1 2 3

55. J'aurais bien aimé pouvoir changer ce qui s'était produit ou ce que je ressentais................. 0 1 2 3

56. J'ai changé quelque chose en moi 0 1 2 3

57. J'ai imaginé que je me retrouvais dans un autre lieu et à un autre moment, dans une situation moins stressante.. 0 1 2 3

58. J'ai souhaité que cette situation disparaisse ou se termine d'une manière ou d'une autre........ 0 1 2 3

59. Je me suis mis(e) à imaginer un dénouement idéal à la situation... 0 1 2 3

60. J'ai prié.. 0 1 2 3

61. Je me suis préparé(e) au pire... 0 1 2 3

62. J'ai répété ce que j'allais dire ou faire.. 0 1 2 3

63. J'ai pensé à la manière dont une personne que j'admire gérerait une telle situation, et j'ai suivi ce modèle 0 1 2 3

64. J'ai essayé de voir les choses du point de vue de l'autre... 0 1 2 3

65. Je me suis dit que les choses pourraient être bien pires... 0 1 2 3

66. J'ai fait du jogging ou de l'exercice physique... 0 1 2 3

Fin du questionnaire.
WAYS - French

Voedingsdagboek

Gedurende 3 dagen (bij voorkeur 2 weekdagen & 1 weekenddag) hou je in dit voedingsdagboek bij wat je op een dag consumeert. Probeer zo volledig mogelijk te zijn door ***de hoeveelheden (gram)*** *en de* ***bereidingswijze*** *te noteren. Vermeld indien mogelijk ook de* ***naam/merk*** *van het product. Voeg gerust ook foto's toe. (Zie voorbeeld onderaan)*

DAG 1 Datum:........................

	**Portie** (gram / aantal stuks / eetlepels)/ **Merk**/ **Bereidingswijze**
**ONTBIJT**<br>*Brood/ontbijtgranen/fruit*<br>*Smeerstof*<br>*Zoet beleg (confituur, choco…)*<br>*Hartig beleg (kaas, charcuterie)*<br>*Melkproducten*<br>*Drank*<br>*(water,koffie,thee,fruitsap,…)*	
**TUSSENDOOR**<br>*Fruit,yoghurt,koekjes,*<br>*chocolade,chips,…*	
**MIDDAG**<br>*Brood /beleg /smeerstof*<br>*Soep*<br>*Vlees/vis/vegetarisch*<br>*Groenten*<br>*Aardappelen/rijst/pasta*<br>*Bereidingsvet*<br>*Sauzen*<br>*Dessert*<br>*Drank*<br>*(water,koffie,thee,wijn,bier,…)*	
**TUSSENDOOR**<br>*Fruit,yoghurt,koekjes,*<br>*chocolade,chips,…*	
**AVOND**<br>*Brood /beleg /smeerstof*<br>*Soep*<br>*Vlees/vis/vegetarisch*<br>*Groenten*<br>*Aardappelen/rijst/pasta*<br>*Bereidingsvet*<br>*Sauzen*<br>*Dessert*<br>*Drank*<br>*(water,koffie,thee,wijn,bier,…)*	
**LATE SNACK**<br>*Fruit,yoghurt,koekjes,*<br>*chocolade,chips,…*	

DRANKEN

Waterglazen ....

Frisdrank/fruitsap ....

Alcohol ....

Tas koffie/thee ....

BEWEGING

Activiteit:

Duur:

Frequentie:
(per dag/ per week)

DAG 2

Datum:........................

	**Portie** (gram / aantal stuks / eetlepels)/ **Merk**/ **Bereidingswijze**
**ONTBIJT**<br>*Brood/ontbijtgranen/fruit*<br>*Smeerstof*<br>*Zoet beleg (confituur, choco…)*<br>*Hartig beleg (kaas, charcuterie)*<br>*Melkproducten*<br>*Drank*<br>*(water,koffie,thee,fruitsap,…)*	
**TUSSENDOOR**<br>*Fruit,yoghurt,koekjes,*<br>*chocolade,chips,…*	
**MIDDAG**<br>*Brood /beleg /smeerstof*<br>*Soep*<br>*Vlees/vis/vegetarisch*<br>*Groenten*<br>*Aardappelen/rijst/pasta*<br>*Bereidingsvet*<br>*Sauzen*<br>*Dessert*<br>*Drank*<br>*(water,koffie,thee,wijn,bier,…)*	
**TUSSENDOOR**<br>*Fruit,yoghurt,koekjes,*<br>*chocolade,chips,…*	
**AVOND**<br>*Brood /beleg /smeerstof*<br>*Soep*<br>*Vlees/vis/vegetarisch*<br>*Groenten*<br>*Aardappelen/rijst/pasta*<br>*Bereidingsvet*<br>*Sauzen*<br>*Dessert*<br>*Drank*<br>*(water,koffie,thee,wijn,bier,…)*	
**LATE SNACK**<br>*Fruit,yoghurt,koekjes,*<br>*chocolade,chips,…*	

DRANKEN

Waterglazen ....

Frisdrank/fruitsap ....

Alcohol ....

Tas koffie/thee ....

BEWEGING

Activiteit:

Duur:

Frequentie:
(per dag/ per week)

DAG 3

Datum:......................

	Portie (gram / aantal stuks / eetlepels)/ Merk/ Bereidingswijze
**ONTBIJT**<br>*Brood/ontbijtgranen/fruit*<br>*Smeerstof*<br>*Zoet beleg (confituur, choco…)*<br>*Hartig beleg (kaas, charcuterie)*<br>*Melkproducten*<br>*Drank*<br>*(water,koffie,thee,fruitsap,…)*	
**TUSSENDOOR**<br>*Fruit,yoghurt,koekjes,*<br>*chocolade,chips,…*	
**MIDDAG**<br>*Brood /beleg /smeerstof*<br>*Soep*<br>*Vlees/vis/vegetarisch*<br>*Groenten*<br>*Aardappelen/rijst/pasta*<br>*Bereidingsvet*<br>*Sauzen*<br>*Dessert*<br>*Drank*<br>*(water,koffie,thee,wijn,bier,…)*	
**TUSSENDOOR**<br>*Fruit,yoghurt,koekjes,*<br>*chocolade,chips,…*	
**AVOND**<br>*Brood /beleg /smeerstof*<br>*Soep*<br>*Vlees/vis/vegetarisch*<br>*Groenten*<br>*Aardappelen/rijst/pasta*<br>*Bereidingsvet*<br>*Sauzen*<br>*Dessert*<br>*Drank*<br>*(water,koffie,thee,wijn,bier,…)*	
**LATE SNACK**<br>*Fruit,yoghurt,koekjes,*<br>*chocolade,chips,…*	

DRANKEN

Waterglazen ....

Frisdrank/fruitsap ....

Alcohol ....

Tas koffie/thee ....

BEWEGING

Activiteit:

Duur:

Frequentie:
(per dag/ per week)

VOORBEELD

Datum: 31 maart 2022

	Portie (gram / aantal stuks / eetlepels)/ Merk/ Bereidingswijze
**ONTBIJT**<br>*Brood/ontbijtgranen/fruit*<br>*Smeerstof*<br>*Zoet beleg (confituur, choco…)*<br>*Hartig beleg (kaas, charcuterie)*<br>*Melkproducten*<br>*Drank*<br>*(water,koffie,thee,fruitsap,…)*	○ 50 gr havermout + 250 ml ongezoete amandel melk (alpro) + 1 theelepel stevia + 1 handvol blauwe bessen & frambozen<br>○ 1 tas koffie (125 mL) + 2 koffielepels magere melk<br>○ 1 grijze boterham, 1 mespuntje margarine, 1 sneetje gouda kaas
**TUSSENDOOR**<br>*Fruit,yoghurt,koekjes, chocolade,chips,…*	○ 1 x Kiwi<br>○ 1 x volle yoghurt (Danone)
**MIDDAG**<br>*Brood /beleg /smeerstof*<br>*Soep*<br>*Vlees/vis/vegetarisch*<br>*Groenten*<br>*Aardappelen/rijst/pasta*<br>*Bereidingsvet*<br>*Sauzen*<br>*Dessert*<br>*Drank*<br>*(water,koffie,thee,wijn,bier,…)*	○ 3 grijze boterhammen + 1 mespunt minarine + 3 sneetjes kippenwit met kruiden (Delhaize)<br>○ 2 melksandwiches + 3 mespunten chocopasta (Nutella)<br>○ 3 tassen thee (125 mL) + 1 eetlepel honing + 1 wolkje melk
**TUSSENDOOR**<br>*Fruit,yoghurt,koekjes, chocolade,chips,…*	○ 1 taartpunt Cheesecake met speculoos (Tarte de Françoise)
**AVOND**<br>*Brood /beleg /smeerstof*<br>*Soep*<br>*Vlees/vis/vegetarisch*<br>*Groenten*<br>*Aardappelen/rijst/pasta*<br>*Bereidingsvet*<br>*Sauzen*<br>*Dessert*<br>*Drank*<br>*(water,koffie,thee,wijn,bier,…)*	○ 1 grote tas zelfgemaakte courgettensoep (250 mL)<br>○ 125 gram gegrilde steak (zonder vetstof) + 300 gram gestoomde broccoli + 100 gram gebakken krielaardappelen met 5 g boter(solo)<br>○ 3 eetlepels Bearnaisesaus (kant en klaar Delhaize)<br>○ 2 glazen rode wijn
**LATE SNACK**<br>*Fruit,yoghurt,koekjes, chocolade,chips,…*	○ 1 handvol chips (merk: Lays Peper & Zout)<br>○ 30 gram borrelnootjes paprika (merk: Duyvis)<br>○ 3 oreo koekjes

DRANKEN

Waterglazen	8 glazen water
Alcohol	2 glazen wijn
Tas koffie/thee	1 x koffie/ 3 x thee

BEWEGING

Activiteit: Wandelen

Duur: 30 minuten

Frequentie: 4 keer per week
(per dag/ per week)

Journal Alimentaire

Pendant 3 jours (de préférence 2 jours de semaine et 1 jour de week-end), vous gardez un journal de ce que vous consommez en une journée dans ce journal alimentaire. Essayez d'être le plus complet possible en notant les quantités (grammes) et le mode de préparation. Si possible, indiquez également le nom/la marque du produit.
N'hésitez pas également à ajouter des photos. (Voir exemple ci-dessous)

JOUR 1 Date:.........................

	**Portion** (grammes/ nombre de pièces/ cuillères) **/ Marque / Méthode de préparation**
**PETIT-DEJEUNER**<br>*Pain/céréales/matière grasse*<br>*Garnitures sucrées (choco, confiture)*<br>*Garnitures salées (fromage, charcuterie)*<br>*Produits laitiers*<br>*Boisson (Eau, café, thé, jus de fruits)*	
**COLLATION**<br>*Fruits, yaourt, biscuits, chocolat, chips, ...*	
**MIDI**<br>*Pain/céréales/matière grasse*<br>*Soupe*<br>*Viande/poisson/végétarien*<br>*Légumes*<br>*Pommes de terre/riz/pâtes*<br>*Graisse de cuisson*<br>*Sauce*<br>*Dessert*<br>*Boisson (Eau, café, thé, vin, bière)*	
**COLLATION**<br>*Fruits, yaourt, biscuits, chocolat, chips, ...*	
**SOIR**<br>*Pain/céréales/matière grasse*<br>*Soupe*<br>*Viande/poisson/végétarien*<br>*Légumes*<br>*Pommes de terre/riz/pâtes*<br>*Graisse de cuisson*<br>*Dessert*<br>*Boisson (Eau, café, thé, vin, bière)*	
**SNACK**<br>*Fruits, yaourt, biscuits, chocolat, chips, ...*	

BOISSONS

- Verre d'eau ....
- Soda/jus de fruits ....
- L'alcool ....
- Tasse café/thé ....

MOUVEMENT

Activité:

Durée:

Fréquence:
(par jour/par semaine)

JOUR 2

Date:.........................

	**Portion** (grammes/ nombre de pièces/ cuillères) / **Marque** / **Méthode de préparation**
**PETIT-DEJEUNER**<br>*Pain/céréales/matière grasse*<br>*Garnitures sucrées (choco, confiture)*<br>*Garnitures salées (fromage, charcuterie)*<br>*Produits laitiers*<br>*Boisson*<br>*(Eau, café, thé, jus de fruits)*	
**COLLATION**<br>*Fruits, yaourt, biscuits, chocolat, chips, ...*	
**MIDI**<br>*Pain/céréales/ matière grasse*<br>*Soupe*<br>*Viande/poisson/végétarien*<br>*Légumes*<br>*Pommes de terre/riz/pâtes*<br>*Graisse de cuisson*<br>*Sauce*<br>*Dessert*<br>*Boisson*<br>*(Eau, café, thé, vin, bière, …)*	
**COLLATION**<br>*Fruits, yaourt, biscuits, chocolat, chips, ...*	
**SOIR**<br>*Pain/céréales/matière grasse*<br>*Soupe*<br>*Viande/poisson/végétarien*<br>*Légumes*<br>*Pommes de terre/riz/pâtes*<br>*Graisse de cuisson*<br>*Dessert*<br>*Boisson*<br>*(Eau, café, thé, vin, bière)*	
**SNACK**<br>*Fruits, yaourt, biscuits, chocolat, chips, ...*	

BOISSONS

Verre d'eau ....

Soda/jus de fruits ....

L'alcool ....

Tasse café/thé ....

MOUVEMENT

Activité:

Durée:

Fréquence:

(par jour/par semaine)

Jour 3

Date:........................

	**Portion** (grammes/ nombre de pièces/ cuillères) **/ Marque / Méthode de préparation**
**PETIT-DEJEUNER**<br>*Pain/céréales/matière grasse*<br>*Garnitures sucrées (choco, confiture)*<br>*Garnitures salées (fromage, charcuterie)*<br>*Produits laitiers*<br>*Boisson*<br>*(Eau, café, thé, jus de fruits)*	
**COLLATION**<br>*Fruits, yaourt, biscuits, chocolat, chips, ...*	
**MIDI**<br>*Pain/céréales/ matière grasse*<br>*Soupe*<br>*Viande/poisson/végétarien*<br>*Légumes*<br>*Pommes de terre/riz/pâtes*<br>*Graisse de cuisson*<br>*Sauce*<br>*Dessert*<br>*Boisson*<br>*(Eau, café, thé, vin, bière, …)*	
**COLLATION**<br>*Fruits, yaourt, biscuits, chocolat, chips, ...*	
**SOIR**<br>*Pain/céréales/matière grasse*<br>*Soupe*<br>*Viande/poisson/végétarien*<br>*Légumes*<br>*Pommes de terre/riz/pâtes*<br>*Graisse de cuisson*<br>*Dessert*<br>*Boisson*<br>*(Eau, café, thé, vin, bière)*	
**SNACK**<br>*Fruits, yaourt, biscuits, chocolat, chips, ...*	

BOISSONS

Verre d'eau ....

Soda/jus de fruits ....

L'alcool ....

Tasse café/thé ....

MOUVEMENT

Activité:

Durée:

Fréquence:

(par jour/par semaine)

Example

Date: 31 mars 2022

	**Portion** (grammes/ nombre de pièces/ cuillères) / **Marque** / **Méthode de préparation**
**PETIT-DEJEUNER**<br>*Pain/céréales/matière grasse*<br>*Garnitures sucrées (choco, confiture)*<br>*Garnitures salées (fromage, charcuterie)*<br>*Produits laitiers*<br>*Boisson*<br>*(Eau, café, thé, jus de fruits)*	o 50 gr de flocons d'avoine + 250 ml de lait d'amande non sucré (alpro) + 1 cuillère à café de stévia + 1 poignée de myrtilles & framboises<br>o 1 tasse de café (125 ml) + 2 cuillères à café de lait écrémé<br>o 1 tranche de pain gris, 1 pincée de margarine, 1 tranche de gouda
**COLLATION**<br>*Fruits, yaourt, biscuits, chocolat, chips, ...*	o 1 x Kiwi<br>o 1 x yaourt entier (Danone)
**MIDI**<br>*Pain/céréales/ matière grasse*<br>*Soupe*<br>*Viande/poisson/végétarien*<br>*Légumes*<br>*Pommes de terre/riz/pâtes*<br>*Graisse de cuisson*<br>*Sauce*<br>*Dessert*<br>*Boisson*<br>*(Eau, café, thé, vin, bière, …)*	o 3 tranches de pain gris + 1 pincée de minarine + 3 tranches de blanc de poulet aux herbes (Delhaize)<br>o 2 sandwichs au lait + 3 pincées de pâte à tartiner au chocolat (Nutella)<br>o 3 tasses de thé (125 ml) + 1 cuillère à soupe de miel + 1 cuillière de lait
**COLLATION**<br>*Fruits, yaourt, biscuits, chocolat, chips, ...*	o 1 tranche de Cheesecake au spéculoos (Tarte de Françoise)
**SOIR**<br>*Pain/céréales/matière grasse*<br>*Soupe*<br>*Viande/poisson/végétarien*<br>*Légumes*<br>*Pommes de terre/riz/pâtes*<br>*Graisse de cuisson*<br>*Dessert*<br>*Boisson*<br>*(Eau, café, thé, vin, bière)*	o 1 grand bol de soupe de courgettes fait maison (250 mL)<br>o 125 grammes de steak grillé (sans matière grasse) + 300 grammes de brocoli cuit à la vapeur + 100 grammes de pommes de terre nouvelles au four avec 5 g de beurre (solo)<br>o 3 cuillères à soupe de sauce béarnaise (prêt Delhaize)<br>o 2 verres de vin rouge
**SNACK**<br>*Fruit,yoghurt,koekjes, chocolade,chips,…*	o 1 poignée de chips (marque : Lays Pepper & Salt)<br>o 30 grammes de noix aux poivrons (marque : Duyvis)<br>o 3 biscuits oreo

BOISSONS

Verre d'eau	8 verres d'eau
L'alcool	2 verres de vin
Tasse café/thé	1 café + 3 thés

MOUVEMENT

Activité: promenade

Durée: 30 minutes

Fréquence: 4 fois par semaine
(par jour/par semaine)

Food diary

For 3 days (preferably 2 weekdays & 1 weekend day), keep a record of what you consume on a day in this food diary. Try to be as complete as possible by writing down the ***quantities (grams)*** *and the* ***way you prepare*** *the food.*
If possible, also mention the ***name/brand*** *of the product. Feel free to add pictures as well. (See example below)*

DAY 1 Date:.........................

	Portion (grams / number of pieces / tablespoons) / Brand / Preparation
**BREAKFAST**<br>*Bread/breakfast cereals/fruit*<br>*Grease*<br>*Sweet toppings (jam, chocolate...)*<br>*Savoury toppings (cheese, cold meat)*<br>*Dairy products*<br>*Beverages*<br>*(water, coffee, tea, fruit juice,...)*	
**SNACK**<br>*Fruit,yoghurt,biscuits,*<br>*chocolate,chips,…*	
**LUNCH**<br>*Bread / spreads*<br>*Soup*<br>*Meat/fish/vegetarian*<br>*Vegetables*<br>*Potatoes/rice/pasta*<br>*Cooking fat*<br>*Sauces*<br>*Dessert*<br>*Beverages*<br>*(water, coffee, tea, wine, beer,...)*	
**SNACK**<br>*Fruit,yoghurt,biscuits,*<br>*chocolate,chips,…*	
**DINNER**<br>*Bread / spreads*<br>*Soup*<br>*Meat/fish/vegetarian*<br>*Vegetables*<br>*Potatoes/rice/pasta*<br>*Cooking fat*<br>*Sauces*<br>*Dessert*<br>*Beverages*<br>*(water, coffee, tea, wine, beer,...)*	
**LATE SNACK**<br>*Fruit,yoghurt,biscuits,*<br>*chocolate,chips,…*	

BEVERAGES

Waterglasses ....

Soft drink /fruit juice ....

Alcohol ....

Cup coffee/tea ....

ACTIVITY

Activity:

Duration:

Frequency:
(per day/week)

DAY 2 Date:........................

Meal	Portion (grams / number of pieces / tablespoons) / Brand / Preparation
**BREAKFAST**<br>*Bread/breakfast cereals/fruit*<br>*Grease*<br>*Sweet toppings (jam, chocolate...)*<br>*Savoury toppings (cheese, cold meat)*<br>*Dairy products*<br>*Beverages*<br>*(water, coffee, tea, fruit juice,...)*	
**SNACK**<br>*Fruit,yoghurt,biscuits,*<br>*chocolate,chips,…*	
**LUNCH**<br>*Bread / spreads*<br>*Soup*<br>*Meat/fish/vegetarian*<br>*Vegetables*<br>*Potatoes/rice/pasta*<br>*Cooking fat*<br>*Sauces*<br>*Dessert*<br>*Beverages*<br>*(water, coffee, tea, wine, beer,...)*	
**SNACK**<br>*Fruit,yoghurt,biscuits,*<br>*chocolate,chips,…*	
**DINNER**<br>*Bread / spreads*<br>*Soup*<br>*Meat/fish/vegetarian*<br>*Vegetables*<br>*Potatoes/rice/pasta*<br>*Cooking fat*<br>*Sauces*<br>*Dessert*<br>*Beverages*<br>*(water, coffee, tea, wine, beer,...)*	
**LATE SNACK**<br>*Fruit,yoghurt,biscuits,*<br>*chocolate,chips,…*	

BEVERAGES

Waterglasses ....

Soft drink /fruit juice ....

Alcohol ....

Cup coffee/tea ....

ACTIVITY

Activity:

Duration:

Frequency:
(per day/week)

DAY 3 Date:……………………

	Portion (grams / number of pieces / tablespoons) / Brand / Preparation
**BREAKFAST**<br>*Bread/breakfast cereals/fruit*<br>*Grease*<br>*Sweet toppings (jam, chocolate...)*<br>*Savoury toppings (cheese, cold meat)*<br>*Dairy products*<br>*Beverages*<br>*(water, coffee, tea, fruit juice,...)*	
**SNACK**<br>*Fruit,yoghurt,biscuits,*<br>*chocolate,chips,…*	
**LUNCH**<br>*Bread / spreads*<br>*Soup*<br>*Meat/fish/vegetarian*<br>*Vegetables*<br>*Potatoes/rice/pasta*<br>*Cooking fat*<br>*Sauces*<br>*Dessert*<br>*Beverages*<br>*(water, coffee, tea, wine, beer,...)*	
**SNACK**<br>*Fruit,yoghurt,biscuits,*<br>*chocolate,chips,…*	
**DINNER**<br>*Bread / spreads*<br>*Soup*<br>*Meat/fish/vegetarian*<br>*Vegetables*<br>*Potatoes/rice/pasta*<br>*Cooking fat*<br>*Sauces*<br>*Dessert*<br>*Beverages*<br>*(water, coffee, tea, wine, beer,...)*	
**LATE SNACK**<br>*Fruit,yoghurt,biscuits,*<br>*chocolate,chips,…*	

BEVERAGES

Waterglasses ……………………………

Soft drink /fruit juice ……………………………

Alcohol ……………………………

Cup coffee/tea ……………………………

ACTIVITY

Activity:

Duration:

Frequency:
(per day/week)

EXAMPLE

Date: 31 March 2022

	Portion (grams / number of pieces / tablespoons) / Brand / Preparation
**BREAKFAST**<br>*Bread/breakfast cereals/fruit*<br>*Grease*<br>*Sweet toppings (jam, chocolate...)*<br>*Savoury toppings (cheese, cold meat)*<br>*Dairy products*<br>*Beverages*<br>*(water, coffee, tea, fruit juice,...)*	o 50 gr oatmeal + 250 ml unsweetend almond milk (alpro) + 1 teaspoon stevia + 1 handful blueberries & raspberries<br>o 1 cup of coffee (125 mL) + 2 teaspoons skimmed milk<br>o 1 grey bread slice, 1 pinch of margarine, 1 slice of gouda cheese
**SNACK**<br>*Fruit, yoghurt, biscuits, chocolate, chips,…*	o 1 x Kiwi<br>o 1 x full-fat yoghurt (Danone)
**LUNCH**<br>*Bread / spreads*<br>*Soup*<br>*Meat/fish/vegetarian*<br>*Vegetables*<br>*Potatoes/rice/pasta*<br>*Cooking fat*<br>*Sauces*<br>*Dessert*<br>*Beverages*<br>*(water, coffee, tea, wine, beer,...)*	o 3 grey bread slices + 1 pinch of minarine + 3 slices of chicken white with herbs (Delhaize)<br>o 2 milk sandwiches + 3 pinches of chocolate spread (Nutella)<br>o 3 cups of tea (125 mL) + 1 teaspoon of honey + 1 cloud of milk
**SNACK**<br>*Fruit, yoghurt, biscuits, chocolate, chips,…*	o 1 slice Cheesecake with speculoos (Tarte de Françoise)
**DINNER**<br>*Bread / spreads*<br>*Soup*<br>*Meat/fish/vegetarian*<br>*Vegetables*<br>*Potatoes/rice/pasta*<br>*Cooking fat*<br>*Sauces*<br>*Dessert*<br>*Beverages*<br>*(water, coffee, tea, wine, beer,...)*	o 1 big cupof homemade zucchini soup (250 mL)<br>o 125 gram grilled steak (no coocking fat) + 300 gram steamed broccoli + 100 gram fried fingerling potatoes with 5 g butter(solo)<br>o 3 tablespoons Bearnaise sauce (Delhaize ready-to-eat)<br>o 2 glasses of red wine
**SNACK**<br>*Fruit, yoghurt, biscuits, chocolate, chips,…*	o 1 handful chips (brand: Lays Pepper & Salt)<br>o 30 gram of peanuts (brand: Duyvis)<br>o 3 oreo cookies

BEVERAGES

Water glasses	8 glasses water
Soft drink /fruit juice	0
Alcohol	2 glasses wine
Cup of coffee/tea	1 x coffee/<br>3 x tea

ACTIVITY

Activity: Walking

Duration: 30 minutes

Frequency: 4 times a week
(per day/week)
