# Supplementary material for: Agreement and reliability between the two-day 6-minute incremental step test and two-day cardiopulmonary exercise test in post COVID-19 condition for assessing post-exertional malaise: The REVEAL-study
Source: PLoS One. 2026 Jul 14;21(7):e0353132. doi: 10.1371/journal.pone.0353132 (PMC13367738; doi:10.1371/journal.pone.0353132)
Supplement: Attachment — Submitted filename: Response to reviewers.docx [file pone.0353132.s005.docx]

Brussels, May 18, 2026

Dear Editor,

First, we would like to thank you for giving us the opportunity to revise and resubmit the manuscript. Second, we would like to thank the reviewers for the helpful comments and suggestions regarding our manuscript “*Agreement and reliability between the two-day 6-minute incremental step test and two-day cardiopulmonary exercise test in post COVID-19 condition for assessing post-exertional malaise: the REVEAL-study*”.

All revisions can be found with track changes in the MS Word document and are marked in yellow as well. Furthermore, we have added a point-by-point response to the concerns of the reviewers. We hope the revised version of our manuscript will meet the standards of your journal.

With my sincere thanks,

Yours faithfully,

The authors

Response to reviewers’ comments:

**ACADEMIC EDITOR – REMARK 1:** Please ensure that your manuscript meets PLOS ONE's style requirements, including those for file naming.

RESPONSE TO THE EDITOR: We thank the academic editor for this valuable remark. We have revised the manuscript to comply with PLOS ONE style requirements and have updated it accordingly.

**ACADEMIC EDITOR – REMARK 2:** Thank you for submitting your clinical trial to PLOS ONE and for providing the name of the registry and the registration number. The information in the registry entry suggests that your trial was registered after patient recruitment began. PLOS ONE strongly encourages authors to register all trials before recruiting the first participant in a study.

As per the journal’s editorial policy, please include in the Methods section of your paper:

1) your reasons for your delay in registering this study (after enrolment of participants started);

2) confirmation that all related trials are registered by stating: “The authors confirm that all ongoing and related trials for this drug/intervention are registered”.

RESPONSE TO THE EDITOR: We thank the academic editor for these important comments and have revised the Methods section accordingly. The reasons for delay in registration of the study and the statement about registration of all related trials have been added to the Methods section.

We added:

“The delay in registration was due to fixed logistical constraints, including pre-scheduled access to cardiopulmonary exercise testing facilities, which required the study to start within a limited time window. The trial was registered in ClinicalTrials.gov as soon as possible, and all outcomes and the study protocol were defined a priori and remained unchanged during this period. The authors confirm that all ongoing and related trials for this intervention are registered.”

**ACADEMIC EDITOR – REMARK 3:** Thank you for stating the following financial disclosure:

“Funding was provided by Fonds Wetenschappelijk Onderzoek (FWO) with grant number G075423N.” Please state what role the funders took in the study.  If the funders had no role, please state: "The funders had no role in study design, data collection and analysis, decision to publish, or preparation of the manuscript." If this statement is not correct you must amend it as needed. Please include this amended Role of Funder statement in your cover letter; we will change the online submission form on your behalf.

RESPONSE TO THE EDITOR: We thank the academic editor for this valuable comment. The financial disclosure has been added to the section “Declarations” and to the cover letter. The Role of Funder statement has been added to the cover letter.

**ACADEMIC EDITOR – REMARK 4:** Your ethics statement should only appear in the Methods section of your manuscript. If your ethics statement is written in any section besides the Methods, please delete it from any other section.

RESPONSE TO THE EDITOR: We thank the academic editor for this clarification. We have revised the manuscript to ensure that the ethics statement appears only in the Methods section. Any duplicate instances in other sections have been removed.

**ACADEMIC EDITOR – REMARK 5:** We note that the original protocol that you have uploaded as a Supporting Information file contains an institutional logo. As this logo is likely copyrighted, we ask that you please remove it from this file and upload an updated version upon resubmission.

RESPONSE TO THE EDITOR: We thank the academic editor for this observation. The institutional logo has been removed from the protocol file and an updated version has been uploaded with the revised submission.

**ACADEMIC EDITOR – REMARK 6:** If the reviewer comments include a recommendation to cite specific previously published works, please review and evaluate these publications to determine whether they are relevant and should be cited. There is no requirement to cite these works unless the editor has indicated otherwise.

RESPONSE TO THE EDITOR: We thank the academic editor for this clarification. We carefully reviewed the references suggested by the reviewers and incorporated the publications that were considered relevant to the aims and scientific context of the present study.

**REVIEWER 1 – REMARK 1:** The manuscript addresses an interesting and clinically relevant topic. The study is well designed, and the results are clearly presented. However, the manuscript would benefit from strengthening the scientific background and contextualization within the existing literature on functional capacity testing, reliability of field tests, tele-assessment and comparison with CPET.

RESPONSE TO THE REVIEWER: We thank the reviewer for this nicely worded feedback. We agree that strengthening the background and contextualization within the relevant literature would improve the manuscript. Accordingly, we revised the background section to better situate the study within the existing literature (see following remarks).

**REVIEWER 1 – REMARK 2:** Introduction
The introduction currently provides limited background on the reliability and clinical utility of field exercise tests (e.g., shuttle tests, 6MWT) as alternatives or complements to CPET.
Please expand this section and better justify the use of accessible functional tests and wearable technologies. The rationale for home-based and remote assessment is highly relevant but insufficiently supported by literature. Please include evidence from cardiac rehabilitation and tele-monitoring research, particularly post-COVID developments.
When discussing functional capacity testing and interpretation of exercise performance, the manuscript would benefit from citing studies on predictors and clinical interpretation of walking tests. Please strengthen the background regarding reliability and clinical use of field walking/stepping tests.
Add citations when discussing reliability, accessibility, and alternatives to CPET:
https://doi.org/10.1016/j.physio.2009.11.010

https://doi.org/10.12968/ijtr.2021.0046
These papers provide important evidence on long-term reliability and clinical utility of incremental shuttle/field tests in cardiovascular rehabilitation populations and support the rationale for exploring accessible alternatives to CPET.

RESPONSE TO THE REVIEWER: We thank the reviewer for this constructive comment. We agree that the introduction needed strengthening of the scientific background and required a stronger contextualization within the existing literature.

We added

“Field-based exercise tests, such as the incremental shuttle walking test or 6-Minute Walk Test, have been widely used as practical alternatives or complements to CPET in various clinical populations. These tests have demonstrated good reliability and clinical utility, especially in cardiovascular and cardiopulmonary disorders, where they are commonly used to assess functional capacity and guide treatment decisions. In cardiovascular rehabilitation populations, incremental field tests have been shown to provide reproducible and clinically interpretable measures of functional capacity over time, supporting their use in both assessment and longitudinal follow-up. These field-based tests are valuable due to their accessibility, low cost and ease of implementation.

Recent advances in wearable sensor technologies further enhance the potential of field- and home-based exercise testing by enabling continuous, objective monitoring of physiological responses such as heart rate and oxygen consumption in real-world environments. This is particularly significant in light of the COVID-19 pandemic, which has accelerated the implementation of telemonitoring and remote rehabilitation strategies. Evidence from cardiac rehabilitation and telehealth research demonstrates that remote assessment and monitoring can be safe and effective, while reducing patient burden and improving accessibility to care. In individuals with post COVID-19 condition, who experience pronounced fatigue and PEM, the ability to perform functional assessments in a home-based setting may be particularly valuable.” Suggested references are added in the revised manuscript.

**REVIEWER 1 – REMARK 3:** Methods / Rationale. The manuscript uses wearable sensors and portable metabolic systems; therefore, the technological and methodological context should be strengthened by citing recent work on automated or technology-supported functional capacity testing.

RESPONSE TO THE REVIEWER: We thank the reviewer for this comment.

We expanded the Introduction/Rationale section to strengthen the technological and methodological rationale for using wearable sensors and portable metabolic systems in the context of functional capacity assessment and home-based monitoring and added relevant references (see REVIEWER 1 – REMARK 2).

**REVIEWER 1 – REMARK 4:** Discussion
The discussion should better contextualize the findings within the broader literature on:
reliability of field tests
remote and tele-assessment of functional capacity
agreement between field tests and CPET
Since the main aim is comparison with CPET, it is essential to cite recent systematic evidence on the validity and reliability of field walking tests compared with CPET.
The clinical implications section would benefit from stronger support regarding the feasibility of home-based and remote monitoring approaches.
Predictors and interpretation of 6-minute walk/functional capacity tests

RESPONSE TO THE REVIEWER: We thank the reviewer for this helpful comment. We agree that the discussion would benefit from a deeper connection to existing literature. We have therefore expanded the discussion section and added suggested references in the revised manuscript (see following remarks).

**REVIEWER 1 – REMARK 5:** Discussion

Predictors and interpretation of 6-minute walk/functional capacity tests
When discussing functional capacity assessment and interpretation of exercise testing outcomes, the manuscript would benefit from including evidence on determinants and clinical interpretation of field tests.
Add citation in Introduction/Discussion:
doi: 10.1590/1414-431X2020e10514
https://doi.org/10.12968/bjca.2015.10.9.454
These references provides relevant background on predictors and clinical interpretation of functional capacity tests.

RESPONSE TO THE REVIEWER: We thank the reviewer for this comment. We agree that discussing determinants and clinical interpretation of field tests would strengthen the manuscript.

We added:
“Functional capacity tests such as walking and stepping tests are increasingly used in clinical practice because they provide accessible and clinically meaningful information regarding exercise tolerance. Previous literature has shown that the interpretation of these tests is multifactorial-for instance, the 6-minute walking test result should take into account age, stature and BMI in individuals with chronic heart failure. The 6MIST (but without a structured increase in exercise intensity), has been previously investigated and validated as an equivalent alternative to the standard 6-minute walk test in the same population.” Suggested references are added to the revised manuscript.

**REVIEWER 1 – REMARK 6:** Discussion

Because the authors highlight the possibility of performing the test at home, literature on tele-assessment and repeated remote measurements should be acknowledged.
Please cite: https://doi.org/10.3390/s23031354
They are directly relevant to remote assessment of functional capacity and supports the feasibility of wearable-based and home-based testing approaches.

RESPONSE TO THE REVIEWER: We thank the reviewer for this valuable suggestion and agree that relevant literature on tele-assessment and repeated remote measurements should be acknowledged, given our emphasis on home-based testing.

We added:

“The observed lack of agreement and reliability in the two-day 6MIST likely stems from methodological and protocol-related factors—rather than a lack of clinical relevance—while its potential applicability in home-based settings remains clinically meaningful despite these limitations. The COVID-19 pandemic accelerated the implementation of home-based telerehabilitation, where remote functional assessments have gained adoption. A recent study demonstrated that wearable technologies and remote assessment methods are feasible for measuring functional capacity beyond rehabilitation or hospital settings. Home-based assessment methods may be particularly valuable in individuals with post COVID-19 condition, who frequently suffer from fatigue and PEM because of travelling and hospital-based testing. Therefore, while the two-day 6MIST protocol would benefit from further refinement and validation, its accessibility and feasibility suggest potential for exploration as a home-based assessment tool.” Suggested references are added in the revised manuscript.

**REVIEWER 1 – REMARK 7:** Discussion

Since the main aim is comparison with CPET, the manuscript should include a recent systematic review/meta-analysis on validity vs CPET.
Please cite:
https://doi.org/10.1136/bmjresp-2021-001158
https://doi.org/10.3390/jcm14238303
DOI:10.1136/bmjresp-2021-001158

This is highly relevant and should be cited in both Introduction and Discussion when interpreting agreement between field tests and CPET.

RESPONSE TO THE REVIEWER: We thank the reviewer for this observation and agree that a recent systematic review with meta-analysis is highly relevant.

We added:

“Field-based exercise tests are increasingly studied as an accessible alternative to CPET in different population groups. A recent systematic review with meta-analysis showed a moderately strong association between the 6-minute walking test and CPET- particularly peak VO_2_ - and with excellent test-retest reliability, supporting their validity as practical alternative when CPET is not feasible. In addition, another study has demonstrated significant and moderate associations between a new incremental step test and the 6-minute walking test in people with COPD. However, although these findings suggest that field-based exercise tests are clinically useful in other populations, their ability to capture PEM-related physiological changes in post COVID-19 condition has not yet been established.” Suggested references are added to the revised manuscript.

**REVIEWER 2 – REMARK 1:** Given the small sample size, the Shapiro–Wilk test may have limited power. A non-significant SW test does not confirm that the data are normally distributed.

RESPONSE TO THE REVIEWER: We thank the reviewer for this comment. We acknowledge that, in small samples, normality tests such as the Shapiro–Wilk test have limited power and that non-significant results should not be interpreted as proof of normality. Following consultation with the professional statistician Prof. Dr. Kurt Barbé (member of SQUARE (Support for Quantitative & Qualitative Research), core facility of the Vrije Universiteit Brussel for statistical consultancy), we used the Shapiro–Wilk test as part of the exploratory assessment of data distribution, as it is widely recommended for small-to-moderate sample sizes. Furthermore, the primary analyses applied in this study (ICC and Bland–Altman analysis) are generally considered robust to moderate deviations from normality. Therefore, we believe that the chosen statistical approach remains appropriate. Nevertheless, we remain open to the reviewer’s suggestions and feedback.

**REVIEWER 2 – REMARK 2:** No formal power calculation was provided. This limitation should be acknowledged, and the non-significant Wilcoxon signed-rank and paired t-test results should not be interpreted as conclusive evidence of no difference.

RESPONSE TO THE REVIEWER: We thank the reviewer for this valuable remark.

We agree that this should be addressed in the limitations section and that the non-significant Wilcoxon signed-rank and paired t-tests should be interpreted with caution.

We added (to the strengths and limitations section):

“However, no formal a priori power calculation was performed due to the absence of established MCIDs for the outcomes in this population. As such, the study may be underpowered to detect small differences between conditions. Non-significant findings, such as those from the non-significant Wilcoxon signed-rank test and paired t-tests, should therefore be interpreted with caution.”

**REVIEWER 2 – REMARK 3:** ICC is not sufficient to establish interchangeability because it mainly measures relative consistency or ranking, rather than whether two methods produce the same or similar values.

RESPONSE TO THE REVIEWER: We thank the reviewer for this important remark. We agree that ICC alone does not establish strict interchangeability or absolute agreement between two measurement methods. However, the primary aim of the present study was not to demonstrate that the two-day CPET and two-day 6MIST yield identical absolute values, but rather to evaluate whether both tests consistently capture the presence and relative magnitude of post-exertional malaise (PEM) across participants. Following consultation with the professional statistician Prof. Dr. Kurt Barbé, we therefore used a consistency ICC, as this approach is appropriate when assessing relative consistency rather than absolute agreement. In addition, Bland–Altman analysis was included to further explore agreement and potential systematic bias between methods. Nevertheless, we appreciate and respect the reviewer’s perspective and hope that the clarification provided addresses this concern.

We added (to aim of the study):

“Importantly, this study did not aim to establish absolute equivalence of outcome values between the two-day 6MIST and two-day CPET. Instead, the focus was on their concordance in detecting within-subject change over time, specifically the extent to which both protocols elicit a comparable decline in physiological capacity and symptom burden from day 1 to day 2 as an indicator of post-exertional malaise.”

**REVIEWER 2 – REMARK 4:** Clarify what ICC model 3.1 is.

RESPONSE TO THE REVIEWER: We thank the reviewer for this comment.

We agree that the ICC model should be clarified.

We changed:

“We selected the consistency-based ICC model 3.1 because it assumes that all participants are assessed by the same fixed rater and it is appropriate for determining whether the pattern of repeated scores remains stable across days.”

to

“We selected the ICC (3,1) model, corresponding to a two-way mixed-effects, single-measurement, consistency model. This model assumes fixed measurement conditions and evaluates whether the pattern of repeated scores remains stable across days.”

We have added the following citation: Shrout PE, Fleiss JL. Intraclass correlations: uses in assessing rater reliability. Psychol Bull. 1979;86(2):420–428. doi:10.1037/0033-2909.86.2.420.

**REVIEWER 2 – REMARK 5:** Line 255: specify the cutoff values used. Line 257: The wording is too strong because the true score is not known.

RESPONSE TO THE REVIEWER: We thank the reviewer for this comment.

We added (line 255):

“Based on the 95% confidence interval of the ICC estimate, values below 0.50, between 0.50 and 0.75, between 0.75 and 0.90 and above 0.90 indicate poor, moderate, good, and excellent reliability, respectively.”

We changed (line 257):

“The SEM estimates the average deviation from the participant’s true score due to measurement variability. A smaller SEM means the test is more precise.”

to

“The SEM estimates the amount of measurement variability expected in repeated assessments, with smaller SEM values indicating greater measurement precision.”

**REVIEWER 2 – REMARK 6:** Figure 1: In experimental session 2, two-day CPET, the sample size should be N = 10.

RESPONSE TO THE REVIEWER: We thank the reviewer for pointing this out. We have updated Figure 1 accordingly, changing the indicated sample size from N = 12 to N = 10.

**REVIEWER 2 – REMARK 7:** Use decimal points instead of commas throughout the manuscript.

RESPONSE TO THE REVIEWER: We thank the reviewer for this observation. All decimal commas in the manuscript have now been changed to decimal points.
