# Supplementary material for: Agreement and reliability between the two-day 6-minute incremental step test and two-day cardiopulmonary exercise test in post COVID-19 condition for assessing post-exertional malaise: The REVEAL-study
Source: PLoS One. 2026 Jul 14;21(7):e0353132. doi: 10.1371/journal.pone.0353132 (PMC13367738; doi:10.1371/journal.pone.0353132)
Supplement: Attachment — Submitted filename: Response_to_Reviewers_auresp_2.docx [file pone.0353132.s006.docx]

Brussels, June 9, 2026

Dear Editor,

First, we would like to thank you for giving us the opportunity to revise and resubmit the manuscript. Second, we would like to thank the reviewers for the helpful comments and suggestions regarding our manuscript “*Agreement and reliability between the two-day 6-minute incremental step test and two-day cardiopulmonary exercise test in post COVID-19 condition for assessing post-exertional malaise: the REVEAL-study*”.

All revisions can be found with track changes in the MS Word document. Furthermore, we have added a point-by-point response to the concerns of the reviewers. We hope the revised version of our manuscript will meet the standards of your journal.

With my sincere thanks,

Yours faithfully,

The authors

Response to reviewers’ comments:

**ACADEMIC EDITOR – REMARK 1:** Check format of references. So many mistakes in references, ie 19. Rewrite all references.

RESPONSE TO ACADEMIC EDITOR: We thank the academic editor for this important comment. We have systematically reviewed the entire reference list and corrected all inconsistencies and formatting errors. All references have been revised to comply with the PLOS One Vancouver style with square brackets. The reference list has been fully standardized and verified for consistency.

**REVIEWER 1 – REMARK 1:** Check format of references. So many mistakes in references, ie 19. Rewrite all references. You can use endnote.

RESPONSE TO REVIEWER: We thank the reviewer for their comment. See ACADEMIC EDITOR – REMARK 1.
