# Supplementary material for: Agreement and reliability between the two-day 6-minute incremental step test and two-day cardiopulmonary exercise test in post COVID-19 condition for assessing post-exertional malaise: The REVEAL-study
Source: PLoS One. 2026 Jul 14;21(7):e0353132. doi: 10.1371/journal.pone.0353132 (PMC13367738; doi:10.1371/journal.pone.0353132)
Supplement: Peer review history

# Peer review: Decision Letter 0

**DOI:** 10.1371/journal.pone.0353132.r001

**Authors:** Vincenzo Lionetti

29 Apr 2026

PONE-D-26-16093

Agreement and reliability between the two-day 6-minute incremental step test and two-day cardiopulmonary exercise test in post COVID-19 condition for assessing post-exertional malaise: the REVEAL-study

PLOS One

Dear Dr. Bomans,

Thank you for submitting your manuscript to PLOS ONE. After careful consideration, we feel that it has merit but does not fully meet PLOS ONE’s publication criteria as it currently stands. Therefore, we invite you to submit a revised version of the manuscript that addresses the points raised during the review process.

Please submit your revised manuscript by Jun 13 2026 11:59PM. If you will need more time than this to complete your revisions, please reply to this message or contact the journal office at plosone@plos.org. When you're ready to submit your revision, log on to https://www.editorialmanager.com/pone/ and select the 'Submissions Needing Revision' folder to locate your manuscript file.

Please include the following items when submitting your revised manuscript:

- A letter that responds to each point raised by the academic editor and reviewer(s). You should upload this letter as a separate file labeled 'Response to Reviewers'.
- A marked-up copy of your manuscript that highlights changes made to the original version. You should upload this as a separate file labeled 'Revised Manuscript with Track Changes'.
- An unmarked version of your revised paper without tracked changes. You should upload this as a separate file labeled 'Manuscript'.

If you would like to make changes to your financial disclosure, please include your updated statement in your cover letter. Guidelines for resubmitting your figure files are available below the reviewer comments at the end of this letter.

If applicable, we recommend that you deposit your laboratory protocols in protocols.io to enhance the reproducibility of your results. Protocols.io assigns your protocol its own identifier (DOI) so that it can be cited independently in the future. For instructions see: https://journals.plos.org/plosone/s/submission-guidelines#loc-laboratory-protocols. Additionally, PLOS ONE offers an option for publishing peer-reviewed Lab Protocol articles, which describe protocols hosted on protocols.io. Read more information on sharing protocols at https://plos.org/protocols?utm_medium=editorial-email&utm_source=authorletters&utm_campaign=protocols.

As the corresponding author, your ORCID iD is verified in the submission system and will appear in the published article. PLOS supports the use of ORCID, and we encourage all coauthors to register for an ORCID iD and use it as well. Please encourage your coauthors to verify their ORCID iD within the submission system before final acceptance, as unverified ORCID iDs will not appear in the published article. *Only*  the individual author can complete the verification step; PLOS staff *cannot*  verify ORCID iDs on behalf of authors.

We look forward to receiving your revised manuscript.

Kind regards,

Vincenzo Lionetti, M.D., PhD

Academic Editor

PLOS One

Journal Requirements:

When submitting your revision, we need you to address these additional requirements.

1.Please ensure that your manuscript meets PLOS ONE's style requirements, including those for file naming. The PLOS ONE style templates can be found at

https://journals.plos.org/plosone/s/file?id=wjVg/PLOSOne_formatting_sample_main_body.pdf and

https://journals.plos.org/plosone/s/file?id=ba62/PLOSOne_formatting_sample_title_authors_affiliations.pdf

2. Thank you for submitting your clinical trial to PLOS ONE and for providing the name of the registry and the registration number. The information in the registry entry suggests that your trial was registered after patient recruitment began. PLOS ONE strongly encourages authors to register all trials before recruiting the first participant in a study.

As per the journal’s editorial policy, please include in the Methods section of your paper:

1) your reasons for your delay in registering this study (after enrolment of participants started);

2) confirmation that all related trials are registered by stating: “The authors confirm that all ongoing and related trials for this drug/intervention are registered”.

3. Thank you for stating the following financial disclosure:

“Funding was provided by Fonds Wetenschappelijk Onderzoek (FWO) with grant number G075423N.”

Please state what role the funders took in the study.  If the funders had no role, please state: "The funders had no role in study design, data collection and analysis, decision to publish, or preparation of the manuscript."

If this statement is not correct you must amend it as needed.

Please include this amended Role of Funder statement in your cover letter; we will change the online submission form on your behalf.

4. Your ethics statement should only appear in the Methods section of your manuscript. If your ethics statement is written in any section besides the Methods, please delete it from any other section.

5. We note that the original protocol that you have uploaded as a Supporting Information file contains an institutional logo. As this logo is likely copyrighted, we ask that you please remove it from this file and upload an updated version upon resubmission.

6. If the reviewer comments include a recommendation to cite specific previously published works, please review and evaluate these publications to determine whether they are relevant and should be cited. There is no requirement to cite these works unless the editor has indicated otherwise.

Additional Editor Comments:

All issues raised by expert reviewers are required.

[Note: HTML markup is below. Please do not edit.]

Reviewers' comments:

Reviewer's Responses to Questions

**Comments to the Author**

1. Is the manuscript technically sound, and do the data support the conclusions?

The manuscript must describe a technically sound piece of scientific research with data that supports the conclusions. Experiments must have been conducted rigorously, with appropriate controls, replication, and sample sizes. The conclusions must be drawn appropriately based on the data presented.

Reviewer #1: Yes

Reviewer #2: Partly

**********

2. Has the statistical analysis been performed appropriately and rigorously?

Reviewer #1: Yes

Reviewer #2: No

**********

3. Have the authors made all data underlying the findings in their manuscript fully available?

The PLOS Data policy requires authors to make all data underlying the findings described in their manuscript fully available without restriction, with rare exception (please refer to the Data Availability Statement in the manuscript PDF file). The data should be provided as part of the manuscript or its supporting information, or deposited to a public repository. For example, in addition to summary statistics, the data points behind means, medians and variance measures should be available. If there are restrictions on publicly sharing data—e.g. participant privacy or use of data from a third party—those must be specified.

Reviewer #1: Yes

Reviewer #2: Yes

**********

4. Is the manuscript presented in an intelligible fashion and written in standard English?

PLOS ONE does not copyedit accepted manuscripts, so the language in submitted articles must be clear, correct, and unambiguous. Any typographical or grammatical errors should be corrected at revision, so please note any specific errors here.

Reviewer #1: Yes

Reviewer #2: Yes

**********

5. Review Comments to the Author

Please use the space provided to explain your answers to the questions above. You may also include additional comments for the author, including concerns about dual publication, research ethics, or publication ethics. (Please upload your review as an attachment if it exceeds 20,000 characters)

Reviewer #1: The manuscript addresses an interesting and clinically relevant topic. The study is well designed and the results are clearly presented. However, the manuscript would benefit from strengthening the scientific background and contextualization within the existing literature on functional capacity testing, reliability of field tests, tele-assessment and comparison with CPET.

Introduction

The introduction currently provides limited background on the reliability and clinical utility of field exercise tests (e.g., shuttle tests, 6MWT) as alternatives or complements to CPET.

Please expand this section and better justify the use of accessible functional tests and wearable technologies.

The rationale for home-based and remote assessment is highly relevant but insufficiently supported by literature. Please include evidence from cardiac rehabilitation and tele-monitoring research, particularly post-COVID developments.

When discussing functional capacity testing and interpretation of exercise performance, the manuscript would benefit from citing studies on predictors and clinical interpretation of walking tests.

Please strengthen the background regarding reliability and clinical use of field walking/stepping tests.

Add citations when discussing reliability, accessibility, and alternatives to CPET:

https://doi.org/10.1016/j.physio.2009.11.010

https://doi.org/10.12968/ijtr.2021.0046

These papers provide important evidence on long-term reliability and clinical utility of incremental shuttle/field tests in cardiovascular rehabilitation populations and support the rationale for exploring accessible alternatives to CPET.

Methods / Rationale

The manuscript uses wearable sensors and portable metabolic systems; therefore, the technological and methodological context should be strengthened by citing recent work on automated or technology-supported functional capacity testing.

Discussion

The discussion should better contextualize the findings within the broader literature on:

reliability of field tests

remote and tele-assessment of functional capacity

agreement between field tests and CPET

Since the main aim is comparison with CPET, it is essential to cite recent systematic evidence on the validity and reliability of field walking tests compared with CPET.

The clinical implications section would benefit from stronger support regarding the feasibility of home-based and remote monitoring approaches.

Predictors and interpretation of 6-minute walk/functional capacity tests

-When discussing functional capacity assessment and interpretation of exercise testing outcomes, the manuscript would benefit from including evidence on determinants and clinical interpretation of field tests.

Add citation in Introduction/Discussion:

https://doi.org/10.12968/bjca.2015.10.9.454

This reference provides relevant background on predictors and clinical interpretation of functional capacity tests.

- Predictors and interpretation of 6-minute walk/functional capacity tests

When discussing functional capacity assessment and interpretation of exercise testing outcomes, the manuscript would benefit from including evidence on determinants and clinical interpretation of field tests.

Add citation in Introduction/Discussion:

doi: 10.1590/1414-431X2020e10514

https://doi.org/10.12968/bjca.2015.10.9.454

These references provides relevant background on predictors and clinical interpretation of functional capacity tests.

-Because the authors highlight the possibility of performing the test at home, literature on tele-assessment and repeated remote measurements should be acknowledged.

Please cite: https://doi.org/10.3390/s23031354

They are directly relevant to remote assessment of functional capacity and supports the feasibility of wearable-based and home-based testing approaches.

- Since the main aim is comparison with CPET, the manuscript should include a recent systematic review/meta-analysis on validity vs CPET.

Please cite:

https://doi.org/10.1136/bmjresp-2021-001158

https://doi.org/10.3390/jcm14238303

DOI:10.1136/bmjresp-2021-001158

This is highly relevant and should be cited in both Introduction and Discussion when interpreting agreement between field tests and CPET.

Reviewer #2: Given the small sample size, the Shapiro–Wilk test may have limited power. A non-significant SW test does not confirm that the data are normally distributed.

No formal power calculation was provided. This limitation should be acknowledged, and the non-significant Wilcoxon signed-rank and paired t-test results should not be interpreted as conclusive evidence of no difference.

ICC is not sufficient to establish interchangeability because it mainly measures relative consistency or ranking, rather than whether two methods produce the same or similar values.

Clarify what ICC model 3.1 is.

Line 255: specify the cutoff values used.

Line 257: The wording is too strong because the true score is not known.

Figure 1: In experimental session 2, two-day CPET, the sample size should be N = 10.

Use decimal points instead of commas throughout the manuscript.

**********

6. PLOS authors have the option to publish the peer review history of their article (what does this mean?). If published, this will include your full peer review and any attached files.

If you choose “no”, your identity will remain anonymous but your review may still be made public.

**Do you want your identity to be public for this peer review?**  For information about this choice, including consent withdrawal, please see our Privacy Policy.

Reviewer #1: No

Reviewer #2: No

**********

[NOTE: If reviewer comments were submitted as an attachment file, they will be attached to this email and accessible via the submission site. Please log into your account, locate the manuscript record, and check for the action link "View Attachments". If this link does not appear, there are no attachment files.]

To ensure your figures meet our technical requirements, please review our figure guidelines: https://journals.plos.org/plosone/s/figures

You may also use PLOS’s free figure tool, NAAS, to help you prepare publication quality figures: https://journals.plos.org/plosone/s/figures#loc-tools-for-figure-preparation.

NAAS will assess whether your figures meet our technical requirements by comparing each figure against our figure specifications.

# Author comment: Author response to Decision Letter 1

**DOI:** 10.1371/journal.pone.0353132.r002

20 May 2026

ACADEMIC EDITOR – REMARK 1: Please ensure that your manuscript meets PLOS ONE's style requirements, including those for file naming.

RESPONSE TO THE EDITOR: We thank the academic editor for this valuable remark. We have revised the manuscript to comply with PLOS ONE style requirements and have updated it accordingly.

ACADEMIC EDITOR – REMARK 2: Thank you for submitting your clinical trial to PLOS ONE and for providing the name of the registry and the registration number. The information in the registry entry suggests that your trial was registered after patient recruitment began. PLOS ONE strongly encourages authors to register all trials before recruiting the first participant in a study.

As per the journal’s editorial policy, please include in the Methods section of your paper:

1) your reasons for your delay in registering this study (after enrolment of participants started);

2) confirmation that all related trials are registered by stating: “The authors confirm that all ongoing and related trials for this drug/intervention are registered”.

RESPONSE TO THE EDITOR: We thank the academic editor for these important comments and have revised the Methods section accordingly. The reasons for delay in registration of the study and the statement about registration of all related trials have been added to the Methods section.

We added:

“The delay in registration was due to fixed logistical constraints, including pre-scheduled access to cardiopulmonary exercise testing facilities, which required the study to start within a limited time window. The trial was registered in ClinicalTrials.gov as soon as possible, and all outcomes and the study protocol were defined a priori and remained unchanged during this period. The authors confirm that all ongoing and related trials for this intervention are registered.”

ACADEMIC EDITOR – REMARK 3: Thank you for stating the following financial disclosure:

“Funding was provided by Fonds Wetenschappelijk Onderzoek (FWO) with grant number G075423N.” Please state what role the funders took in the study. If the funders had no role, please state: "The funders had no role in study design, data collection and analysis, decision to publish, or preparation of the manuscript." If this statement is not correct you must amend it as needed. Please include this amended Role of Funder statement in your cover letter; we will change the online submission form on your behalf.

RESPONSE TO THE EDITOR: We thank the academic editor for this valuable comment. The financial disclosure has been added to the section “Declarations” and to the cover letter. The Role of Funder statement has been added to the cover letter.

ACADEMIC EDITOR – REMARK 4: Your ethics statement should only appear in the Methods section of your manuscript. If your ethics statement is written in any section besides the Methods, please delete it from any other section.

RESPONSE TO THE EDITOR: We thank the academic editor for this clarification. We have revised the manuscript to ensure that the ethics statement appears only in the Methods section. Any duplicate instances in other sections have been removed.

ACADEMIC EDITOR – REMARK 5: We note that the original protocol that you have uploaded as a Supporting Information file contains an institutional logo. As this logo is likely copyrighted, we ask that you please remove it from this file and upload an updated version upon resubmission.

RESPONSE TO THE EDITOR: We thank the academic editor for this observation. The institutional logo has been removed from the protocol file and an updated version has been uploaded with the revised submission.

ACADEMIC EDITOR – REMARK 6: If the reviewer comments include a recommendation to cite specific previously published works, please review and evaluate these publications to determine whether they are relevant and should be cited. There is no requirement to cite these works unless the editor has indicated otherwise.

RESPONSE TO THE EDITOR: We thank the academic editor for this clarification. We carefully reviewed the references suggested by the reviewers and incorporated the publications that were considered relevant to the aims and scientific context of the present study.

REVIEWER 1 – REMARK 1: The manuscript addresses an interesting and clinically relevant topic. The study is well designed, and the results are clearly presented. However, the manuscript would benefit from strengthening the scientific background and contextualization within the existing literature on functional capacity testing, reliability of field tests, tele-assessment and comparison with CPET.

RESPONSE TO THE REVIEWER: We thank the reviewer for this nicely worded feedback. We agree that strengthening the background and contextualization within the relevant literature would improve the manuscript. Accordingly, we revised the background section to better situate the study within the existing literature (see following remarks).

REVIEWER 1 – REMARK 2: Introduction

The introduction currently provides limited background on the reliability and clinical utility of field exercise tests (e.g., shuttle tests, 6MWT) as alternatives or complements to CPET.

Please expand this section and better justify the use of accessible functional tests and wearable technologies. The rationale for home-based and remote assessment is highly relevant but insufficiently supported by literature. Please include evidence from cardiac rehabilitation and tele-monitoring research, particularly post-COVID developments.

When discussing functional capacity testing and interpretation of exercise performance, the manuscript would benefit from citing studies on predictors and clinical interpretation of walking tests. Please strengthen the background regarding reliability and clinical use of field walking/stepping tests.

Add citations when discussing reliability, accessibility, and alternatives to CPET:

https://doi.org/10.1016/j.physio.2009.11.010

https://doi.org/10.12968/ijtr.2021.0046

These papers provide important evidence on long-term reliability and clinical utility of incremental shuttle/field tests in cardiovascular rehabilitation populations and support the rationale for exploring accessible alternatives to CPET.

RESPONSE TO THE REVIEWER: We thank the reviewer for this constructive comment. We agree that the introduction needed strengthening of the scientific background and required a stronger contextualization within the existing literature.

We added

“Field-based exercise tests, such as the incremental shuttle walking test or 6-Minute Walk Test, have been widely used as practical alternatives or complements to CPET in various clinical populations. These tests have demonstrated good reliability and clinical utility, especially in cardiovascular and cardiopulmonary disorders, where they are commonly used to assess functional capacity and guide treatment decisions. In cardiovascular rehabilitation populations, incremental field tests have been shown to provide reproducible and clinically interpretable measures of functional capacity over time, supporting their use in both assessment and longitudinal follow-up. These field-based tests are valuable due to their accessibility, low cost and ease of implementation.

Recent advances in wearable sensor technologies further enhance the potential of field- and home-based exercise testing by enabling continuous, objective monitoring of physiological responses such as heart rate and oxygen consumption in real-world environments. This is particularly significant in light of the COVID-19 pandemic, which has accelerated the implementation of telemonitoring and remote rehabilitation strategies. Evidence from cardiac rehabilitation and telehealth research demonstrates that remote assessment and monitoring can be safe and effective, while reducing patient burden and improving accessibility to care. In individuals with post COVID-19 condition, who experience pronounced fatigue and PEM, the ability to perform functional assessments in a home-based setting may be particularly valuable.” Suggested references are added in the revised manuscript.

REVIEWER 1 – REMARK 3: Methods / Rationale. The manuscript uses wearable sensors and portable metabolic systems; therefore, the technological and methodological context should be strengthened by citing recent work on automated or technology-supported functional capacity testing.

RESPONSE TO THE REVIEWER: We thank the reviewer for this comment.

We expanded the Introduction/Rationale section to strengthen the technological and methodological rationale for using wearable sensors and portable metabolic systems in the context of functional capacity assessment and home-based monitoring and added relevant references (see REVIEWER 1 – REMARK 2).

REVIEWER 1 – REMARK 4: Discussion

The discussion should better contextualize the findings within the broader literature on:

reliability of field tests

remote and tele-assessment of functional capacity

agreement between field tests and CPET

Since the main aim is comparison with CPET, it is essential to cite recent systematic evidence on the validity and reliability of field walking tests compared with CPET.

The clinical implications section would benefit from stronger support regarding the feasibility of home-based and remote monitoring approaches.

Predictors and interpretation of 6-minute walk/functional capacity tests

RESPONSE TO THE REVIEWER: We thank the reviewer for this helpful comment. We agree that the discussion would benefit from a deeper connection to existing literature. We have therefore expanded the discussion section and added suggested references in the revised manuscript (see following remarks).

REVIEWER 1 – REMARK 5: Discussion

Predictors and interpretation of 6-minute walk/functional capacity tests

When discussing functional capacity assessment and interpretation of exercise testing outcomes, the manuscript would benefit from including evidence on determinants and clinical interpretation of field tests.

Add citation in Introduction/Discussion:

doi: 10.1590/1414-431X2020e10514

https://doi.org/10.12968/bjca.2015.10.9.454

These references provides relevant background on predictors and clinical interpretation of functional capacity tests.

RESPONSE TO THE REVIEWER: We thank the reviewer for this comment. We agree that discussing determinants and clinical interpretation of field tests would strengthen the manuscript.

We added:

“Functional capacity tests such as walking and stepping tests are increasingly used in clinical practice because they provide accessible and clinically meaningful information regarding exercise tolerance. Previous literature has shown that the interpretation of these tests is multifactorial-for instance, the 6-minute walking test result should take into account age, stature and BMI in individuals with chronic heart failure. The 6MIST (but without a structured increase in exercise intensity), has been previously investigated and validated as an equivalent alternative to the standard 6-minute walk test in the same population.” Suggested references are added to the revised manuscript.

REVIEWER 1 – REMARK 6: Discussion

Because the authors highlight the possibility of performing the test at home, literature on tele-assessment and repeated remote measurements should be acknowledged.

Please cite: https://doi.org/10.3390/s23031354

They are directly relevant to remote assessment of functional capacity and supports the feasibility of wearable-based and home-based testing approaches.

RESPONSE TO THE REVIEWER: We thank the reviewer for this valuable suggestion and agree that relevant literature on tele-assessment and repeated remote measurements should be acknowledged, given our emphasis on home-based testing.

We added:

“The observed lack of agreement and reliability in the two-day 6MIST likely stems from methodological and protocol-related factors—rather than a lack of clinical relevance—while its potential applicability in home-based settings remains clinically meaningful despite these limitations. The COVID-19 pandemic accelerated the implementation of home-based telerehabilitation, where remote functional assessments have gained adoption. A recent study demonstrated that wearable technologies and remote assessment methods are feasible for measuring functional capacity beyond rehabilitation or hospital settings. Home-based assessment methods may be particularly valuable in individuals with post COVID-19 condition, who frequently suffer from fatigue and PEM because of travelling and hospital-based testing. Therefore, while the two-day 6MIST protocol would benefit from further refinement and validation, its accessibility and feasibility suggest potential for exploration as a home-based assessment tool.” Suggested references are added in the revised manuscript.

REVIEWER 1 – REMARK 7: Discussion

Since the main aim is comparison with CPET, the manuscript should include a recent systematic review/meta-analysis on validity vs CPET.

Please cite:

https://doi.org/10.1136/bmjresp-2021-001158

https://doi.org/10.3390/jcm14238303

DOI:10.1136/bmjresp-2021-001158

This is highly relevant and should be cited in both Introduction and Discussion when interpreting agreement between field tests and CPET.

RESPONSE TO THE REVIEWER: We thank the reviewer for this observation and agree that a recent systematic review with meta-analysis is highly relevant.

We added:

“Field-based exercise tests are increasingly studied as an accessible alternative to CPET in different population groups. A recent systematic review with meta-analysis showed a moderately strong association between the 6-minute walking test and CPET- particularly peak VO2 - and with excellent test-retest reliability, supporting their validity as practical alternative when CPET is not feasible. In addition, another study has demonstrated significant and moderate associations between a new incremental step test and the 6-minute walking test in people with COPD. However, although these findings suggest that field-based exercise tests are clinically useful in other populations, their ability to capture PEM-related physiological changes in post COVID-19 condition has not yet been established.” Suggested references are added to the revised manuscript.

REVIEWER 2 – REMARK 1: Given the small sample size, the Shapiro–Wilk test may have limited power. A non-significant SW test does not confirm that the data are normally distributed.

RESPONSE TO THE REVIEWER: We thank the reviewer for this comment. We acknowledge that, in small samples, normality tests such as the Shapiro–Wilk test have limited power and that non-significant results should not be interpreted as proof of normality. Following consultation with the professional statistician Prof. Dr. Kurt Barbé (member of SQUARE (Support for Quantitative & Qualitative Research), core facility of the Vrije Universiteit Brussel for statistical consultancy), we used the Shapiro–Wilk test as part of the exploratory assessment of data distribution, as it is widely recommended for small-to-moderate sample sizes. Furthermore, the primary analyses applied in this study (ICC and Bland–Altman analysis) are generally considered robust to moderate deviations from normality. Therefore, we believe that the chosen statistical approach remains appropriate. Nevertheless, we remain open to the reviewer’s suggestions and feedback.

REVIEWER 2 – REMARK 2: No formal power calculation was provided. This limitation should be acknowledged, and the non-significant Wilcoxon signed-rank and paired t-test results should not be interpreted as conclusive evidence of no difference.

RESPONSE TO THE REVIEWER: We thank the reviewer for this valuable remark.

We agree that this should be addressed in the limitations section and that the non-significant Wilcoxon signed-rank and paired t-tests should be interpreted with caution.

We added (to the strengths and limitations section):

“However, no formal a priori power calculation was performed due to the absence of established MCIDs for the outcomes in this population. As such, the study may be underpowered to detect small differences between conditions. Non-significant findings, such as those from the non-significant Wilcoxon

Attachment Submitted filename: Response to reviewers.docx

# Peer review: Decision Letter 1

**DOI:** 10.1371/journal.pone.0353132.r003

**Authors:** Vincenzo Lionetti

27 May 2026

PONE-D-26-16093R1

Agreement and reliability between the two-day 6-minute incremental step test and two-day cardiopulmonary exercise test in post COVID-19 condition for assessing post-exertional malaise: the REVEAL-study

PLOS One

Dear Dr. Bomans,

Thank you for submitting your manuscript to PLOS ONE. After careful consideration, we feel that it has merit but does not fully meet PLOS ONE’s publication criteria as it currently stands. Therefore, we invite you to submit a revised version of the manuscript that addresses the points raised during the review process.

==============================

**ACADEMIC EDITOR:** Check format of references. So many mistakes in references , ie 19. Rewrite all references.

==============================

Please submit your revised manuscript by Jul 11 2026 11:59PM. If you will need more time than this to complete your revisions, please reply to this message or contact the journal office at plosone@plos.org. When you're ready to submit your revision, log on to https://www.editorialmanager.com/pone/ and select the 'Submissions Needing Revision' folder to locate your manuscript file.

Please include the following items when submitting your revised manuscript:

- A letter that responds to each point raised by the academic editor and reviewer(s). You should upload this letter as a separate file labeled 'Response to Reviewers'.
- A marked-up copy of your manuscript that highlights changes made to the original version. You should upload this as a separate file labeled 'Revised Manuscript with Track Changes'.
- An unmarked version of your revised paper without tracked changes. You should upload this as a separate file labeled 'Manuscript'.

If you would like to make changes to your financial disclosure, please include your updated statement in your cover letter. Guidelines for resubmitting your figure files are available below the reviewer comments at the end of this letter.

If applicable, we recommend that you deposit your laboratory protocols in protocols.io to enhance the reproducibility of your results. Protocols.io assigns your protocol its own identifier (DOI) so that it can be cited independently in the future. For instructions see: https://journals.plos.org/plosone/s/submission-guidelines#loc-laboratory-protocols. Additionally, PLOS ONE offers an option for publishing peer-reviewed Lab Protocol articles, which describe protocols hosted on protocols.io. Read more information on sharing protocols at https://plos.org/protocols?utm_medium=editorial-email&utm_source=authorletters&utm_campaign=protocols.

As the corresponding author, your ORCID iD is verified in the submission system and will appear in the published article. PLOS supports the use of ORCID, and we encourage all coauthors to register for an ORCID iD and use it as well. Please encourage your coauthors to verify their ORCID iD within the submission system before final acceptance, as unverified ORCID iDs will not appear in the published article. *Only*  the individual author can complete the verification step; PLOS staff *cannot*  verify ORCID iDs on behalf of authors.

We look forward to receiving your revised manuscript.

Kind regards,

Vincenzo Lionetti, M.D., PhD

Academic Editor

PLOS One

Journal Requirements:

If the reviewer comments include a recommendation to cite specific previously published works, please review and evaluate these publications to determine whether they are relevant and should be cited. There is no requirement to cite these works unless the editor has indicated otherwise.

Please review your reference list to ensure that it is complete and correct. If you have cited papers that have been retracted, please include the rationale for doing so in the manuscript text, or remove these references and replace them with relevant current references. Any changes to the reference list should be mentioned in the rebuttal letter that accompanies your revised manuscript. If you need to cite a retracted article, indicate the article’s retracted status in the References list and also include a citation and full reference for the retraction notice.

[Note: HTML markup is below. Please do not edit.]

Reviewers' comments:

Reviewer's Responses to Questions

**Comments to the Author**

1. If the authors have adequately addressed your comments raised in a previous round of review and you feel that this manuscript is now acceptable for publication, you may indicate that here to bypass the “Comments to the Author” section, enter your conflict of interest statement in the “Confidential to Editor” section, and submit your "Accept" recommendation.

Reviewer #1: All comments have been addressed

Reviewer #2: All comments have been addressed

**********

2. Is the manuscript technically sound, and do the data support the conclusions?

The manuscript must describe a technically sound piece of scientific research with data that supports the conclusions. Experiments must have been conducted rigorously, with appropriate controls, replication, and sample sizes. The conclusions must be drawn appropriately based on the data presented.

Reviewer #1: Yes

Reviewer #2: (No Response)

**********

3. Has the statistical analysis been performed appropriately and rigorously?

Reviewer #1: Yes

Reviewer #2: (No Response)

**********

4. Have the authors made all data underlying the findings in their manuscript fully available?

The PLOS Data policy requires authors to make all data underlying the findings described in their manuscript fully available without restriction, with rare exception (please refer to the Data Availability Statement in the manuscript PDF file). The data should be provided as part of the manuscript or its supporting information, or deposited to a public repository. For example, in addition to summary statistics, the data points behind means, medians and variance measures should be available. If there are restrictions on publicly sharing data—e.g. participant privacy or use of data from a third party—those must be specified.

Reviewer #1: Yes

Reviewer #2: (No Response)

**********

5. Is the manuscript presented in an intelligible fashion and written in standard English?

PLOS ONE does not copyedit accepted manuscripts, so the language in submitted articles must be clear, correct, and unambiguous. Any typographical or grammatical errors should be corrected at revision, so please note any specific errors here.

Reviewer #1: Yes

Reviewer #2: (No Response)

**********

6. Review Comments to the Author

Please use the space provided to explain your answers to the questions above. You may also include additional comments for the author, including concerns about dual publication, research ethics, or publication ethics. (Please upload your review as an attachment if it exceeds 20,000 characters)

Reviewer #1: Check format of references. So many mistakes in references , ie 19. Rewrite all references. You can use endnote.

Reviewer #2: (No Response)

**********

7. PLOS authors have the option to publish the peer review history of their article (what does this mean?). If published, this will include your full peer review and any attached files.

If you choose “no”, your identity will remain anonymous but your review may still be made public.

**Do you want your identity to be public for this peer review?**  For information about this choice, including consent withdrawal, please see our Privacy Policy.

Reviewer #1: No

Reviewer #2: No

**********

[NOTE: If reviewer comments were submitted as an attachment file, they will be attached to this email and accessible via the submission site. Please log into your account, locate the manuscript record, and check for the action link "View Attachments". If this link does not appear, there are no attachment files.]

To ensure your figures meet our technical requirements, please review our figure guidelines: https://journals.plos.org/plosone/s/figures

You may also use PLOS’s free figure tool, NAAS, to help you prepare publication quality figures: https://journals.plos.org/plosone/s/figures#loc-tools-for-figure-preparation.

NAAS will assess whether your figures meet our technical requirements by comparing each figure against our figure specifications.

# Author comment: Author response to Decision Letter 2

**DOI:** 10.1371/journal.pone.0353132.r004

9 Jun 2026

ACADEMIC EDITOR – REMARK 1: Check format of references. So many mistakes in references, ie 19. Rewrite all references.

RESPONSE TO ACADEMIC EDITOR: We thank the academic editor for this important comment. We have systematically reviewed the entire reference list and corrected all inconsistencies and formatting errors. All references have been revised to comply with the PLOS One Vancouver style with square brackets. The reference list has been fully standardized and verified for consistency.

REVIEWER 1 – REMARK 1: Check format of references. So many mistakes in references, ie 19. Rewrite all references. You can use endnote.

RESPONSE TO REVIEWER: We thank the reviewer for their comment. See ACADEMIC EDITOR – REMARK 1.

Attachment Submitted filename: Response_to_Reviewers_auresp_2.docx

# Peer review: Decision Letter 2

**DOI:** 10.1371/journal.pone.0353132.r005

**Authors:** Vincenzo Lionetti

19 Jun 2026

Agreement and reliability between the two-day 6-minute incremental step test and two-day cardiopulmonary exercise test in post COVID-19 condition for assessing post-exertional malaise: the REVEAL-study

PONE-D-26-16093R2

Dear Dr. Bomans,

We’re pleased to inform you that your manuscript has been judged scientifically suitable for publication and will be formally accepted for publication once it meets all outstanding technical requirements.

Within one week, you’ll receive an e-mail detailing the required amendments. When these have been addressed, you’ll receive a formal acceptance letter and your manuscript will be scheduled for publication.

An invoice will be generated when your article is formally accepted. Please note, if your institution has a publishing partnership with PLOS and your article meets the relevant criteria, all or part of your publication costs will be covered. Please make sure your user information is up-to-date by logging into Editorial Manager at Editorial Manager® and clicking the ‘Update My Information' link at the top of the page. For questions related to billing, please contact billing support.

If your institution or institutions have a press office, please notify them about your upcoming paper to help maximize its impact. If they’ll be preparing press materials, please inform our press team as soon as possible -- no later than 48 hours after receiving the formal acceptance. Your manuscript will remain under strict press embargo until 2 pm Eastern Time on the date of publication. For more information, please contact onepress@plos.org.

Kind regards,

Vincenzo Lionetti, M.D., PhD

Academic Editor

PLOS One

Additional Editor Comments (optional):

Reviewers' comments:

Reviewer's Responses to Questions

**Comments to the Author**

1. If the authors have adequately addressed your comments raised in a previous round of review and you feel that this manuscript is now acceptable for publication, you may indicate that here to bypass the “Comments to the Author” section, enter your conflict of interest statement in the “Confidential to Editor” section, and submit your "Accept" recommendation.

Reviewer #1: All comments have been addressed

Reviewer #2: All comments have been addressed

**********

2. Is the manuscript technically sound, and do the data support the conclusions?

The manuscript must describe a technically sound piece of scientific research with data that supports the conclusions. Experiments must have been conducted rigorously, with appropriate controls, replication, and sample sizes. The conclusions must be drawn appropriately based on the data presented.

Reviewer #1: Yes

Reviewer #2: (No Response)

**********

3. Has the statistical analysis been performed appropriately and rigorously?

Reviewer #1: Yes

Reviewer #2: (No Response)

**********

4. Have the authors made all data underlying the findings in their manuscript fully available?

The PLOS Data policy requires authors to make all data underlying the findings described in their manuscript fully available without restriction, with rare exception (please refer to the Data Availability Statement in the manuscript PDF file). The data should be provided as part of the manuscript or its supporting information, or deposited to a public repository. For example, in addition to summary statistics, the data points behind means, medians and variance measures should be available. If there are restrictions on publicly sharing data—e.g. participant privacy or use of data from a third party—those must be specified.

Reviewer #1: Yes

Reviewer #2: (No Response)

**********

5. Is the manuscript presented in an intelligible fashion and written in standard English?

PLOS ONE does not copyedit accepted manuscripts, so the language in submitted articles must be clear, correct, and unambiguous. Any typographical or grammatical errors should be corrected at revision, so please note any specific errors here.

Reviewer #1: Yes

Reviewer #2: (No Response)

**********

6. Review Comments to the Author

Please use the space provided to explain your answers to the questions above. You may also include additional comments for the author, including concerns about dual publication, research ethics, or publication ethics. (Please upload your review as an attachment if it exceeds 20,000 characters)

Reviewer #1: (No Response)

Reviewer #2: (No Response)

**********

7. PLOS authors have the option to publish the peer review history of their article (what does this mean?). If published, this will include your full peer review and any attached files.

If you choose “no”, your identity will remain anonymous but your review may still be made public.

**Do you want your identity to be public for this peer review?**  For information about this choice, including consent withdrawal, please see our Privacy Policy.

Reviewer #1: No

Reviewer #2: No

**********

# Editor report: Acceptance letter

**DOI:** 10.1371/journal.pone.0353132.r006

**Authors:** Vincenzo Lionetti

PONE-D-26-16093R2

PLOS One

Dear Dr. Bomans,

I'm pleased to inform you that your manuscript has been deemed suitable for publication in PLOS One. Congratulations! Your manuscript is now being handed over to our production team.

At this stage, our production department will prepare your paper for publication. This includes ensuring the following:

* All references, tables, and figures are properly cited

* All relevant supporting information is included in the manuscript submission,

* There are no issues that prevent the paper from being properly typeset

You will receive further instructions from the production team, including instructions on how to review your proof when it is ready. Please keep in mind that we are working through a large volume of accepted articles, so please give us a few days to review your paper and let you know the next and final steps.

Lastly, if your institution or institutions have a press office, please let them know about your upcoming paper now to help maximize its impact. If they'll be preparing press materials, please inform our press team within the next 48 hours. Your manuscript will remain under strict press embargo until 2 pm Eastern Time on the date of publication. For more information, please contact onepress@plos.org.

You will receive an invoice from PLOS for your publication fee after your manuscript has reached the completed accept phase. If you receive an email requesting payment before acceptance or for any other service, this may be a phishing scheme. Learn how to identify phishing emails and protect your accounts at https://explore.plos.org/phishing.

If we can help with anything else, please email us at customercare@plos.org.

Thank you for submitting your work to PLOS ONE and supporting open access.

Kind regards,

PLOS ONE Editorial Office Staff

on behalf of

Prof. Vincenzo Lionetti

Academic Editor

PLOS One
